# Genome-wide analysis of overlapping genes regulated by iron deficiency and phosphate starvation reveals new interactions in Arabidopsis roots

**DOI:** 10.1186/s13104-015-1524-y

**Published:** 2015-10-12

**Authors:** Wenfeng Li, Ping Lan

**Affiliations:** Collaborative Innovation Center of Sustainable Forestry in Southern China of Jiangsu Province, College of Biology and the Environment, Nanjing Forestry University, Nanjing, 210037 People’s Republic of China; State Key Laboratory of Soil and Sustainable Agriculture, Institute of Soil Science, Chinese Academy of Sciences, Nanjing, 210008 People’s Republic of China

**Keywords:** Iron deficiency, Phosphate deficiency, RNA-seq, Co-expression, Interaction

## Abstract

**Background:**

Iron (Fe) and phosphorus (P) are essential mineral nutrients in plants. Knowledge regarding global changes in the abundance of Fe-responsive genes under Pi deficiency as well as the processes these genes are involved in remains largely unavailable at the genome level. In the current study, we comparatively analyzed RNA sequencing data sets relative to Fe deficiency (NCBI: SRP044814) and Pi starvation (NCBI: SRA050356.1).

**Results:**

Analysis showed a total of 579 overlapping genes that are responsible for both Fe deficiency and Pi starvation in Arabidopsis roots. A subset of 137 genes had greater than twofold changes in transcript abundant as a result of the treatments. Gene ontology (GO) analysis showed that the stress-related processes ‘response to salt stress’, ‘response to oxidative stress’, and ‘response to zinc ion’ were enriched in the 579 genes, while Fe response-related processes, including ‘cellular response to nitric oxide’, ‘cellular response to iron ion’, and ‘cellular iron ion homeostasis’, were also enriched in the subset of 137 genes. Co-expression analysis of the 579 genes using the MACCU toolbox yielded a network consisting of 292 nodes (genes). Further analysis revealed that a subset of 90 genes were up-regulated under Fe shortage, but down-regulated under Pi starvation. GO analysis in this group of genes revealed an increased cellular response to iron ion/nitric oxide/ethylene stimuli. Promoter analysis was performed in 35 of the 90 genes with a 1.5-fold or greater change in abundance, showing that 12 genes contained the PHOSPHATE STARVATION RESPONSE1-binding GNATATNC cis-element within their promoter regions. Quantitative real-time PCR showed that the decreased abundance of Fe acquisition genes under Pi deficiency exclusively relied on Fe concentration in Pi-deficient media.

**Conclusions:**

Comprehensive analysis of the overlapping genes derived from Fe deficiency and Pi starvation provides more information to understand the link between Pi and Fe homeostasis. Gene clustering and root-specific co-expression analysis revealed several potentially important genes which likely function as putative novel players in response to Fe and Pi deficiency or in cross-talk between Fe-deficient responses and Pi-deficient signaling.

**Electronic supplementary material:**

The online version of this article (doi:10.1186/s13104-015-1524-y) contains supplementary material, which is available to authorized users.

## Background

The evolutionary ability of iron (Fe) to change oxidation states between Fe(III) and Fe(II) renders it irreplaceably important in many essential processes associated with basic redox reactions, such as in photosynthesis, respiration and many vital enzymatic reactions [1–5]. Although Fe is abundant in the earth’s crust, it is one of the least available elements for plants in aerobic soils with neutral to basic pH [1–5]. Approximately 30 % of the land worldwide consists of alkaline soils, leading to a demand in bioavailable Fe for plant fitness [1, 5, 6]. As a consequence, Fe deficiency is a major constraint in crop yield and quality [7]. In contrast, in acidic and anaerobic conditions, accumulation of excess Fe is toxic to plant growth and development due to formation of potentially harmful reactive oxygen species (ROS). Plants therefore must tightly regulate cellular Fe homeostasis to allow for effective acquisition, distribution and utilization of Fe [1, 8, 9].

Under Fe-deficient conditions, Arabidopsis (*Arabidopsis thaliana*) and other dicotyledonous and non-graminaceous monocotyledonous plants use a reduction strategy, referred to as strategy I [10], to increase Fe bioavailability. In this strategy, acidification of the rhizosphere mediated by the H^+^-translocating P-type ATPase AHA2 [6, 11] occurs as the first step, which leads to an increase in the concentration of chelated Fe(III). Fe(III) is subsequently reduced to soluble Fe(II) by the root surface-localized ferric chelate reductase FERRIC-REDUCTION OXIDASE2 (FRO2) [12]. Soluble Fe(II) is then transported into epidermal cells by the Fe-REGULATED TRANSPORTER1 (IRT1) [13]. Being the major components of the Fe acquisition system, FRO2 and IRT1 are the major players controlling entry of Fe from the soil into cells. At the transcriptional level, expression of both genes is coordinately regulated by the basic helix-loop-helix (bHLH) transcription factor FER-LIKE Fe DEFICIENCY-INDUCED TRANSCRIPTION FACTOR (FIT), but not the transcription factor POPEYE, which is also involved in Fe homeostasis [14–17]. FIT forms heterodimers with bHLH38 and bHLH39 and positively regulates a subset of Fe-responsive genes, including three key genes required for Fe acquisition [12–14, 18, 19]. Recent studies have shown that the transcription factors bHLH100 and bHLH101, which belong to the Ib sub-group of bHLH proteins, are also involved in Arabidopsis Fe deficiency responses by interacting with FIT [20] or via a FIT-independent manner [21].

Studies have shown that expression of *FRO2* and *IRT1* is tightly controlled both locally and systemically [22, 23]. However, in some cases disrupted Fe signaling in several mutants, such as *frd3* [24, 25], *opt3* [26] and the quadruple nicotianamine synthase mutant *nas4x*-*1* [27] in Arabidopsis, *dgl* and *brz* mutants [28–30] in pea (*Pisum sativum*) and the *chloronerva* mutant *chln* [31] in tomato (*Solanum lycopersicum*), constitutively activates expression of Fe acquisition genes even under sufficient Fe conditions. By contrast, *FRO2* and *IRT1* expression has been documented to be decreased under phosphate (Pi)-deficient conditions [32–36]. Currently, the predominate explanation for decreased expression of Fe acquisition genes under Pi-deficient conditions is that Pi deficiency results in enhanced Fe accessibility to plants in the media, which leads to an over accumulation of Fe in plants, subsequently causing down-regulated expression of Fe-responsive genes. However, if the Pi-deficient media without available Fe or with low concentrations of Fe, does the down-regulated expression of Fe-responsive genes occur? A recent report showed that PHOSPHATE STARVATION RESPONSE1 (PHR1), a major regulator of the Pi deficiency response, could bind the promoter of the Fe storage gene *Ferritin1* through the imperfect palindromic sequence motif P1BS (PHR1 binding sequences, GNATATNC), strongly supporting the link between Fe and Pi homeostasis [37]. However, it remains an open question whether this link exists or not in *phr1* mutant plants.

Moreover, although down-regulation of Fe deficient-induced Fe acquisition genes under Pi deficient conditions has been documented [33, 38, 39], knowledge regarding genome-wide transcriptional changes of Fe-responsive genes under Pi deficiency remains unavailable, and the processes of the genes involved are largely unknown. To provide systemic information about transcriptional changes in Fe-responsive genes under Pi deficiency and to further extend knowledge of the relationship between Fe and Pi at the transcript level, we mined and re-analyzed previous RNA sequencing (RNA-seq) data sets relative to transcriptome profiling in Fe-deficient [40] and Pi-deficient Arabidopsis roots [36], with an emphasis on 579 overlapping genes that respond to both Fe and Pi deficiency. We revealed that a subset of 137 genes had a twofold or greater change in abundance under either of the treatments. A subset of 90 genes with an increased abundance under Fe deficiency, but a decrease under Pi deficiency, may be critical for Fe responses under Pi-deficient conditions. By gene clustering and root-specific co-expression analysis, we revealed several potentially important genes that likely function as putative novel players in response to Fe and Pi deficiency or in the cross talk between Fe deficient responses and phosphate-deficient signaling, which may be determined in follow-up experiments. Finally, we found that FIT-regulated genes were down-regulated by Pi deficiency, and an extent of Fe in the Pi deficient media is required for this down-regulation, suggesting that, besides FIT, PHR1, Fe itself might be a critical factor involved in the transcriptional regulation under both Pi- and Fe-deficiency.

## Results

### Genes responsible for Fe and Pi deficiency in Arabidopsis roots

**Fig. 1:**
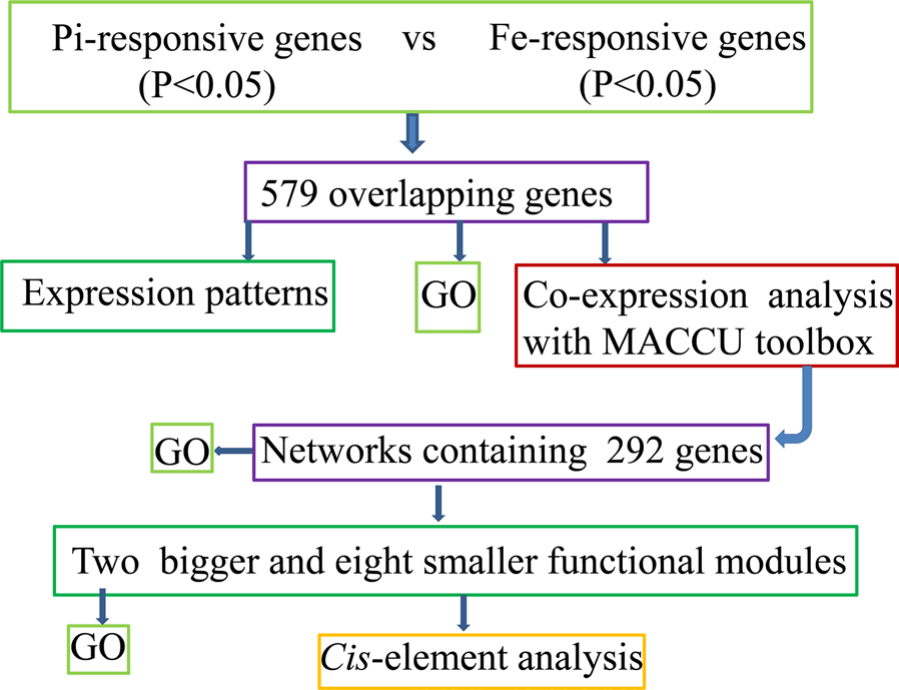
Bioinformatic analysis scheme of the 579 differentially expressed overlapping genes regulated by both iron and phosphate deficiency in Arabidopsis roots

**Fig. 2:**
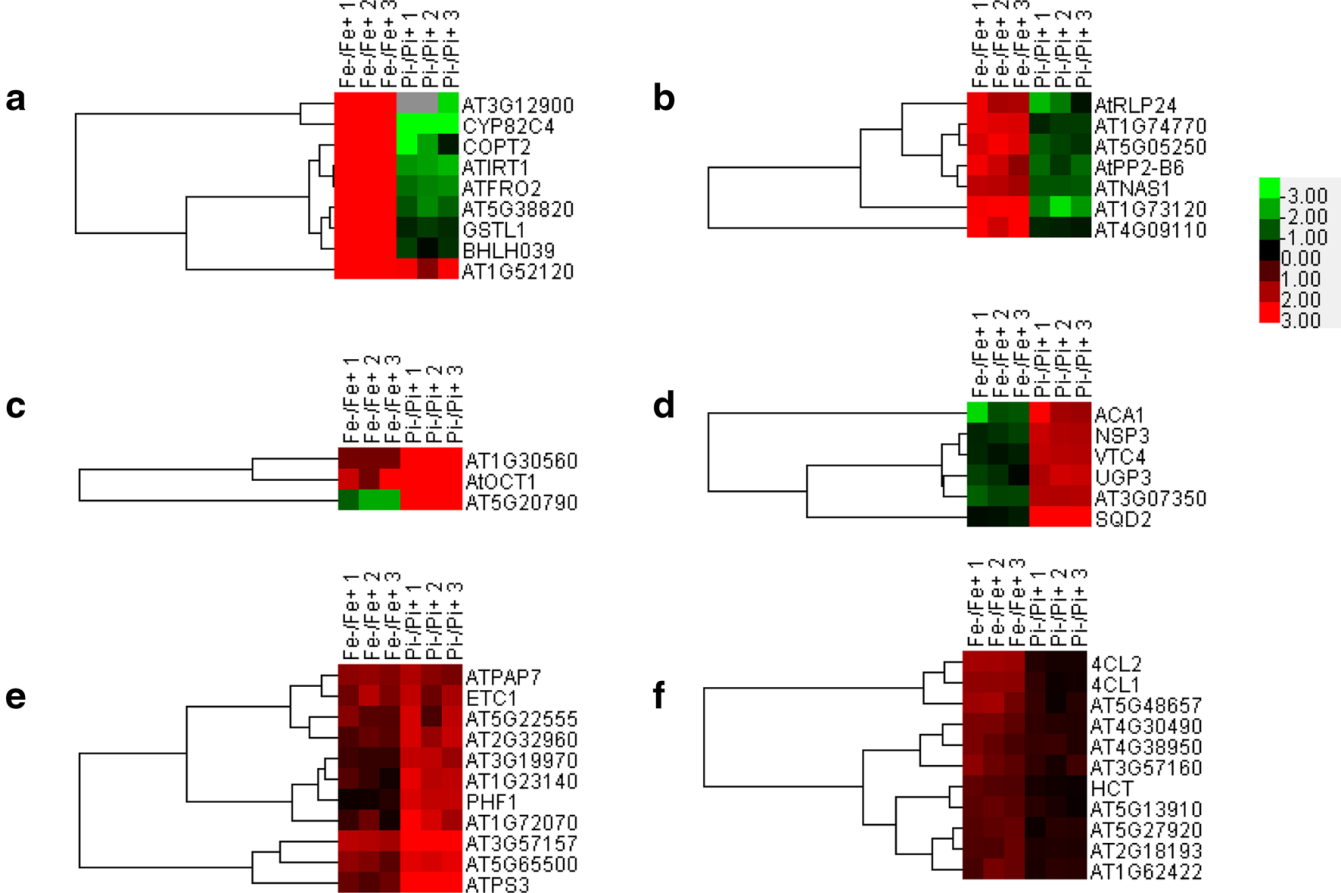
Hierarchical cluster analysis of 137 overlapping genes with greater than twofold changes in transcript abundance in Arabidopsis roots grown under Fe or Pi deficiency. **a**–**f** Indicate six representative sub-clusters. Complete clustering results of the 137 overlapping genes can be found in Additional file 2. Results shown are parts of the representative clusters from Additional file 2. Fold change in transcript abundance was defined as transcript level (reads per kilobase per million mapped reads) in Fe-deficient (Fe−) conditions divided by the level in normal conditions (Fe+), with three biological repeats. The same strategy was applied to Pi treatment. The *color key* indicates log2 transformed intensity; *grey* indicates that the number is missing

Previously published RNA-seq data sets [36, 40] relative to Fe and Pi deficiency in Arabidopsis roots were re-analyzed, and differentially expressed genes (P < 0.05) upon Fe deficiency were compared with those (P < 0.05) exposed to Pi deficiency. Subsequent analyses focused on the 579 overlapping genes (Additional file l) as shown in Fig. 1. Of the 579 genes, 137 showed an increase or decrease in transcript abundance, with fold changes greater than twofold under either of the treatments (Table 1; Additional file 2). Fe acquisition genes *FRO2* and *IRT1,* copper transporter *COPT2,* Fe(II)-dependent oxygenase gene AT3G12900, cytochrome P450 *CYP82C4* (AT4G31940), mannose-binding lectin protein gene AT1G52120, glutathione transferase lambda 1 *GSTL1* and amino acid transporter gene AT5G38820 showed the strongest induction under Fe deficiency and were up-regulated by more than 50-fold (Table 1; Fig. 2a). Excluding AT1G52120, these genes were among the most repressed under Pi deficiency and were down-regulated by two to more than tenfold (Table 1; Fig. 2a). Genes encoding transcriptional factor bHLH039, ZIP9, zinc binding protein (AT1G74770), ATROPGEF10, receptor like protein 24 RLP24, phloem protein 2-B6 and other functionally unknown proteins were among the second group of highly induced genes following Fe deficiency and were up-regulated by more than fivefold (Table 1; Fig. 2a, b). The most induced genes following Pi deficiency were *AtOCT1*, an unknown protein gene AT5G20790 and a major facilitator protein gene AT1G30560, which were induced by more than 50-fold (Table 1; Fig. 2c). Highly induced genes under Pi deficiency were *ATPS3* (phosphate starvation-induced gene 3), *SQD2* (sulfoquinovosyl diacylglycerol 2) and U-box domain-containing protein kinase gene AT5G65500, with changes more than fivefold (Table 1; Fig. 2d, e). Interestingly, a subset of genes involved in lignin biosynthesis was induced by both Fe and Pi deficiency (Table 1; Fig. 2f).

**Table 1:** Subset of 137 of the 579 overlapping genes with more than twofold changes in transcript abundance due to either of the treatments

AGI	Annotation	Mean (−Fe/+Fe)	SD	Mean (−Pi/+Pi)	SD
At3G12900	2-Oxoglutarate (2OG) and Fe(II)-dependent oxygenase superfamily protein	612.44	199.47	0.06	0.10
At4G31940	CYP82C4, cytochrome P450, family 82, subfamily C, polypeptide 4	184.70	3.63	0.08	0.01
At1G52120	Mannose-binding lectin superfamily protein	157.65	59.83	6.13	2.70
At3G46900	COPT2, copper transporter 2	71.15	33.39	0.40	0.36
At1G01580	ATFRO2, FRD1, FRO2, ferric reduction oxidase 2	59.73	10.56	0.35	0.05
At5G02780	GSTL1, glutathione transferase lambda 1	57.98	10.87	0.69	0.06
At4G19690	ATIRT1, IRT1, iron-regulated transporter 1	54.72	7.88	0.26	0.04
At5G38820	Transmembrane amino acid transporter family protein	54.35	11.69	0.43	0.09
At3G56980	BHLH039, ORG3, basic helix-loop-helix (bHLH) DNA-binding superfamily protein	34.23	13.48	0.73	0.18
At3G13610	2-Oxoglutarate (2OG) and Fe(II)-dependent oxygenase superfamily protein	10.04	0.65	1.63	0.25
At1G73120	Unknown protein	8.98	2.09	0.28	0.11
At1G73220	1-Oct, AtOCT1, organic cation/carnitine transporter1	7.38	6.18	196.69	139.63
At4G33020	ATZIP9, ZIP9, ZIP metal ion transporter family	7.09	2.01	2.21	0.97
At5G05250	Unknown protein	6.62	0.95	0.57	0.10
At3G61410	BEST Arabidopsis thaliana protein match is: U-box domain-containing protein kinase family protein (TAIR:AT2G45910.1)	6.53	1.32	3.79	0.43
At1G74770	Zinc ion binding	6.43	0.50	0.66	0.07
At5G19560	ATROPGEF10, ROPGEF10, ROP uanine nucleotide exchange factor 10	5.33	0.51	2.12	0.20
At2G02310	AtPP2-B6, PP2-B6, phloem protein 2-B6	5.30	2.01	0.49	0.12
At2G33020	AtRLP24, RLP24, receptor like protein 24	5.05	1.74	0.48	0.33
At3G59880	Unknown protein	4.58	2.42	2.51	0.54
At5G01060	Protein kinase protein with tetratricopeptide repeat domain	4.44	0.17	1.70	0.10
At5G04950	ATNAS1, NAS1, nicotianamine synthase 1	4.39	0.50	0.49	0.01
At3G57157	Other RNA	4.28	0.37	9.81	1.46
At3G60330	AHA7, HA7, H(+)-ATPase 7	4.27	0.69	1.79	0.21
At3G21500	DXPS1, 1-deoxy-d-xylulose 5-phosphate synthase 1	3.76	1.94	0.40	0.26
At3G21240	4CL2, AT4CL2, 4-coumarate:CoA ligase 2	3.73	0.16	1.25	0.09
At3G50710	F-box/RNI-like/FBD-like domains-containing protein	3.57	1.09	2.71	1.32
At1G18910	Zinc ion binding	3.33	0.13	0.78	0.04
At5G48657	Defense protein-related	3.33	0.71	1.36	0.23
At1G01380	ETC1, Homeodomain-like superfamily protein	3.22	1.09	3.73	1.22
At1G51680	4CL.1, 4CL1, AT4CL1, 4-coumarate:CoA ligase 1	3.22	0.06	1.27	0.19
At5G54790	Unknown protein	3.20	0.75	2.02	0.19
At2G01880	ATPAP7, PAP7, purple acid phosphatase 7	3.14	0.21	3.31	0.81
At5G19970	Unknown protein	2.81	0.43	0.57	0.07
At5G65500	U-box domain-containing protein kinase family protein	2.73	0.60	6.26	0.42
At4G12735	Unknown protein	2.67	1.20	1.87	0.47
At3G57160	Unknown protein	2.57	0.55	1.46	0.23
At5G26820	ATIREG3, IREG3, IREG3, MAR1, RTS3, iron-regulated protein 3	2.54	0.28	0.77	0.19
At4G30490	AFG1-like ATPase family protein	2.52	0.32	1.40	0.08
At1G78230	Outer arm dynein light chain 1 protein	2.51	0.57	0.59	0.22
At3G18290	BTS, EMB2454, zinc finger protein-related	2.51	0.14	0.76	0.09
At1G51870	Protein kinase family protein	2.42	0.42	2.25	0.83
At5G22555	Unknown protein	2.37	0.58	4.13	2.07
At1G53310	ATPEPC1, ATPPC1, PEPC1, PPC1, phosphoenolpyruvate carboxylase 1	2.34	0.19	2.21	0.25
At3G51570	Disease resistance protein (TIR-NBS-LRR class) family	2.32	0.58	2.70	1.18
At5G22890	C2H2 and C2HC zinc fingers superfamily protein	2.32	0.53	1.91	0.25
At4G22980	Pyridoxal phosphate (PLP)-dependent transferases superfamily protein (TAIR:AT5G51920.1)	2.30	0.28	0.56	0.14
At1G14190	Glucose-methanol-choline (GMC) oxidoreductase family protein	2.29	0.41	0.78	0.08
At1G48300	Unknown protein	2.27	0.25	0.85	0.07
At1G24320	Six-hairpin glycosidases superfamily protein	2.26	0.27	0.78	0.09
At1G62422	Unknown protein	2.25	0.43	1.37	0.10
At4G38950	ATP binding microtubule motor family protein	2.25	0.45	1.50	0.15
At3G47420	ATPS3, PS3, phosphate starvation-induced gene 3	2.24	0.27	12.82	5.53
At5G26320	TRAF-like family protein	2.24	0.26	2.95	0.37
At2G43570	CHI, chitinase, putative	2.22	0.87	2.26	0.45
At4G26890	MAPKKK16, mitogen-activated protein kinase kinase kinase 16	2.16	0.42	3.00	0.85
At5G13910	LEP, Integrase-type DNA-binding superfamily protein	2.14	0.15	1.25	0.16
At5G27920	F-box family protein	2.14	0.15	1.29	0.15
At3G15510	ANAC056, ATNAC2, NAC2, NARS1, NAC domain containing protein 2	2.13	0.62	0.71	0.15
At5G53850	Haloacid dehalogenase-like hydrolase family protein	2.09	0.06	0.96	0.01
At2G18193	P-loop containing nucleoside triphosphate hydrolases superfamily protein	2.07	0.26	1.29	0.01
At2G32960	Phosphotyrosine protein phosphatases superfamily protein	2.07	0.23	4.79	1.23
At1G64590	NAD(P)-binding Rossmann-fold superfamily protein	2.06	0.13	2.39	0.70
At5G48930	HCT, hydroxycinnamoyl-CoA shikimate/quinate hydroxycinnamoyl transferase	2.04	0.17	1.16	0.07
At2G14210	AGL44, ANR1, AGAMOUS-like 44	2.01	0.37	0.82	0.09
At5G20790	Unknown protein	0.32	0.13	90.27	24.26
At1G30560	Major facilitator superfamily protein	–	–	55.93	32.45
At5G01220	SQD2, sulfoquinovosyldiacylglycerol 2	0.86	0.06	10.49	3.02
At1G72070	Chaperone DnaJ-domain superfamily protein	1.72	0.62	6.60	3.34
At3G52720	ACA1, ATACA1, CAH1, alpha carbonic anhydrase 1	0.40	0.20	5.56	3.11
At1G23140	Calcium-dependent lipid-binding (CaLB domain) family protein	1.58	0.43	5.33	1.19
At3G52190	PHF1, phosphate transporter traffic facilitator1	1.22	0.12	5.25	0.61
At3G56040	UGP3, UDP-glucose pyrophosphorylase 3	0.74	0.18	4.98	0.71
At3G02870	VTC4, Inositol monophosphatase family protein	0.79	0.06	4.64	0.29
At3G16390	NSP3, nitrile specifier protein 3	0.67	0.08	4.55	0.59
At3G07350	Protein of unknown function (DUF506)	0.54	0.07	4.40	0.09
At3G19970	Alpha/beta-hydrolases superfamily protein	1.58	0.08	4.37	0.76
At1G15040	Class I glutamine amidotransferase-like superfamily protein	0.60	0.04	3.73	1.12
At3G12500	ATHCHIB, B-CHI, CHI-B, HCHIB, PR-3, PR3, basic chitinase	0.52	0.05	3.05	0.75
At1G18970	GLP4, germin-like protein 4	0.71	0.12	3.04	0.41
At2G29000	Leucine-rich repeat protein kinase family protein	1.85	0.60	3.03	0.79
At3G53620	AtPPa4, PPa4, pyrophosphorylase 4	0.82	0.03	2.96	0.19
At1G14220	Ribonuclease T2 family protein	0.74	0.18	2.89	0.27
At4G32480	Protein of unknown function (DUF506)	1.35	0.19	2.85	1.36
At3G06962	Other RNA	1.82	0.49	2.74	0.44
At1G11920	Pectin lyase-like superfamily protein	–	–	2.68	0.51
At1G08650	ATPPCK1, PPCK1, phosphoenolpyruvate carboxylase kinase 1	1.77	0.16	2.58	0.21
At4G04040	MEE51, Phosphofructokinase family protein	0.83	0.04	2.50	0.22
At5G57540	AtXTH13, XTH13, xyloglucan endotransglucosylase/hydrolase 13	1.53	0.11	2.33	0.36
At5G40860	Unknown protein	1.51	0.11	2.32	0.81
At1G68740	PHO1;H1, EXS (ERD1/XPR1/SYG1) family protein	0.39	0.08	2.31	0.44
At3G32040	Terpenoid synthases superfamily protein	1.32	0.21	2.30	0.11
At2G25240	Serine protease inhibitor (SERPIN) family protein	1.53	0.15	2.27	0.08
At2G16430	ATPAP10, PAP10, purple acid phosphatase 10	0.68	0.08	2.26	0.33
At4G30670	Putative membrane lipoprotein	1.61	0.16	2.22	0.17
At3G10420	P-loop containing nucleoside triphosphate hydrolases superfamily protein	1.24	0.08	2.20	0.35
At2G42600	ATPPC2, PPC2, phosphoenolpyruvate carboxylase 2	0.77	0.05	2.18	0.09
At4G11650	ATOSM34, OSM34, osmotin 34	0.67	0.10	2.16	0.38
At2G23960	Class I glutamine amidotransferase-like superfamily protein	1.55	0.27	2.13	0.21
At1G05300	ZIP5, zinc transporter 5 precursor	0.49	0.05	2.13	0.11
At3G13110	ATSERAT2;2, SAT-1, SAT-A, SAT-M, SAT3, SERAT2;2, serine acetyltransferase 2;2	0.83	0.08	2.12	0.22
At2G22290	ATRAB-H1D, ATRAB6, ATRABH1D, RAB-H1D, RABH1d, RAB GTPase homolog H1D	1.91	0.66	2.10	0.59
At4G20160	RING/U-box superfamily protein (TAIR:AT1G30860.1)	1.71	0.10	2.07	0.17
At1G20390	Transposable element gene	0.56	0.11	2.07	0.04
At5G20280	ATSPS1F, SPS1F, sucrose phosphate synthase 1F	0.77	0.06	2.05	0.17
At5G01870	Bifunctional inhibitor/lipid-transfer protein/seed storage 2S albumin superfamily protein	0.71	0.10	2.05	0.51
At3G05858	Unknown protein	1.64	0.09	2.01	0.47
At2G17280	Phosphoglycerate mutase family protein	1.16	0.02	2.01	0.47
At1G26250	Proline-rich extensin-like family protein	0.55	0.16	2.00	0.82
At4G09110	RING/U-box superfamily protein	–	–	0.77	0.04

Change in gene expression is shown as the mean and standard deviation (SD)

No change is indicated as “−” in cases when a gene transcript was not determined (read number = zero) in any of the biological replicates under control conditions

Gene ontology (GO) analysis of the 579 overlapping genes revealed that stress-related processes, including ‘response to salt stress’, ‘response to oxidative stress’ and ‘response to zinc ion’, were enriched (Additional file 3), while analysis of the subset of 137 genes showed that Fe response-related processes, including ‘cellular response to nitric oxide’, ‘cellular response to Fe ion’ and ‘cellular Fe ion homeostasis’, were also enriched (Additional file 4).

### Gene expression patterns of overlapping genes

**Fig. 3:**
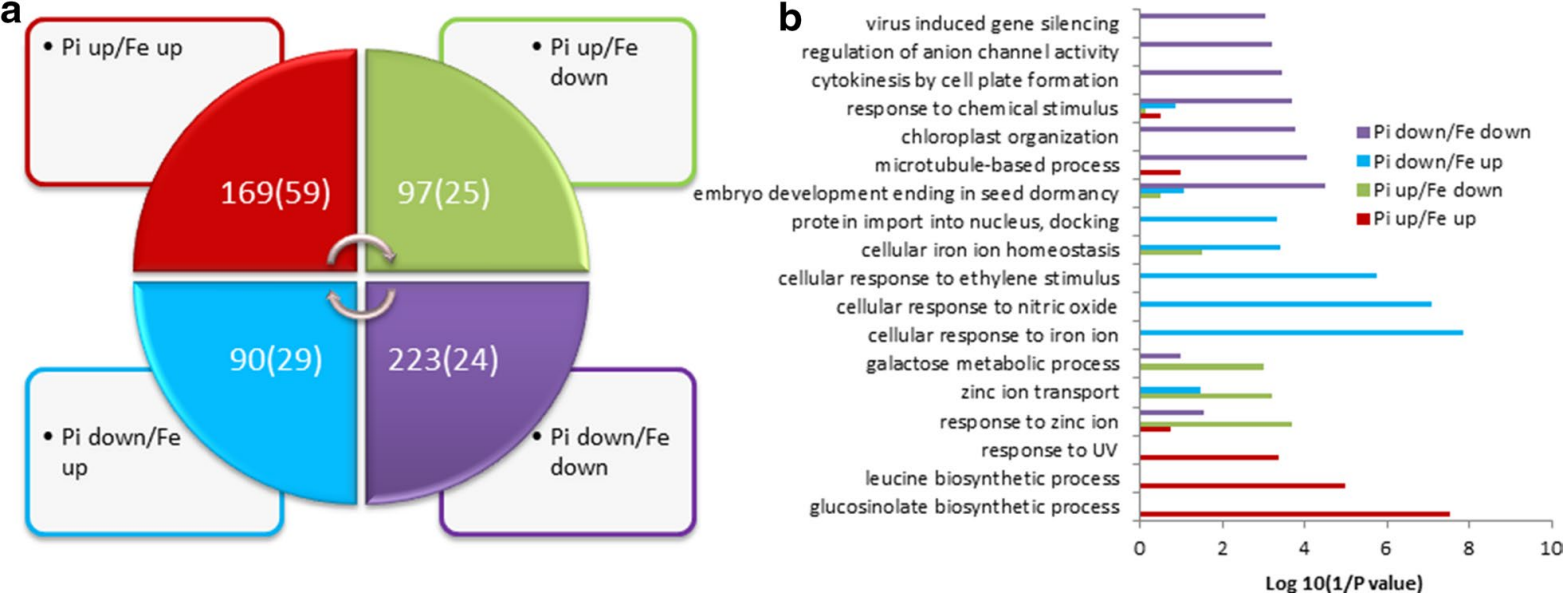
Expression patterns (**a**) and differential gene ontology (GO) enrichment analysis of the four types of 579 overlapping genes (**b**). *Bracketed numbers* in **a** indicate the number of genes with greater than a twofold change in transcript level under either of the stresses

Expression patterns of the 579 overlapping genes were divided into four types according to changes at the transcript level under two stress conditions (Fig. 3a). Type one was composed of 223 genes (of which 24 genes were down-regulated by more than twofold) with decreased transcription under both Pi and Fe deficiency. GO analysis of this group of genes revealed that the processes of ‘embryo development ending in seed dormancy’, ‘microtubule-based process’ and ‘chloroplast organization’, were most enriched (Fig. 3b). In contrast, transcript abundance of 169 genes in the type two category were shown to be increased following both Pi and Fe deficiency, with processes of ‘glucosinolate and leucine biosynthesis’ and ‘UV response’ being enriched (Fig. 3b). Type three was composed of 97 genes with increased transcript abundance under Pi deficiency, but decreased under Fe deficiency. In contrast, transcript abundance of the 90 genes in type four were decreased under Pi deficiency and increased under Fe deficiency. GO enrichment analysis showed that the zinc-related processes ‘response to zinc ion’, ‘zinc ion transport’ and ‘galactose metabolic process’ were enriched in type three (Fig. 3b). Iron-related processes ‘cellular response to Fe ion’, ‘cellular response to nitric oxide’, ‘cellular response to ethylene stimulus’, ‘cellular Fe ion homeostasis’ and ‘protein import into nucleus’ were enriched in type four (Fig. 3b).

### Identification of overlapping gene modules by co-expression analysis

Stress-specific variability in gene expression may occur at the individual gene level, but can also occur in a coordinated manner. To determine functional modules, co-expression networks (i.e., groups of genes that show similar expression patterns under diverse conditions) of the 579 overlapping genes were generated using MACCU software [41]. Pairwise co-expressed genes were selected with a Pearson correlation coefficient cutoff of 0.7 [36, 41]. The 300 publicly available microarrays that were mined for co-expression analysis discriminated between root-related experiments. As such, the co-expression relationships reported herein are restricted to roots [42–44]. Co-expression relationships between these genes were visualized using Cytoscape (http://www.cytoscape.org). This analysis yielded a network composed of 292 nodes (genes) and 1595 edges (correlations between genes; Additional file 5). The network can be further divided into two large and eight small clusters (modules). The largest module was composed of 210 genes, most of which are associated with stress (Additional file 6). GO enrichment analysis revealed that the biological processes ‘glucosinolate biosynthetic process’, ‘response to cadmium ion’, ‘response to salt stress’ and ‘leucine biosynthetic process’ were most enriched in this module (Additional file 7). Via connection to the zinc binding protein gene AT1G74770, two marker genes strongly induced by Fe deficiency, *IRT1* (AT4G19690) and *CYP82C4* (AT4G31940), were associated with this module.

**Fig. 4:**
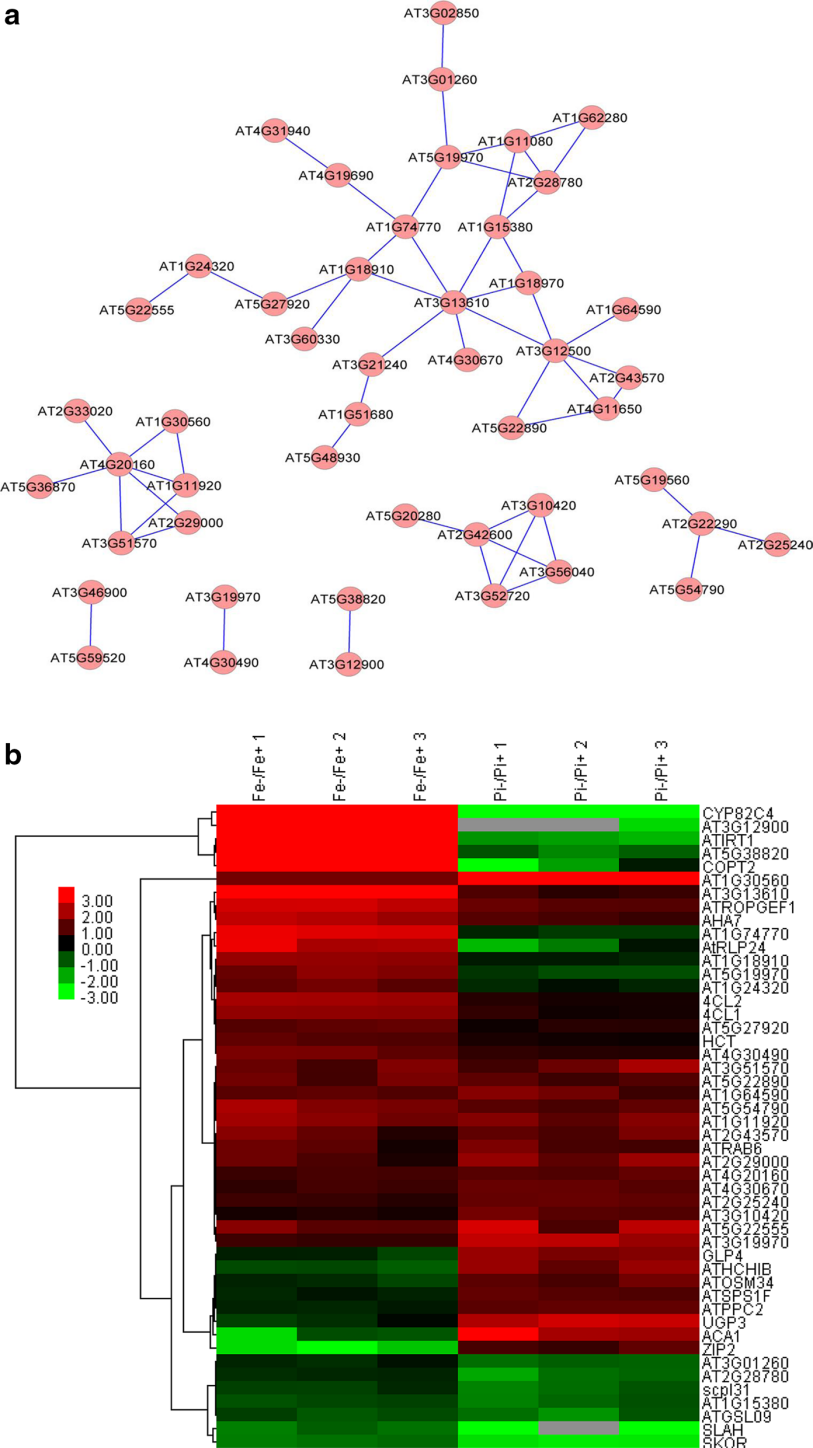
Co-expression relationships of the 137 differentially expressed genes with changes greater than twofold (**a**), and heat map of genes involved in network construction (**b**)

Co-expression analysis of the subset of 137 overlapping genes with changes greater than twofold yielded a network consisting of 48 nodes and 56 edges. The Fe deficiency-regulated marker genes *IRT1* and *CYP82C4* (AT4G31940) remained in the network (Fig. 4a). This network can be divided into one large (26 genes) and six small clusters (Fig. 4a). Detailed expression information of these genes upon Fe or Pi deficiency is shown in Fig. 4b. GO enrichment analysis of the genes involved in the co-expression network revealed that the biological processes ‘cellular response to nitric oxide’, ‘cellular response to Fe ion’ and ‘cellular response to ethylene stimulus’ were enriched (Table 2).

**Table 2:** Gene ontology enrichment was assessed using GOBUin the 48 genes in the large sub-network shown in Fig. 4a (elim, P < 0.01)

GOID	*P* value (elim)	GO name
GO: 0071732	1.51E−06	Cellular response to nitric oxide
GO: 0071281	9.74E−06	Cellular response to iron ion
GO: 0009620	1.11E−04	Response to fungus
GO: 0071369	1.52E−04	Cellular response to ethylene stimulus
GO: 0006829	1.54E−04	Zinc ion transport
GO: 0015794	0.001428	Glycerol-3-phosphate transport
GO: 0015678	0.001428	High-affinity copper ion transport
GO: 0010421	0.002855	Hydrogen peroxide-mediated programmed cell death
GO: 0009805	0.004279	Coumarin biosynthetic process
GO: 0009871	0.004279	Jasmonic acid and ethylene-dependent systemic resistance, ethylene mediated signaling pathway
GO: 0009411	0.004536	Response to UV
GO: 0006873	0.005599	Cellular ion homeostasis
GO: 0009963	0.007122	Positive regulation of flavonoid biosynthetic process
GO: 0009311	0.007707	Oligosaccharide metabolic process
GO: 0006828	0.009958	Manganese ion transport

**Fig. 5:**
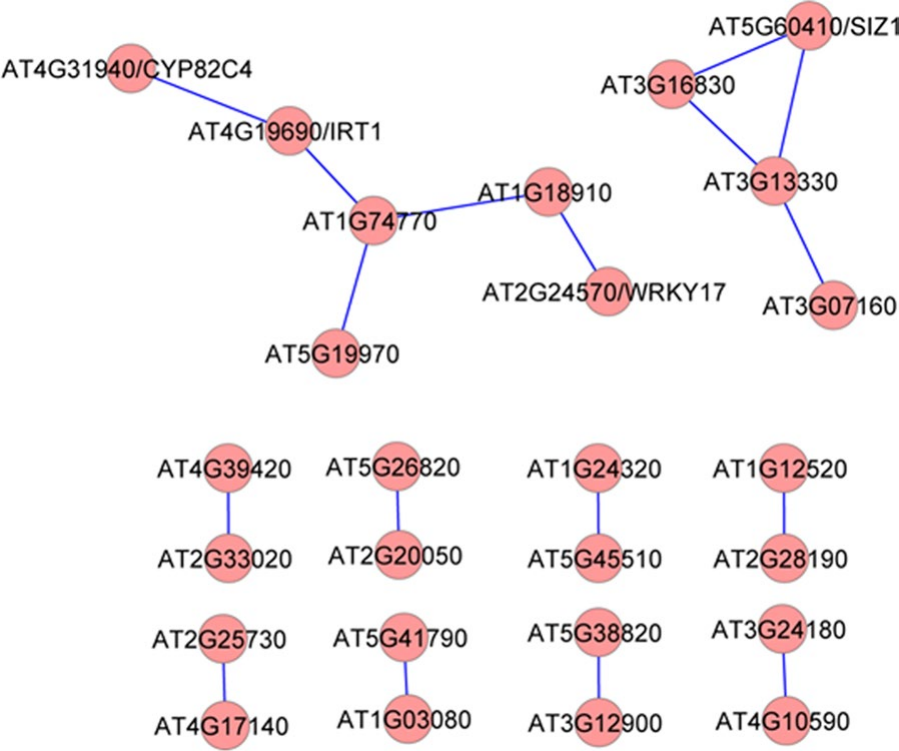
Co-expression relationships of the 90 differentially expressed overlapping genes induced by Fe deficiency but repressed by Pi starvation

To search for potentially functional novel modules, co-expression analysis was applied to the subset of 90 genes that were induced by Fe deficiency, but down-regulated by Pi deficiency (Additional file l). A network containing 26 nodes and 17 edges was created using the same criteria (Fig. 5). The network can be divided into 10 small clusters (none with more than ten nodes), with the largest one containing several Fe-responsive marker genes and one transcriptional factor *WRKY 17* (Fig. 5). The second largest cluster was composed of four genes, including the Pi homeostasis regulator *SIZ1* (Fig. 5). For 97 genes induced by Pi deficiency but repressed by Fe deficiency, co-expression analysis resulted in a network containing 26 nodes and 29 edges that were divided into one large and two small clusters (Additional file 8).

### Analysis of P1BS motif in 35 genes induced by Fe deficiency but down regulated by Pi deficiency

A subset of 35 genes in the type four category had an increase in transcript abundance under Fe deficiency but a decrease under Pi deficiency, with changes more than 1.5-fold (Additional file 9). A 3000 bp sequence upstream of the translation start (named −3000 bp) of these 35 genes was retrieved from TAIR10 and used to search the PHR1 recognition sequence 5′-GNATATNC-3′ (P1BS motif). Results showed that 12 of the 35 genes contained at least one P1BS motif, and seven of the 12 genes contained the P1BS motif within −1000 bp of their promoter regions (Table 3). In total, 11 P1BS patterns were hit in the 12 genes, with 5′-GTATATGC-3′ and 5′-GTATATTC-3′ being the most frequent (5 and 3 hits out of 18 total hits, respectively).

**Table 3:** Distribution of the P1BS motif in promoter regions of 12 genes

AGI	Matching positions		Hit pattern (5′–3′)
	Start	End	
AT1G01580	2660	2667	GTATATTC
AT1G01580	2701	2708	GTATATTC
AT1G18910	134	141	GGATATCC
AT1G18910	359	366	GTATATAC
AT1G18910	1311	1318	GTATATGC
AT1G24320	2052	2059	GCATATCC
AT1G48300	1167	1174	GCATATTC
AT2G02310	645	652	GCATATAC
AT3G12900	445	452	GTATATTC
AT3G18290	129	136	GTATATAC
AT3G18290	703	710	GCATATGC
AT3G56980	153	160	GTATATGC
AT3G56980	2484	2491	GTATATGC
AT3G56980	2484	2491	GTATATGC
AT4G00910	2514	2521	GTATATGC
AT4G19690	838	845	GAATATCC
AT4G22980	1641	1648	GAATATAC
AT5G02780	369	376	GAATATGC

### Down-regulation of Fe-acquisition genes upon Pi deficiency is dependent on Fe concentration in the media

**Fig. 6:**
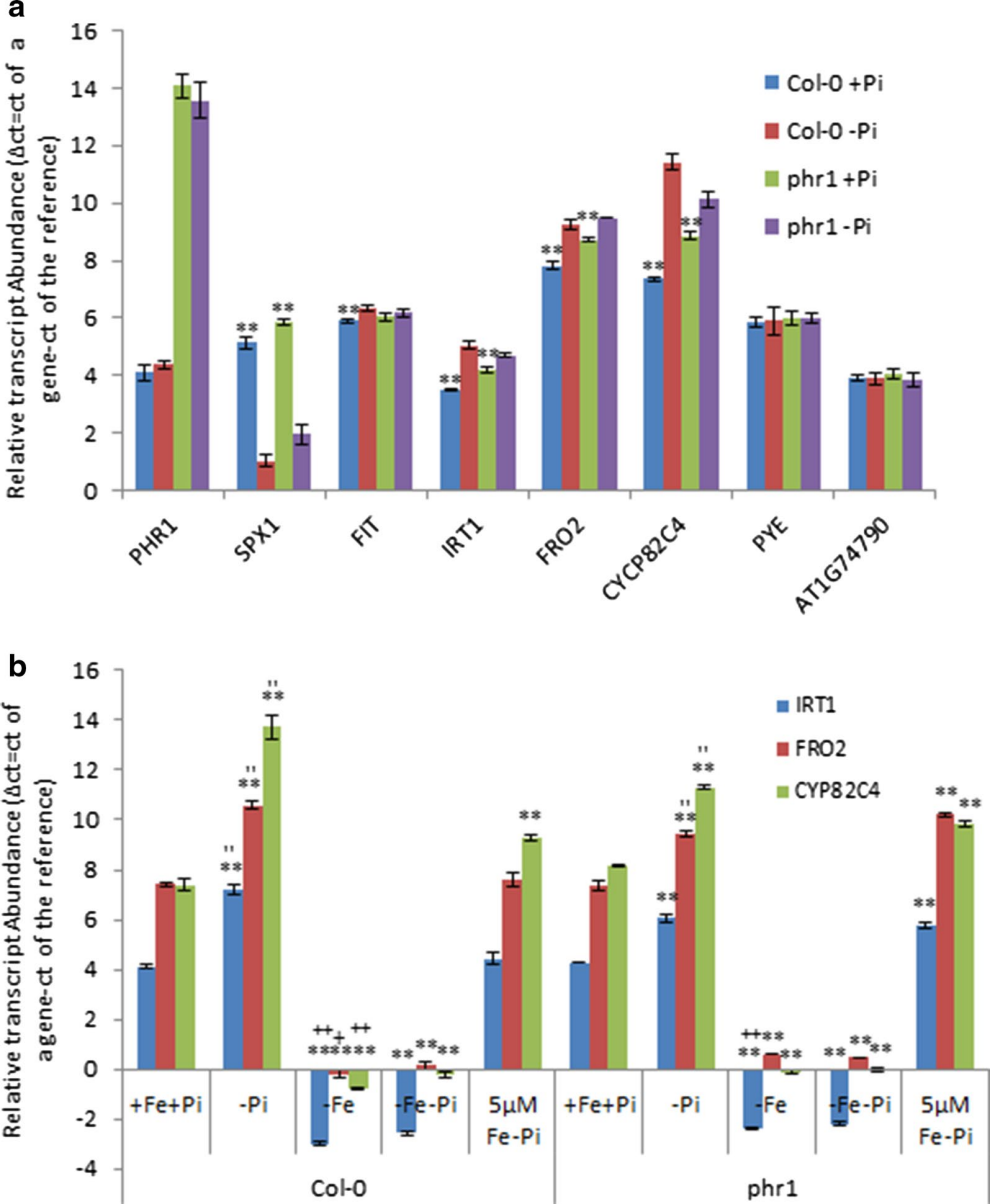
Reverse transcription–quantitative PCR (RT–qPCR) detected expression of Pi- and Fe-responsive marker genes under Pi deficiency (**a**) or Fe deficiency or Pi deficiency without or with low Fe concentrations (**b**). Total RNA was isolated from roots in wild type or *phr1* mutant plants and qPCR was performed. Expression levels are relative to normal controls. *Error bars* represent SD of biological replicates from three independent experiments. Data significantly different from the corresponding controls are indicated (‘−Pi’ versus ‘+Pi’, *P < 0.05, **P < 0.01; ‘−Fe’ versus ‘−Fe−Pi’, +P < 0.05, ++P < 0.01; ‘−Pi’ versus ‘5 µMFe−Pi’, ′P < 0.05, ″P < 0.01; Student’s *t* test)

To determine how Fe acquisition genes are down-regulated by Pi deficiency and whether this down-regulation is dependent on PHR1, we investigated changes in genes that were most induced by Fe deficiency (including the two Fe acquisition genes *FRO2* and *IRT1*) at the transcript level in wild type and the *phr1* mutant under varied growth conditions as follows: Pi deficiency (−Pi, in which the concentration of Fe was 40 µM), Fe deficiency (−Fe), both Pi- and Fe-deficient (−Pi−Fe), Pi deficiency with low Fe concentration (−Pi + 5 µM Fe) and control conditions (+Pi+Fe). Null expression of *PHR1* in the *phr1* mutant was first verified by quantitative real-time PCR (qPCR) (the ct value of the reference is around 20 cycles while the ct value of the *PHR1* is around 34 cycles in the *phr1* mutant plants). As shown in Fig. 6a and in agreement with previously reported results [45], transcriptional expression of *PHR1* was not significantly regulated by Pi deficiency in wild type plants and could not be detected in *phr1* mutant plants under both Pi sufficient and deficient conditions. As a control, the expression of *SPX1* [46, 47], a Pi-responsive marker gene, was significantly induced by Pi deficiency. Consistent with our transcriptomic data, transcriptional expression of the Fe acquisition genes *IRT1* and *FRO2* as well as the Fe deficiency-induced marker gene *CYP82C4* was significantly down-regulated under Pi deficiency in both *Col*-*0* and *phr1* roots (Fig. 6a). Because all these Fe-responsive genes tested were mainly regulated by transcription factor FIT [14], we thus tested whether the expression of FIT itself was affected or not by Pi deficiency. As shown in Fig. 6a, the expression level of FIT was significantly lower in Pi-deficient roots than in Pi-sufficient roots in wild type plants. In addition to FIT, another transcription factor PYE [34], regulating the expression of another subset of Fe-responsive genes, has been reported to be required for plant Fe homeostasis. However, both *PYE* and its target AT1G74790 were not affected by Pi deficiency (Fig. 6a). To determine whether this down-regulation is dependent on Fe concentrations in the media, we compared transcript abundance under Pi deficiency with different Fe concentrations in both wild type and mutant plants. In wild type plant roots, all genes evaluated were dramatically induced under Fe deficiency (−Fe) but repressed under Pi deficiency (−Pi) compared to expression under control conditions (+Fe+Pi). Fe deficient-induced up-regulation was not blocked but attenuated by the absence of Pi in Fe-deficient media (−Fe−Pi), while Pi deficient-induced down-regulation was dramatically attenuated by 5 µM Fe (low Fe concentration) in the media (Fig. 6b). Similar to results in wild type plants, these genes were significantly induced under Fe deficiency and Fe and Pi deficiency (−Fe−Pi) in the *phr1* mutant roots (Fig. 6b). However, down-regulation of gene expression under Pi deficiency was not all significantly attenuated by 5 µM Fe in Pi-deficient media in the *phr1* mutant roots (Fig. 6b).

## Discussion

As an essential element for all living organisms, particularly as a major constraint in crop yield and quality, Fe deficiency responses in plants have been extensively studied in the last decade [1, 6, 9]. With the emergence of high throughput research platforms, many genes and proteins have been revealed to be regulated by Fe deficiency [40, 48–55]. Evidence has shown that transcriptional expression of some Fe-responsive genes can be altered due to deficiencies or excesses of mineral elements, including cross-talk between Fe and other mineral elements. For example, the Fe transporter *LeIRT1* is reported to be up-regulated by potassium (K) deficiency, as revealed by microarray analysis [56], and expression of the K transporter gene *LeKC1* was induced not only by K starvation but also by Fe deficiency [56]. Via comprehensive analysis of Fe-responsive protein kinase (PK) and protein phosphatase (PP) genes, we found that strong over-representation of PK and PP genes that encode proteins is involved in K homeostasis, which supports the link between potassium uptake and Fe deficiency [44]. The ameliorative effect of K supply on Fe-deficient responses was previously reported [57]. Although several lines of evidence have suggested a link between Pi and Fe homeostasis [32–35, 37–39, 58], little genome-wide information on transcriptional expression changes in Fe-responsive genes under Pi deficiency is available, and the biological processes that these genes are involved in remain elusive in Arabidopsis.

By mining previous RNA-seq data sets, we present comprehensive information on transcriptional expression of overlapping genes regulated by Fe and Pi deficiency in Arabidopsis roots. In total, 579 overlapping genes, or less than 20 % of all differentially expressed genes evaluated in each treatment, were responsive to both Fe and Pi deficiency. Only 137 of the 579 genes had greater than twofold changes in transcript abundance (Additional file l; Table 1). Many of the most strongly induced genes under Fe deficiency, such as AT3G12900, *IRT1*, *FRO2*, *CYP82C4* and AT5G38820 [18, 41, 54], are among the 579 overlapping genes, while most of the strongest induced Pi deficiency-induced marker genes, such as pyridoxal phosphate phosphatase-related protein gene AT1G17710, transposable element gene AT2G04460, *ATISP1*, *SPX3*, *APT1* and *AT4*, are not overlapping [33, 35]. GO enrichment analysis of the 137 genes with changes greater than twofold (Additional file 4) showed that Fe response-related processes such as ‘cellular response to Fe ion’ and ‘cellular Fe ion homeostasis’ were enriched, but none of the Pi response-related processes were pronounced, suggesting that plant responses to Fe deficiency might be more specific than responses to Pi deficiency under the conditions presented herein. The most strongly induced Fe-responsive genes were clustered together and down-regulated under Pi deficiency, except for AT1G52120 in which transcript abundance was increased under both stress conditions (Fig. 2a). In this cluster, IRT1, FRO2, and BHLH039 are known to be involved in Fe acquisition and transcriptional regulation, and COPT2 was confirmed to participate in cross talk between Fe deficiency responses and low phosphate signaling in a recent study [59]. Other genes in the group (Fig. 2a), such as AT3G12900, CYP82C4, AT5G38820 and AT1G52120, do not have defined functions currently, but may be involved in responses to Fe deficiency or Pi deficiency or cross-talk between Fe deficiency responses and phosphate-deficient signaling. Another group of interesting genes are AT5G20790, *AtOCT1* and AT1G30560 (Fig. 2c) given that their transcriptional expression was among the most highly induced under Pi deficiency. In particular, both AT1G30560 and *AtOCT1* were significantly up-regulated upon Fe deficiency, suggesting that these two genes might play important roles in responses to both stresses. In animals, organic cation/carnitine transporters (OCTs) are associated with homeostasis and distribution of various small endogenous amines (e.g. carnitine, choline) and detoxification of xenobiotics like nicotine. AtOCT1 has been reported to be involved in Arabidopsis root development. Knockout of *AtOCT1* expression results in a higher degree of root branching compared to the wild type in vitro. This disordered development may be due to an inability to transport carnitine [60]. It has been well established that the number and length of lateral roots are increased under Pi deficiency in Arabidopsis and other plants. Therefore, whether AtOCT1-mediated transport of carnitine or related chemicals is involved in lateral root development under Pi deficiency remains elusive.

GO enrichment analysis of the 579 overlapping genes revealed that these Fe- and Pi-responsive genes were associated with diverse biological processes (Additional file 3), particularly with the GO categories ‘response to salt stress’, ‘response to oxidative stress’ and ‘response to zinc ion’ (Additional file 3). These results imply that acclimation of plants to Fe and Pi deficiency and possibly other nutritional stresses is associated with profound changes in the transcriptome, including stress-specific responses such as alteration of ribosome composition [43] and other general responses. Only four (AT1G27760, AT3G04720, AT4G11650 and AT5G24090) of the 21 genes associated with ‘response to salt stress’ had an increase in transcript abundance greater than 1.5-fold, suggesting that this common response to Fe and Pi deficiency might be less important than Fe response-related processes. GO enrichment analysis of the most responsive genes (i.e., those with greater than 1.5-fold change) revealed that Fe response-related processes, were enriched, but none of the Pi response-related processes were (Additional file 4),suggesting that Pi deficiency has more pronounced effects on Fe homeostasis than Fe deficiency has on Pi homeostasis.

Functional annotation of a given gene is the most important goal in modern molecular biology and is essential for understanding how the cell works. All omics studies are discovery tools and are not capable of defining gene function. The actual functions of differentially expressed genes under certain conditions discovered by high throughput platforms require further experimental evidence. However, current research platforms can discover hundreds to thousands of differentially expressed genes in a single run, and most of them are annotated as function unknown. Functional exploration of every differentially expressed gene without selection would be extremely laborious and impossible. Fortunately, co-expression analysis provides the option to choose genes of interest for further study. The basic idea of co-expression analysis is that genes that show transcriptionally coordinated expression patterns under diverse conditions are often functionally related [61], thus allowing functional predictions regarding genes with unknown functions inferred from their co-expression relationships with genes with known functions [62, 63]. Using co-expression analysis, we discovered ten, six and ten potentially critical regulatory modules with diverse nodes from inputs of the 579 (total overlapping genes), 137 (genes with changes greater than twofold) and 90 Fe deficiency-induced, Pi deficiency-repressed genes (Additional file 5; Figs. 4a, 5). Unexpectedly, only 50, 35 and 29 %, respectively, of the input genes were associated with formation of co-expression networks, suggesting that the majority of overlapping genes are functionally diverse and involved in a variety of biological processes. The network obtained from the group of 90 Fe deficiency-induced, Pi deficiency-repressed genes (Fig. 3a) is of particular interest. In this network, several genes may play important roles in responses to Fe and Pi deficiency. For instance, the gene AT1G74770 annotated with zinc ion binding protein showed a strong relationship with the Fe transporter *IRT1,* implying that this gene may be required for a Fe response. Another putative zinc ion binding protein encoding gene, AT1G18910, was shown to be connected to AT1G74770 and the transcription factor gene *WRKY17,* suggesting that these genes may also be involved in plant adaptation to Fe deficiency or zinc toxicity elicited by excess zinc under Fe deficiency.

It is generally accepted that a group of genes with similar expression patterns might be positively and/or negatively regulated by the same regulator(s). In Arabidopsis, the PHR1 transcription factor (TF) and its homolog PHL1 consist of the central regulatory system controlling transcriptional expression of a subset of Pi deficiency response genes by binding to the P1BS motif in promoter regions; while FIT and PYE are two major TFs regulating transcriptional expression of two subsets of Fe deficiency response genes. 35 out of the 579 genes, including Fe acquisition genes *IRT1* and *FRO2* and Fe responsive marker gene *CYP82C4*, were induced under Fe deficiency but down-regulated under Pi deficiency with changes greater than 1.5-fold. qPCR examination (Fig. 6a, b) confirmed that both *IRT1* and *FRO2* as well as *CYP82C4*, mainly regulated by FIT in response to Fe deficiency, were down-regulated by Pi deficiency in wild type plants, probably due to the decreased abundance of *FIT* (Fig. 6a). By contrast, the transcriptional expression of both *PYE* and its target AT1G74790 was not altered in response to Pi deficiency in both wild type and *phr1* mutant plants. Taken together, these results suggest that FIT-regulated but not PYE-regulated Fe-response genes are affected by Pi deficiency and the down-regulation by Pi deficiency might be partially due to the down-regulation of *FIT* (since although the transcript abundance of *FIT* was not significantly different between Pi-sufficient and -deficient conditions in *phr1* mutant plants, the transcriptional expression of *IRT1* and *FRO2* as well as *CYP82C4* was still significantly down-regulated by Pi deficiency). In addition, under Pi sufficiency, although the transcript level of *FIT* was not different between wild type and *phr1* mutant plants, the transcript abundance of *IRT1* and *FRO2* as well as *CYP82C4* was still significantly down-regulated in the mutant plants (Fig. 6a, b). These results indicate that, besides FIT and P1BS motif (*CYP82C4* doesn’t contain a P1BS motif in the −3000 bp sequence of its promoter region), some other factors may be involved in the down-regulation of gene expression under Pi deficiency. Indeed, only 34 % of the genes (12 of 35) contained at least one P1BS motif in promoter regions (−3000 bp sequence upstream of the translation start) and only 20 % (7 of 35) had a P1BS motif within −1000 bp of their promoter regions, further suggesting that other positive or negative regulators might be involved in down-regulation of these Pi-responsive genes. One of these regulators may be Fe itself. It has been reported that Pi deficiency results in enhanced Fe accessibility to plants in the media, which leads to an over accumulation of Fe in plants, subsequently causing down-regulated expression of Fe-responsive genes. This point of view was confirmed by supply of different Fe concentrations in the Pi deficient media (Fig. 6b). If no additional Fe was supplied to the Pi deficient media (−Fe−Pi), the transcriptional expression of all tested genes was induced both in wild type and *phr1* mutant plants, an expression pattern similar to the one of Fe deficiency (Fig. 6b). This result suggests that an extent of Fe in the Pi deficient media is required for the down-regulation of Fe-responsive genes under Pi deficiency. Indeed, Pi-deficiency caused down-regulation was much enhanced by supply of 5 µM Fe in the Pi deficient media both in wild type and *phr1* mutant plants (Fig. 6b). In the future, the dose effects of Fe in the Pi-deficient media on the transcriptional expression of Fe-responsive genes need further validation.

## Conclusions

In summary, we provide genome-wide information on the transcriptional expression of 579 overlapping genes that responded to both Fe and Pi deficiency in Arabidopsis roots and the biological processes that the genes are involved in. Gene clustering and root-specific co-expression analysis revealed several potentially important genes, including *CYP82C4* and AT5G38820, which likely function as putative novel players in response to Fe and Pi deficiency or in cross-talk between Fe-deficient responses and Pi-deficient signaling. These results imply that Pi deficiency has more pronounced effects on Fe homeostasis than Fe deficiency has on Pi homeostasis.

## Materials and methods

### Plant growth and treatments

Arabidopsis (*Arabidopsis thaliana*) seeds from the Columbia ecotype obtained from the Arabidopsis Biological Resource Center (ABRC) were used in this study. *Phr1* mutant seeds (SALK_067629C) were a gift from Professor Tzyy-Jen Chiou as previously described [64]. Seeds were surface sterilized by immersion in 5 % (v/v) NaOCl for 5 min and 70 % ethanol for 7 min, followed by four rinses in sterile water. Seeds were placed into Petri dishes and stored for 1 day at 4 °C in the dark. Plates were then transferred to a growth chamber and grown at 21 °C under continuous illumination (50 µmol m^−2^ s^−1^; Philips TL lamps). The agar-based medium [65] was composed of (mM): KNO_3_ (5), MgSO_4_ (2), Ca(NO_3_)_2_ (2), KH_2_PO_4_ (2.5); (µM): H_3_BO_3_ (70), MnCl_2_ (14), ZnSO_4_ (1), CuSO_4_ (0.5), NaCl (10), Na_2_MoO_4_ (0.2); and 40 µM Fe-EDTA solidified with 0.8 % agar (Sigma-Aldrich). Sucrose (43 mM) and 4.7 mM MES were included, and the pH was adjusted to 5.8. After 10 d of precultivation, plants were transferred either to fresh agar medium with 100 µM 3-(2-pyridyl)-5,6-diphenyl-1,2,4-triazine sulfonate without Fe, medium without Pi, medium without both Fe and Pi, medium without Pi containing 5 µm Fe or fresh control medium and grown for another 3 d. Lower potassium concentrations due to the absence of KH_2_PO_4_ in the Pi-free medium was corrected by addition of KCl.

### Quantitative reverse transcription-PCR

Total RNA was isolated using the RNeasy Plant Mini Kit (Qiagen) and treated with DNase using the TURBO DNA-free Kit (Ambion) as suggested by the manufacturer. cDNA was synthesized and qPCR was performed according to a previous report [40] using the SYBR Green PCR Master Mix (Applied Biosystems) with programs recommended by the manufacturer in the ABI Prism 7500 Sequence Detection System (Applied Biosystems). The melting temperature of the primers ranged from 58 to 62 °C. Primer pairs were selected using Primer3 (http://primer3.sourceforge.net/). Elongation factor1-β2 (At5g19510) and Tubulin3 (At5g19770) were used as internal controls (transcript abundance of both genes did not change under Fe and Pi deficiency) for transcript normalization. The primers used in this study are listed in Additional file 10. Three independent replicates were performed for each sample. The delta threshold cycle (∆ct = the ct of a gene−the ct of the reference) was used to determine the relative amount of gene expression. Student’s *t* test (P < 0.05) was used to compare differences between samples grown under treatment and control conditions.

### Data collection and processing

Transcriptomic data sets of roots from 13-day-old Arabidopsis seedlings grown in the presence or absence of Fe or Pi by RNA-seq were downloaded from a public database (NCBI: SRP044814, SRA050356.1). The 3106 and 3296 differentially expressed genes (P < 0.05) upon Pi and Fe deficiency were compared, and the resulting 579 overlapping genes were subsequently analyzed as shown in Fig. 1. Microarray data of 2671 ATH1 arrays from the NASCarray database (http://affymetrix.arabidopsis.info/) were downloaded and normalized using the RMA function in the Bioconductor Affy package software. Three hundred root-related arrays were manually identified as described [41] and were used as a database for co-expression analysis.

### Gene ontology analysis

The gene ontology browsing utility (GOBU) [66] was adopted for gene ontology (GO) enrichment analysis using the TopGo ‘elim’ method [67]. The elim algorithm iteratively removes the genes mapped to significant terms from higher level GO terms, thus avoiding enrichment of unimportant functional categories.

### Generation of co-expression networks using the MACCU toolbox

Gene functional networks were constructed based on 300 publicly available root-related microarrays using the MACCU toolbox [41], with a Pearson correlation threshold of 0.7. The generated co-expression networks were visualized by Cytoscape (http://www.cytoscape.org). If one cluster of genes did not have any connection (edges) to any other cluster in the co-expression network, it was referred to as a module.

## Additional files

10.1186/s13104-015-1524-y Subset of 579 overlapping genes between phosphate-deficiency regulated and iron-deficiency regulated in the Arabidopsis roots (P < 0.05). The fold change of the gene expression was indicated as mean with standard deviation (SD).
10.1186/s13104-015-1524-y Hierarchical cluster analysis of 579 overlapping genes with greater than twofold changes in transcript abundance in Arabidopsis roots grown under Fe- or Pi-deficient conditions.
10.1186/s13104-015-1524-y Gene Ontology enrichment was assessed using GOBU (Lin et al. [66]) in the 579 overlapping genes (elim, P < 0.01). In the term type column, P, F and C indicate biological process, functional process and subcellular localization, respectively.
10.1186/s13104-015-1524-y Representative Gene Ontology categories (in biological process) enriched in the 137 overlapping genes with twofold change in expression.
10.1186/s13104-015-1524-y Co-expression relationships of the 579 differentially expressed overlapping genes.
10.1186/s13104-015-1524-y Subset of 210 overlapping genes consists of the largest co-expression module. The fold change of the gene expression was indicated as mean with standard deviation (SD).
10.1186/s13104-015-1524-y Gene ontology enrichment was assessed using GOBU (Lin et al. [66]) in the 210 overlapping genes comprising the largest model in Figure S2 (elim, P < 0.01). In the term type column, P, F and C indicate biological process, functional process and subcellular localization, respectively.
10.1186/s13104-015-1524-y Co-expression relationships of the 97 differentially expressed overlapping genes induced by Pi starvation but repressed by Fe deficiency.
10.1186/s13104-015-1524-y Subset of 35 overlapping genes induced by iron deficiency with fold change more than 1.5-fold but down-regulated by Pi starvation the Arabidopsis roots (P < 0.05). The fold change of the gene expression was indicated as mean with standard deviation (SD).
10.1186/s13104-015-1524-y Primers used in this study.
