# Supplementary material for: Genome-wide analysis of overlapping genes regulated by iron deficiency and phosphate starvation reveals new interactions in Arabidopsis roots
Source: BMC Res Notes. 2015 Oct 12;8:555. doi: 10.1186/s13104-015-1524-y (PMC4604098; doi:10.1186/s13104-015-1524-y)
Supplement: Supplementary file 1 — 10.1186/s13104-015-1524-y Subset of 579 overlapping genes between phosphate-deficiency regulated and iron-deficiency regulated in the Arabidopsis roots (P < 0.05). The fold change of the gene expression was indicated as mean with standard deviation (SD). [file 13104_2015_1524_MOESM1_ESM.doc]

**Additional file 1** Subset of 579 overlapping genes between phosphate-deficiency regulated and iron-deficiency regulated in the Arabidopsis roots (P<0.05). The fold change of the gene expression was indicated as mean with standard deviation (SD).

| 列**AGI** | 列2 **Annotation** | **Mean(Fe-/Fe+)** | SD | **Mean(Pi-/Pi+)** | **SD** |  |
| --- | --- | --- | --- | --- | --- | --- |
| At1G01070 | Nodulin MtN21 /EamA-like transporter family protein | 0.74 | 0.05 | 1.27 | 0.03 |  |
| At1G01090 | PDH-E1 ALPHA, pyruvate dehydrogenase E1 alpha | 0.84 | 0.01 | 0.88 | 0.03 |  |
| At1G01380 | ETC1, Homeodomain-like superfamily protein | 3.22 | 1.09 | 3.73 | 1.22 |  |
| At1G01430 | TBL25, TRICHOME BIREFRINGENCE-LIKE 25 | 0.86 | 0.05 | 0.86 | 0.04 |  |
| At1G01580 | ATFRO2, FRD1, FRO2, ferric reduction oxidase 2 | 59.73 | 10.56 | 0.35 | 0.05 |  |
| At1G01630 | Sec14p-like phosphatidylinositol transfer family protein | 1.45 | 0.20 | 1.17 | 0.12 |  |
| At1G01660 | RING/U-box superfamily protein | 1.49 | 0.11 | 1.58 | 0.20 |  |
| At1G01960 | EDA10, SEC7-like guanine nucleotide exchange family protein | 0.91 | 0.02 | 0.78 | 0.07 |  |
| At1G03080 | Kinase interacting (KIP1-like) family protein | 1.37 | 0.03 | 0.86 | 0.09 |  |
| At1G04290 | Thioesterase superfamily protein | 0.88 | 0.02 | 1.49 | 0.21 |  |
| At1G04680 | Pectin lyase-like superfamily protein | 0.81 | 0.04 | 0.80 | 0.08 |  |
| At1G04850 | Ubiquitin-associated (UBA)/TS-N domain-containing protein | 1.11 | 0.01 | 1.05 | 0.03 |  |
| At1G05300 | ZIP5, zinc transporter 5 precursor | 0.49 | 0.05 | 2.13 | 0.11 |  |
| At1G07590 | Tetratricopeptide repeat (TPR)-like superfamily protein | 1.70 | 0.18 | 1.24 | 0.10 |  |
| At1G08160 | Late embryogenesis abundant (LEA) hydroxyproline-rich glycoprotein family | 0.80 | 0.15 | 0.82 | 0.01 |  |
| At1G08190 | ATVAM2, ATVPS41, VAM2, VPS41, ZIP2, vacuolar protein sorting 41 | 1.07 | 0.02 | 0.89 | 0.02 |  |
| At1G08560 | ATSYP111, KN, SYP111, syntaxin of plants 111 | 0.87 | 0.09 | 0.87 | 0.04 |  |
| At1G08650 | ATPPCK1, PPCK1, phosphoenolpyruvate carboxylase kinase 1 | 1.77 | 0.16 | 2.58 | 0.21 |  |
| At1G08840 | Emb2411, DNA replication helicase, putative | 0.91 | 0.06 | 0.77 | 0.02 |  |
| At1G09430 | ACLA-3, ATP-citrate lyase A-3 | 1.29 | 0.06 | 0.88 | 0.03 |  |
| At1G09620 | ATP binding;leucine-tRNA ligases;aminoacyl-tRNA ligases;nucleotide binding;ATP binding;aminoacyl-tRNA ligases | 0.87 | 0.05 | 0.74 | 0.10 |  |
| At1G09780 | Phosphoglycerate mutase, 2,3-bisphosphoglycerate-independent | 1.33 | 0.03 | 1.18 | 0.04 |  |
| At1G10780 | F-box/RNI-like superfamily protein | 0.82 | 0.05 | 0.87 | 0.03 |  |
| At1G10970 | ATZIP4, ZIP4, zinc transporter 4 precursor | 0.62 | 0.14 | 1.65 | 0.26 |  |
| At1G11080 | Scpl31, serine carboxypeptidase-like 31 | 0.63 | 0.08 | 0.42 | 0.08 |  |
| At1G11920 | Pectin lyase-like superfamily protein | #DIV/0! | ###### | 2.68 | 0.51 |  |
| At1G12090 | ELP, extensin-like protein | 0.89 | 0.08 | 1.14 | 0.05 |  |
| At1G12520 | ATCCS, CCS, copper chaperone for SOD1 | 1.31 | 0.03 | 0.88 | 0.06 |  |
| At1G12800 | Nucleic acid-binding, OB-fold-like protein | 0.87 | 0.08 | 0.79 | 0.09 |  |
| At1G12810 | Proline-rich family protein | 1.17 | 0.08 | 1.16 | 0.10 |  |
| At1G13300 | HRS1, myb-like transcription factor family protein | 1.19 | 0.08 | 1.33 | 0.15 |  |
| At1G13320 | PP2AA3, protein phosphatase 2A subunit A3 | 0.91 | 0.04 | 0.93 | 0.05 |  |
| At1G14190 | Glucose-methanol-choline (GMC) oxidoreductase family protein | 2.29 | 0.41 | 0.78 | 0.08 |  |
| At1G14220 | Ribonuclease T2 family protein | 0.74 | 0.18 | 2.89 | 0.27 |  |
| At1G14870 | PCR2, PLANT CADMIUM RESISTANCE 2 | 1.47 | 0.20 | 1.66 | 0.17 |  |
| At1G15040 | Class I glutamine amidotransferase-like superfamily protein | 0.60 | 0.04 | 3.73 | 1.12 |  |
| At1G15165 | RING/FYVE/PHD zinc finger superfamily protein | 0.82 | 0.04 | 0.79 | 0.04 |  |
| At1G15210 | ATPDR7, PDR7, pleiotropic drug resistance 7 | 0.87 | 0.00 | 0.77 | 0.06 |  |
| At1G15290 | Tetratricopeptide repeat (TPR)-like superfamily protein | 0.87 | 0.03 | 0.75 | 0.12 |  |
| At1G15380 | Lactoylglutathione lyase / glyoxalase I family protein | 0.55 | 0.04 | 0.43 | 0.09 |  |
| At1G16430 | Surfeit locus protein 5 subunit 22 of Mediator complex | 0.79 | 0.10 | 1.27 | 0.12 |  |
| At1G17220 | FUG1, Translation initiation factor 2, small GTP-binding protein | 0.72 | 0.01 | 0.71 | 0.10 |  |
| At1G18360 | Alpha/beta-Hydrolases superfamily protein | 0.70 | 0.12 | 1.17 | 0.04 |  |
| At1G18910 | Zinc ion binding;zinc ion binding | 3.33 | 0.13 | 0.78 | 0.04 |  |
| At1G18970 | GLP4, germin-like protein 4 | 0.71 | 0.12 | 3.04 | 0.41 |  |
| At1G19570 | ATDHAR1, DHAR1, DHAR5, dehydroascorbate reductase | 0.86 | 0.06 | 1.20 | 0.07 |  |
| At1G20390 | Transposable element gene | 0.56 | 0.11 | 2.07 | 0.04 |  |
| At1G21810 | Plant protein of unknown function (DUF869) | 0.84 | 0.07 | 0.77 | 0.03 |  |
| At1G22070 | TGA3, TGA1A-related gene 3 | 1.20 | 0.04 | 1.55 | 0.09 |  |
| At1G22410 | Class-II DAHP synthetase family protein | 1.55 | 0.13 | 1.32 | 0.07 |  |
| At1G22430 | GroES-like zinc-binding dehydrogenase family protein | 0.58 | 0.15 | 0.75 | 0.03 |  |
| At1G22500 | RING/U-box superfamily protein | 0.78 | 0.10 | 0.70 | 0.05 |  |
| At1G22540 | Major facilitator superfamily protein | 1.40 | 0.11 | 0.85 | 0.06 |  |
| At1G23140 | Calcium-dependent lipid-binding (CaLB domain) family protein | 1.58 | 0.43 | 5.33 | 1.19 |  |
| At1G23310 | AOAT1, GGAT1, GGT1, glutamate:glyoxylate aminotransferase | 0.69 | 0.01 | 0.76 | 0.09 |  |
| At1G24150 | ATFH4, FH4, formin homologue 4 | 1.16 | 0.06 | 1.19 | 0.06 |  |
| At1G24320 | Six-hairpin glycosidases superfamily protein | 2.26 | 0.27 | 0.78 | 0.09 |  |
| At1G24560 | Unknown protein | 1.21 | 0.09 | 0.88 | 0.05 |  |
| At1G26250 | Proline-rich extensin-like family protein | 0.55 | 0.16 | 2.00 | 0.82 |  |
| At1G26300 | BSD domain-containing protein | 0.83 | 0.04 | 1.08 | 0.04 |  |
| At1G27030 | Unknown protein | 1.40 | 0.09 | 1.34 | 0.07 |  |
| At1G27760 | ATSAT32, SAT32, interferon-related developmental regulator family protein / IFRD protein family | 1.19 | 0.03 | 1.76 | 0.20 |  |
| At1G29280 | ATWRKY65, WRKY65, WRKY DNA-binding protein 65 | 1.51 | 0.05 | 1.23 | 0.12 |  |
| At1G30130 | Unknown protein | 0.77 | 0.05 | 0.79 | 0.07 |  |
| At1G30270 | ATCIPK23, CIPK23, LKS1, SnRK3.23, CBL-interacting protein kinase 23 | 0.85 | 0.10 | 0.87 | 0.06 |  |
| At1G30360 | ERD4, Early-responsive to dehydration stress protein (ERD4) | 0.88 | 0.04 | 0.78 | 0.08 |  |
| At1G30560 | Major facilitator superfamily protein | #DIV/0! | ###### | 55.93 | 32.45 |  |
| At1G30610 | EMB2279, EMB88, pentatricopeptide (PPR) repeat-containing protein | 0.88 | 0.04 | 0.80 | 0.04 |  |
| At1G30850 | RSH4, root hair specific 4 | 1.38 | 0.14 | 1.52 | 0.21 |  |
| At1G33250 | Protein of unknown function (DUF604) | 1.18 | 0.03 | 0.85 | 0.08 |  |
| At1G33780 | Protein of unknown function (DUF179) | 1.14 | 0.09 | 1.70 | 0.10 |  |
| At1G35580 | CINV1, cytosolic invertase 1 | 1.33 | 0.10 | 0.72 | 0.03 |  |
| At1G36060 | Integrase-type DNA-binding superfamily protein | 0.57 | 0.13 | 1.36 | 0.13 |  |
| At1G36160 | ACC1, AT-ACC1, EMB22, GK, PAS3, acetyl-CoA carboxylase 1 | 0.80 | 0.01 | 0.82 | 0.09 |  |
| At1G36180 | ACC2, acetyl-CoA carboxylase 2 | 1.25 | 0.03 | 0.77 | 0.06 |  |
| At1G43650 | Nodulin MtN21 /EamA-like transporter family protein | 0.66 | 0.05 | 0.71 | 0.06 |  |
| At1G43710 | Emb1075, Pyridoxal phosphate (PLP)-dependent transferases superfamily protein | 0.84 | 0.03 | 0.76 | 0.05 |  |
| At1G44750 | ATPUP11, PUP11, purine permease 11 | 1.12 | 0.05 | 1.08 | 0.00 |  |
| At1G45145 | ATH5, ATTRX5, LIV1, TRX5, thioredoxin H-type 5 | 1.95 | 0.27 | 1.59 | 0.26 |  |
| At1G47565 | Transposable element gene | 1.60 | 0.29 | 0.80 | 0.07 |  |
| At1G48300 | Unknown protein | 2.27 | 0.25 | 0.85 | 0.07 |  |
| At1G48410 | AGO1, Stabilizer of iron transporter SufD / Polynucleotidyl transferase | 0.85 | 0.09 | 0.79 | 0.08 |  |
| At1G48790 | AMSH1, associated molecule with the SH3 domain of STAM 1 | 1.16 | 0.02 | 0.93 | 0.05 |  |
| At1G49450 | Transducin/WD40 repeat-like superfamily protein | 0.74 | 0.06 | 0.58 | 0.13 |  |
| At1G50060 | CAP (Cysteine-rich secretory proteins, Antigen 5, and Pathogenesis-related 1 protein) superfamily protein | 0.71 | 0.04 | 1.42 | 0.05 |  |
| At1G50110 | D-aminoacid aminotransferase-like PLP-dependent enzymes superfamily protein | 0.55 | 0.02 | 1.62 | 0.09 |  |
| At1G50250 | FTSH1, FTSH protease 1 | 0.85 | 0.01 | 0.81 | 0.05 |  |
| At1G51070 | bHLH115, basic helix-loop-helix (bHLH) DNA-binding superfamily protein | 1.26 | 0.02 | 1.11 | 0.04 |  |
| At1G51420 | ATSPP1, SPP1, sucrose-phosphatase 1 | 1.24 | 0.12 | 1.40 | 0.24 |  |
| At1G51680 | 4CL.1, 4CL1, AT4CL1, 4-coumarate:CoA ligase 1 | 3.22 | 0.06 | 1.27 | 0.19 |  |
| At1G51860 | Leucine-rich repeat protein kinase family protein | 1.97 | 0.32 | 1.20 | 0.05 |  |
| At1G51870 | Protein kinase family protein | 2.42 | 0.42 | 2.25 | 0.83 |  |
| At1G52050 | Mannose-binding lectin superfamily protein | 0.70 | 0.04 | 1.32 | 0.11 |  |
| At1G52120 | Mannose-binding lectin superfamily protein | 157.65 | 59.83 | 6.13 | 2.70 |  |
| At1G52410 | TSA1, TSK-associating protein 1 | 0.91 | 0.02 | 1.17 | 0.03 |  |
| At1G53140 | DRP5A, Dynamin related protein 5A | 0.87 | 0.04 | 0.74 | 0.03 |  |
| At1G53310 | ATPEPC1, ATPPC1, PEPC1, PPC1, phosphoenolpyruvate carboxylase 1 | 2.34 | 0.19 | 2.21 | 0.25 |  |
| At1G53830 | ATPME2, PME2, pectin methylesterase 2 | 0.79 | 0.12 | 0.74 | 0.08 |  |
| At1G54575 | Unknown protein | 1.63 | 0.31 | 1.89 | 0.25 |  |
| At1G56145 | Leucine-rich repeat transmembrane protein kinase | 1.21 | 0.05 | 0.85 | 0.03 |  |
| At1G56680 | Chitinase family protein | 0.68 | 0.04 | 1.36 | 0.10 |  |
| At1G58080 | ATATP-PRT1, ATP-PRT1, HISN1A, ATP phosphoribosyl transferase 1 | 1.69 | 0.09 | 1.16 | 0.07 |  |
| At1G62280 | SLAH1, SLAC1 homologue 1 | 0.40 | 0.06 | 0.04 | 0.04 |  |
| At1G62422 | Unknown protein | 2.25 | 0.43 | 1.37 | 0.10 |  |
| At1G62750 | ATSCO1, ATSCO1/CPEF-G, SCO1, Translation elongation factor EFG/EF2 protein | 0.77 | 0.04 | 0.67 | 0.09 |  |
| At1G62970 | Chaperone DnaJ-domain superfamily protein | 0.81 | 0.02 | 0.81 | 0.05 |  |
| At1G63010 | Major Facilitator Superfamily with SPX (SYG1/Pho81/XPR1) domain-containing protein | 0.85 | 0.03 | 1.27 | 0.10 |  |
| At1G64590 | NAD(P)-binding Rossmann-fold superfamily protein | 2.06 | 0.13 | 2.39 | 0.70 |  |
| At1G65690 | Late embryogenesis abundant (LEA) hydroxyproline-rich glycoprotein family | 1.75 | 0.18 | 1.47 | 0.22 |  |
| At1G65840 | ATPAO4, PAO4, polyamine oxidase 4 | 1.74 | 0.07 | 1.79 | 0.34 |  |
| At1G65900 | Unknown protein | 0.71 | 0.14 | 0.77 | 0.04 |  |
| At1G65930 | cICDH, cytosolic NADP+-dependent isocitrate dehydrogenase | 0.94 | 0.01 | 0.93 | 0.01 |  |
| At1G66050 | ORTH5, VIM2, Zinc finger (C3HC4-type RING finger) family protein | 0.83 | 0.11 | 0.86 | 0.11 |  |
| At1G67800 | Copine (Calcium-dependent phospholipid-binding protein) family | 1.54 | 0.19 | 1.51 | 0.17 |  |
| At1G67940 | ATNAP3, AtSTAR1, NAP3, NAP3, non-intrinsic ABC protein 3 | 1.38 | 0.10 | 1.33 | 0.17 |  |
| At1G68430 | Unknown protein | 1.39 | 0.25 | 1.35 | 0.11 |  |
| At1G68740 | PHO1;H1, EXS (ERD1/XPR1/SYG1) family protein | 0.39 | 0.08 | 2.31 | 0.44 |  |
| At1G68890 | Magnesium ion binding;thiamin pyrophosphate binding;hydro-lyases;catalytics;2-succinyl-5-enolpyruvyl-6-hydroxy-3-cyclohexene-1-carboxylic-acid synthases | 0.84 | 0.07 | 0.82 | 0.09 |  |
| At1G69220 | SIK1, Protein kinase superfamily protein | 0.93 | 0.02 | 0.90 | 0.04 |  |
| At1G69240 | ATMES15, MES15, RHS9, methyl esterase 15 | 1.17 | 0.04 | 1.64 | 0.16 |  |
| At1G69420 | DHHC-type zinc finger family protein | 0.92 | 0.00 | 0.88 | 0.02 |  |
| At1G70200 | RNA-binding (RRM/RBD/RNP motifs) family protein | 0.84 | 0.05 | 0.71 | 0.04 |  |
| At1G70300 | KUP6, K+ uptake permease 6 | 1.32 | 0.17 | 1.32 | 0.08 |  |
| At1G71720 | Nucleic acid-binding proteins superfamily | 0.68 | 0.09 | 0.78 | 0.03 |  |
| At1G71870 | MATE efflux family protein | 0.67 | 0.04 | 0.49 | 0.06 |  |
| At1G72070 | Chaperone DnaJ-domain superfamily protein | 1.72 | 0.62 | 6.60 | 3.34 |  |
| At1G72150 | PATL1, PATELLIN 1 | 0.87 | 0.04 | 0.79 | 0.03 |  |
| At1G72160 | Sec14p-like phosphatidylinositol transfer family protein | 0.83 | 0.02 | 0.90 | 0.03 | |
| At1G72300 | Leucine-rich receptor-like protein kinase family protein | 1.26 | 0.03 | 0.77 | 0.03 | |
| At1G72900 | Toll-Interleukin-Resistance (TIR) domain-containing protein | 0.49 | 0.06 | 1.88 | 0.29 | |
| At1G73120 | Unknown protein | 8.98 | 2.09 | 0.28 | 0.11 | |
| At1G73170 | P-loop containing nucleoside triphosphate hydrolases superfamily protein | 0.81 | 0.06 | 1.36 | 0.05 | |
| At1G73220 | 1-Oct, AtOCT1, organic cation/carnitine transporter1 | 7.38 | 6.18 | 196.69 | 139.63 | |
| At1G73600 | S-adenosyl-L-methionine-dependent methyltransferases superfamily protein | 0.61 | 0.06 | 0.23 | 0.02 |  |
| At1G73660 | Protein tyrosine kinase family protein | 0.90 | 0.06 | 0.71 | 0.05 |  |
| At1G73820 | Ssu72-like family protein | 0.91 | 0.03 | 1.16 | 0.06 |  |
| At1G73920 | Alpha/beta-Hydrolases superfamily protein | 0.87 | 0.02 | 0.61 | 0.02 |  |
| At1G74020 | SS2, strictosidine synthase 2 | 0.84 | 0.03 | 1.23 | 0.07 |  |
| At1G74770 | Zinc ion binding | 6.43 | 0.50 | 0.66 | 0.07 |  |
| At1G74790 | Catalytics | 1.35 | 0.11 | 1.10 | 0.03 |  |
| At1G75270 | DHAR2, dehydroascorbate reductase 2 | 1.14 | 0.02 | 1.38 | 0.09 |  |
| At1G75820 | ATCLV1, CLV1, FAS3, FLO5, Leucine-rich receptor-like protein kinase family protein | 0.78 | 0.06 | 0.72 | 0.12 |  |
| At1G76950 | PRAF1, Regulator of chromosome condensation (RCC1) family with FYVE zinc finger domain | 1.58 | 0.04 | 0.89 | 0.05 |  |
| At1G77760 | GNR1, NIA1, NR1, nitrate reductase 1 | 0.58 | 0.03 | 0.54 | 0.02 |  |
| At1G77920 | bZIP transcription factor family protein | 1.50 | 0.15 | 1.48 | 0.23 |  |
| At1G78150 | Unknown protein | 0.93 | 0.05 | 1.30 | 0.00 |  |
| At1G78230 | Outer arm dynein light chain 1 protein | 2.51 | 0.57 | 0.59 | 0.22 |  |
| At1G78300 | 14-3-3OMEGA, GF14 OMEGA, GRF2, general regulatory factor 2 | 0.86 | 0.05 | 0.92 | 0.06 |  |
| At1G78820 | D-mannose binding lectin protein with Apple-like carbohydrate-binding domain | 0.57 | 0.18 | 0.52 | 0.13 |  |
| At1G79450 | ALIS5, ALA-interacting subunit 5 | 1.55 | 0.21 | 1.24 | 0.10 |  |
| At1G80030 | Molecular chaperone Hsp40/DnaJ family protein | 0.87 | 0.02 | 0.82 | 0.06 |  |
| At2G01670 | Atnudt17, NUDT17, nudix hydrolase homolog 17 | 1.88 | 0.04 | 1.48 | 0.10 |  |
| At2G01820 | Leucine-rich repeat protein kinase family protein | 0.82 | 0.05 | 0.75 | 0.04 |  |
| At2G01880 | ATPAP7, PAP7, purple acid phosphatase 7 | 3.14 | 0.21 | 3.31 | 0.81 |  |
| At2G02310 | AtPP2-B6, PP2-B6, phloem protein 2-B6 | 5.30 | 2.01 | 0.49 | 0.12 |  |
| At2G02390 | ATGSTZ1, GST18, GSTZ1, glutathione S-transferase zeta 1 | 1.16 | 0.04 | 1.21 | 0.09 |  |
| At2G02450 | Anac034, ANAC035, LOV1, NAC035, NAC domain containing protein 35 | 0.30 | 0.23 | 0.41 | 0.24 |  |
| At2G04235 | Unknown protein | 0.83 | 0.06 | 0.72 | 0.09 |  |
| At2G05920 | Subtilase family protein | 0.87 | 0.01 | 0.74 | 0.07 |  |
| At2G07050 | CAS1, cycloartenol synthase 1 | 0.89 | 0.02 | 0.86 | 0.04 |  |
| At2G14210 | AGL44, ANR1, AGAMOUS-like 44 | 2.01 | 0.37 | 0.82 | 0.09 |  |
| At2G15620 | ATHNIR, NIR, NIR1, nitrite reductase 1 | 0.63 | 0.01 | 0.67 | 0.06 |  |
| At2G16430 | ATPAP10, PAP10, purple acid phosphatase 10 | 0.68 | 0.08 | 2.26 | 0.33 |  |
| At2G17050 | Disease resistance protein (TIR-NBS-LRR class), putative | 0.60 | 0.05 | 1.19 | 0.12 |  |
| At2G17280 | Phosphoglycerate mutase family protein | 1.16 | 0.02 | 2.01 | 0.47 |  |
| At2G18193 | P-loop containing nucleoside triphosphate hydrolases superfamily protein | 2.07 | 0.26 | 1.29 | 0.01 |  |
| At2G18730 | ATDGK3, DGK3, diacylglycerol kinase 3 | 1.34 | 0.01 | 1.45 | 0.03 |  |
| At2G19090 | Protein of unknown function (DUF630 and DUF632) | 0.66 | 0.17 | 0.79 | 0.04 |  |
| At2G20050 | Protein serine/threonine phosphatases;protein kinases;catalytics;cAMP-dependent protein kinase regulators;ATP binding;protein serine/threonine phosphatases | 1.10 | 0.02 | 0.77 | 0.12 |  |
| At2G20990 | ATSYTA, NTMC2T1.1, NTMC2TYPE1.1, SYT1, SYTA, synaptotagmin A | 0.90 | 0.03 | 0.87 | 0.02 |  |
| At2G21050 | LAX2, like AUXIN RESISTANT 2 | 0.86 | 0.10 | 0.79 | 0.05 |  |
| At2G21540 | ATSFH3, SFH3, SEC14-like 3 | 1.89 | 0.09 | 0.71 | 0.13 |  |
| At2G22080 | Unknown protein | 1.12 | 0.08 | 1.93 | 0.17 |  |
| At2G22122 | Unknown protein | 0.41 | 0.09 | 0.31 | 0.06 |  |
| At2G22125 | CSI1, binding | 0.88 | 0.05 | 0.81 | 0.10 |  |
| At2G22200 | Integrase-type DNA-binding superfamily protein | 0.47 | 0.30 | 0.44 | 0.10 |  |
| At2G22290 | ATRAB-H1D, ATRAB6, ATRABH1D, RAB-H1D, RABH1d, RAB GTPase homolog H1D | 1.91 | 0.66 | 2.10 | 0.59 |  |
| At2G23960 | Class I glutamine amidotransferase-like superfamily protein | 1.55 | 0.27 | 2.13 | 0.21 |  |
| At2G24570 | ATWRKY17, WRKY17, WRKY DNA-binding protein 17 | 1.15 | 0.05 | 0.85 | 0.02 |  |
| At2G25180 | ARR12, RR12, response regulator 12 | 1.20 | 0.08 | 0.92 | 0.05 |  |
| At2G25240 | Serine protease inhibitor (SERPIN) family protein | 1.53 | 0.15 | 2.27 | 0.08 |  |
| At2G25730 | Unknown protein | 1.11 | 0.05 | 0.79 | 0.05 |  |
| At2G26760 | CYCB1;4, Cyclin B1;4 | 0.84 | 0.03 | 0.85 | 0.05 |  |
| At2G26780 | ARM repeat superfamily protein | 1.12 | 0.04 | 0.82 | 0.06 |  |
| At2G26980 | CIPK3, SnRK3.17, CBL-interacting protein kinase 3 | 0.76 | 0.05 | 0.75 | 0.06 |  |
| At2G27170 | SMC3, TTN7, Structural maintenance of chromosomes (SMC) family protein | 1.11 | 0.01 | 0.78 | 0.06 |  |
| At2G28190 | CSD2, CZSOD2, copper/zinc superoxide dismutase 2 | 1.12 | 0.03 | 0.84 | 0.06 |  |
| At2G28470 | BGAL8, beta-galactosidase 8 | 0.79 | 0.08 | 0.80 | 0.09 |  |
| At2G28780 | Unknown protein | 0.72 | 0.03 | 0.36 | 0.10 |  |
| At2G28840 | XBAT31, XB3 ortholog 1 in Arabidopsis thaliana | 0.85 | 0.02 | 0.88 | 0.04 |  |
| At2G29000 | Leucine-rich repeat protein kinase family protein | 1.85 | 0.60 | 3.03 | 0.79 |  |
| At2G29620 | Unknown protein | 1.47 | 0.13 | 1.34 | 0.26 |  |
| At2G30520 | RPT2, Phototropic-responsive NPH3 family protein | 0.87 | 0.02 | 0.79 | 0.08 |  |
| At2G30840 | 2-Oxoglutarate (2OG) and Fe(II)-dependent oxygenase superfamily protein | 1.53 | 0.15 | 1.24 | 0.06 |  |
| At2G32230 | PRORP1, proteinaceous RNase P 1 | 0.79 | 0.08 | 0.87 | 0.02 |  |
| At2G32590 | CONTAINS InterPro DOMAIN/s: Barren (InterPro:IPR008418) | 0.75 | 0.06 | 0.79 | 0.05 |  |
| At2G32960 | Phosphotyrosine protein phosphatases superfamily protein | 2.07 | 0.23 | 4.79 | 1.23 |  |
| At2G33020 | AtRLP24, RLP24, receptor like protein 24 | 5.05 | 1.74 | 0.48 | 0.33 |  |
| At2G33450 | Ribosomal L28 family | 0.73 | 0.09 | 0.86 | 0.07 |  |
| At2G33570 | Domain of unknown function (DUF23) | 0.90 | 0.03 | 0.87 | 0.03 |  |
| At2G34610 | Unknown protein | 0.42 | 0.07 | 0.60 | 0.06 |  |
| At2G35780 | Scpl26, serine carboxypeptidase-like 26 | 0.78 | 0.08 | 0.90 | 0.03 |  |
| At2G36390 | BE3, SBE2.1, starch branching enzyme 2.1 | 0.82 | 0.01 | 1.46 | 0.05 |  |
| At2G36530 | ENO2, LOS2, Enolase | 1.29 | 0.10 | 1.26 | 0.05 |  |
| At2G37050 | Leucine-rich repeat protein kinase family protein | 0.92 | 0.05 | 0.83 | 0.09 |  |
| At2G37330 | ALS3, aluminum sensitive 3 | 0.66 | 0.09 | 1.41 | 0.16 |  |
| At2G38300 | Myb-like HTH transcriptional regulator family protein | 1.37 | 0.26 | 1.26 | 0.17 |  |
| At2G38330 | MATE efflux family protein | 0.73 | 0.10 | 0.76 | 0.07 |  |
| At2G38460 | ATIREG1, FPN1, IREG1, iron regulated 1 | 0.52 | 0.13 | 0.62 | 0.04 |  |
| At2G38870 | Serine protease inhibitor, potato inhibitor I-type family protein | 0.71 | 0.12 | 1.64 | 0.28 |  |
| At2G39190 | ATATH8, Protein kinase superfamily protein | 0.87 | 0.07 | 0.82 | 0.07 |  |
| At2G39800 | ATP5CS, P5CS1, delta1-pyrroline-5-carboxylate synthase 1 | 0.88 | 0.02 | 1.37 | 0.06 |  |
| At2G41040 | S-adenosyl-L-methionine-dependent methyltransferases superfamily protein | 1.20 | 0.03 | 0.91 | 0.04 |  |
| At2G41560 | ACA4, autoinhibited Ca(2+)-ATPase, isoform 4 | 0.79 | 0.08 | 0.67 | 0.05 |  |
| At2G42365 | Other RNA | 0.65 | 0.23 | 0.84 | 0.04 |  |
| At2G42600 | ATPPC2, PPC2, phosphoenolpyruvate carboxylase 2 | 0.77 | 0.05 | 2.18 | 0.09 |  |
| At2G43200 | S-adenosyl-L-methionine-dependent methyltransferases superfamily protein | 0.85 | 0.08 | 0.73 | 0.04 |  |
| At2G43410 | FPA, RNA binding | 0.89 | 0.03 | 0.84 | 0.02 |  |
| At2G43570 | CHI, chitinase, putative | 2.22 | 0.87 | 2.26 | 0.45 |  |
| At2G43910 | ATHOL1, HOL1, HARMLESS TO OZONE LAYER 1 | 0.81 | 0.05 | 1.20 | 0.04 |  |
| At2G45910 | U-box domain-containing protein kinase family protein | 0.91 | 0.03 | 1.93 | 0.17 |  |
| At2G46170 | Reticulon family protein | 1.12 | 0.10 | 1.18 | 0.06 |  |
| At2G46890 | Protein of unknown function (DUF1295) | 0.88 | 0.07 | 0.86 | 0.02 |  |
| At2G47160 | BOR1, HCO3- transporter family | 0.55 | 0.01 | 0.77 | 0.05 |  |
| At2G47260 | ATWRKY23, WRKY23, WRKY DNA-binding protein 23 | 1.46 | 0.04 | 0.67 | 0.05 |  |
| At2G47500 | P-loop nucleoside triphosphate hydrolases superfamily protein with CH (Calponin Homology) domain | 0.85 | 0.03 | 0.74 | 0.06 |  |
| At3G01260 | Galactose mutarotase-like superfamily protein | 0.76 | 0.09 | 0.44 | 0.03 |  |
| At3G01680 | CONTAINS InterPro DOMAIN/s: Mediator complex subunit Med28 (InterPro:IPR021640) | 0.92 | 0.06 | 0.79 | 0.11 |  |
| At3G02730 | ATF1, TRXF1, thioredoxin F-type 1 | 0.72 | 0.06 | 1.16 | 0.08 |  |
| At3G02850 | SKOR, STELAR K+ outward rectifier | 0.39 | 0.03 | 0.15 | 0.01 |  |
| At3G02870 | VTC4, Inositol monophosphatase family protein | 0.79 | 0.06 | 4.64 | 0.29 |  |
| At3G03040 | F-box/RNI-like superfamily protein | 0.75 | 0.04 | 0.79 | 0.04 |  |
| At3G03160 | FUNCTIONS IN: molecular_function unknown; INVOLVED IN: intracellular protein transport | 0.83 | 0.05 | 1.21 | 0.12 |  |
| At3G03190 | ATGSTF11, ATGSTF6, GSTF11, glutathione S-transferase F11 | 1.44 | 0.11 | 1.93 | 0.15 |  |
| At3G03250 | AtUGP1, UGP, UGP1, UDP-GLUCOSE PYROPHOSPHORYLASE 1 | 0.90 | 0.05 | 1.19 | 0.04 |  |
| At3G04320 | Kunitz family trypsin and protease inhibitor protein | 0.74 | 0.05 | 1.91 | 0.14 |  |
| At3G04330 | Kunitz family trypsin and protease inhibitor protein | 0.70 | 0.03 | 1.94 | 0.43 |  |
| At3G04720 | HEL, PR-4, PR4, pathogenesis-related 4 | 0.62 | 0.09 | 1.76 | 0.33 |  |
| At3G05810 | FUNCTIONS IN: molecular_function unknown; INVOLVED IN: biological_process unknown | 0.82 | 0.08 | 1.27 | 0.15 |  |
| At3G05858 | Unknown protein | 1.64 | 0.09 | 2.01 | 0.47 |  |
| At3G06930 | ATPRMT4B, PRMT4B, protein arginine methyltransferase 4B | 0.86 | 0.08 | 0.81 | 0.03 |  |
| At3G06962 | Other RNA | 1.82 | 0.49 | 2.74 | 0.44 |  |
| At3G06980 | DEA(D/H)-box RNA helicase family protein | 0.81 | 0.11 | 0.80 | 0.10 |  |
| At3G07160 | ATGSL10, CALS9, gsl10, GSL10, glucan synthase-like 10 | 1.07 | 0.05 | 0.78 | 0.08 |  |
| At3G07350 | Protein of unknown function (DUF506) | 0.54 | 0.07 | 4.40 | 0.09 |  |
| At3G07980 | MAP3KE2, MAPKKK6, mitogen-activated protein kinase kinase kinase 6 | 1.16 | 0.06 | 0.86 | 0.03 |  |
| At3G08960 | ARM repeat superfamily protein | 1.07 | 0.01 | 0.81 | 0.03 |  |
| At3G10160 | ATDFC, DFC, DHFS-FPGS homolog C | 0.80 | 0.06 | 0.77 | 0.03 |  |
| At3G10420 | P-loop containing nucleoside triphosphate hydrolases superfamily protein | 1.24 | 0.08 | 2.20 | 0.35 |  |
| At3G11690 | Unknown protein | 1.73 | 0.46 | 1.41 | 0.14 |  |
| At3G12500 | ATHCHIB, B-CHI, CHI-B, HCHIB, PR-3, PR3, basic chitinase | 0.52 | 0.05 | 3.05 | 0.75 |  |
| At3G12750 | ZIP1, zinc transporter 1 precursor | 0.79 | 0.07 | 1.64 | 0.16 |  |
| At3G12900 | 2-Oxoglutarate (2OG) and Fe(II)-dependent oxygenase superfamily protein | 612.44 | 199.47 | 0.06 | 0.10 |  |
| At3G12920 | SBP (S-ribonuclease binding protein) family protein | 1.77 | 0.23 | 0.75 | 0.04 |  |
| At3G12930 | Lojap-related protein | 0.78 | 0.08 | 0.81 | 0.11 |  |
| At3G12977 | NAC (No Apical Meristem) domain transcriptional regulator superfamily protein | 1.56 | 0.26 | 0.70 | 0.15 |  |
| At3G13100 | ATMRP7, MRP7, MRP7, multidrug resistance-associated protein 7 | 0.91 | 0.03 | 1.60 | 0.19 |  |
| At3G13110 | ATSERAT2;2, SAT-1, SAT-A, SAT-M, SAT3, SERAT2;2, serine acetyltransferase 2;2 | 0.83 | 0.08 | 2.12 | 0.22 |  |
| At3G13330 | PA200, proteasome activating protein 200 | 1.22 | 0.05 | 0.84 | 0.06 |  |
| At3G13610 | 2-Oxoglutarate (2OG) and Fe(II)-dependent oxygenase superfamily protein | 10.04 | 0.65 | 1.63 | 0.25 |  |
| At3G14440 | ATNCED3, NCED3, SIS7, STO1, nine-cis-epoxycarotenoid dioxygenase 3 | 0.51 | 0.24 | 0.42 | 0.06 |  |
| At3G14650 | CYP72A11, cytochrome P450, family 72, subfamily A, polypeptide 11 | 0.84 | 0.04 | 0.89 | 0.07 |  |
| At3G14690 | CYP72A15, cytochrome P450, family 72, subfamily A, polypeptide 15 | 0.70 | 0.07 | 0.90 | 0.06 |  |
| At3G15510 | ANAC056, ATNAC2, NAC2, NARS1, NAC domain containing protein 2 | 2.13 | 0.62 | 0.71 | 0.15 |  |
| At3G15700 | P-loop containing nucleoside triphosphate hydrolases superfamily protein | 1.51 | 0.16 | 1.29 | 0.16 |  |
| At3G15990 | SULTR3;4, sulfate transporter 3;4 | 1.12 | 0.06 | 1.84 | 0.11 |  |
| At3G16180 | Major facilitator superfamily protein | 0.77 | 0.03 | 0.87 | 0.06 |  |
| At3G16390 | NSP3, nitrile specifier protein 3 | 0.67 | 0.08 | 4.55 | 0.59 |  |
| At3G16830 | TPR2, TOPLESS-related 2 | 1.07 | 0.04 | 0.86 | 0.03 |  |
| At3G17710 | F-box and associated interaction domains-containing protein | 1.29 | 0.08 | 1.09 | 0.05 |  |
| At3G17770 | Dihydroxyacetone kinase | 1.32 | 0.13 | 0.83 | 0.08 |  |
| At3G17940 | Galactose mutarotase-like superfamily protein | 0.86 | 0.02 | 1.19 | 0.08 |  |
| At3G18070 | BGLU43, beta glucosidase 43 | 0.67 | 0.09 | 1.72 | 0.50 |  |
| At3G18080 | BGLU44, B-S glucosidase 44 | 0.85 | 0.05 | 1.70 | 0.12 |  |
| At3G18290 | BTS, EMB2454, zinc finger protein-related | 2.51 | 0.14 | 0.76 | 0.09 |  |
| At3G18390 | EMB1865, CRS1 / YhbY (CRM) domain-containing protein | 0.78 | 0.09 | 0.72 | 0.06 |  |
| At3G19380 | PUB25, plant U-box 25 | 0.68 | 0.03 | 0.79 | 0.07 |  |
| At3G19710 | BCAT4, branched-chain aminotransferase4 | 1.29 | 0.04 | 1.83 | 0.22 |  |
| At3G19810 | Protein of unknown function (DUF177) | 0.76 | 0.11 | 0.78 | 0.05 |  |
| At3G19970 | Alpha/beta-Hydrolases superfamily protein | 1.58 | 0.08 | 4.37 | 0.76 |  |
| At3G20570 | AtENODL9, ENODL9, early nodulin-like protein 9 | 0.93 | 0.01 | 0.82 | 0.05 |  |
| At3G20790 | NAD(P)-binding Rossmann-fold superfamily protein | 0.87 | 0.06 | 1.14 | 0.04 |  |
| At3G20840 | PLT1, Integrase-type DNA-binding superfamily protein | 0.77 | 0.06 | 1.14 | 0.08 |  |
| At3G21240 | 4CL2, AT4CL2, 4-coumarate:CoA ligase 2 | 3.73 | 0.16 | 1.25 | 0.09 |  |
| At3G21390 | Mitochondrial substrate carrier family protein | 0.64 | 0.05 | 0.73 | 0.03 |  |
| At3G21500 | DXPS1, 1-deoxy-D-xylulose 5-phosphate synthase 1 | 3.76 | 1.94 | 0.40 | 0.26 |  |
| At3G22890 | APS1, ATP sulfurylase 1 | 1.25 | 0.09 | 1.37 | 0.07 |  |
| At3G23000 | ATSR2, ATSRPK1, CIPK7, PKS7, SnRK3.10, CBL-interacting protein kinase 7 | 0.88 | 0.03 | 0.77 | 0.04 |  |
| At3G23570 | Alpha/beta-Hydrolases superfamily protein | 1.23 | 0.14 | 1.18 | 0.05 |  |
| At3G24180 | Beta-glucosidase, GBA2 type family protein | 1.10 | 0.01 | 0.89 | 0.01 |  |
| At3G24440 | VIL1, VRN5, Fibronectin type III domain-containing protein | 0.84 | 0.06 | 0.87 | 0.10 |  |
| At3G25790 | Myb-like transcription factor family protein | 0.65 | 0.12 | 1.94 | 0.29 |  |
| At3G25900 | ATHMT-1, HMT-1, Homocysteine S-methyltransferase family protein | 1.24 | 0.03 | 1.14 | 0.04 |  |
| At3G26450 | Polyketide cyclase/dehydrase and lipid transport superfamily protein | 0.87 | 0.06 | 1.30 | 0.12 |  |
| At3G27090 | DCD (Development and Cell Death) domain protein | 1.15 | 0.03 | 0.89 | 0.04 |  |
| At3G27460 | SGF29 tudor-like domain | 1.23 | 0.09 | 1.45 | 0.16 |  |
| At3G29280 | Unknown protein | 0.67 | 0.06 | 1.10 | 0.05 |  |
| At3G30260 | AGL79, AGAMOUS-like 79 | 0.59 | 0.15 | 1.52 | 0.17 |  |
| At3G32040 | Terpenoid synthases superfamily protein | 1.32 | 0.21 | 2.30 | 0.11 |  |
| At3G32980 | Peroxidase superfamily protein | 0.82 | 0.06 | 0.89 | 0.05 |  |
| At3G43610 | Spc97 / Spc98 family of spindle pole body (SBP) component | 0.85 | 0.04 | 0.82 | 0.09 |  |
| At3G44990 | ATXTR8, XTH31, XTR8, xyloglucan endo-transglycosylase-related 8 | 1.21 | 0.03 | 0.77 | 0.14 |  |
| At3G45780 | JK224, NPH1, PHOT1, RPT1, phototropin 1 | 0.74 | 0.10 | 0.65 | 0.03 |  |
| At3G46270 | Receptor protein kinase-related | 1.64 | 0.10 | 1.36 | 0.24 |  |
| At3G46800 | Cysteine/Histidine-rich C1 domain family protein | 0.65 | 0.05 | 0.55 | 0.01 |  |
| At3G46900 | COPT2, copper transporter 2 | 71.15 | 33.39 | 0.40 | 0.36 |  |
| At3G46960 | RNA helicase, ATP-dependent, SK12/DOB1 protein | 0.85 | 0.05 | 0.84 | 0.06 |  |
| At3G47420 | ATPS3, PS3, phosphate starvation-induced gene 3 | 2.24 | 0.27 | 12.82 | 5.53 |  |
| At3G48890 | ATMAPR3, ATMP2, MAPR3, MSBP2, membrane-associated progesterone binding protein 3 | 1.64 | 0.16 | 1.17 | 0.05 |  |
| At3G48990 | AMP-dependent synthetase and ligase family protein | 0.74 | 0.06 | 0.78 | 0.05 |  |
| At3G49390 | CID10, CTC-interacting domain 10 | 1.10 | 0.02 | 1.15 | 0.01 |  |
| At3G49960 | Peroxidase superfamily protein | 0.51 | 0.13 | 1.99 | 0.19 |  |
| At3G50710 | F-box/RNI-like/FBD-like domains-containing protein | 3.57 | 1.09 | 2.71 | 1.32 |  |
| At3G51000 | Alpha/beta-Hydrolases superfamily protein | 1.13 | 0.04 | 1.16 | 0.03 |  |
| At3G51570 | Disease resistance protein (TIR-NBS-LRR class) family | 2.32 | 0.58 | 2.70 | 1.18 |  |
| At3G52190 | PHF1, phosphate transporter traffic facilitator1 | 1.22 | 0.12 | 5.25 | 0.61 |  |
| At3G52720 | ACA1, ATACA1, CAH1, alpha carbonic anhydrase 1 | 0.40 | 0.20 | 5.56 | 3.11 |  |
| At3G52910 | AtGRF4, GRF4, growth-regulating factor 4 | 0.81 | 0.05 | 0.75 | 0.01 |  |
| At3G53620 | AtPPa4, PPa4, pyrophosphorylase 4 | 0.82 | 0.03 | 2.96 | 0.19 |  |
| At3G54830 | Transmembrane amino acid transporter family protein | 0.58 | 0.08 | 0.43 | 0.27 |  |
| At3G55840 | Hs1pro-1 protein | 0.80 | 0.07 | 0.51 | 0.08 |  |
| At3G56040 | UGP3, UDP-glucose pyrophosphorylase 3 | 0.74 | 0.18 | 4.98 | 0.71 |  |
| At3G56130 | Biotin/lipoyl attachment domain-containing protein | 0.89 | 0.04 | 1.22 | 0.01 |  |
| At3G56980 | BHLH039, ORG3, basic helix-loop-helix (bHLH) DNA-binding superfamily protein | 34.23 | 13.48 | 0.73 | 0.18 |  |
| At3G57157 | Other RNA | 4.28 | 0.37 | 9.81 | 1.46 |  |
| At3G57160 | Unknown protein | 2.57 | 0.55 | 1.46 | 0.23 |  |
| At3G57200 | Unknown protein | 0.63 | 0.10 | 0.74 | 0.03 |  |
| At3G57220 | Glycosyl transferase family 4 protein | 0.86 | 0.07 | 0.82 | 0.02 |  |
| At3G58990 | IPMI1, isopropylmalate isomerase 1 | 1.53 | 0.19 | 1.71 | 0.14 |  |
| At3G59300 | Pentatricopeptide repeat (PPR) superfamily protein | 1.21 | 0.05 | 1.22 | 0.03 |  |
| At3G59880 | Unknown protein | 4.58 | 2.42 | 2.51 | 0.54 |  |
| At3G60330 | AHA7, HA7, H(+)-ATPase 7 | 4.27 | 0.69 | 1.79 | 0.21 |  |
| At3G60740 | CHO, EMB133, TFC D, TTN1, ARM repeat superfamily protein | 0.87 | 0.08 | 0.81 | 0.02 |  |
| At3G61410 | BEST Arabidopsis thaliana protein match is: U-box domain-containing protein kinase family protein (TAIR:AT2G45910.1) | 6.53 | 1.32 | 3.79 | 0.43 |  |
| At3G62040 | Haloacid dehalogenase-like hydrolase (HAD) superfamily protein | 0.70 | 0.08 | 0.65 | 0.05 |  |
| At3G62060 | Pectinacetylesterase family protein | 0.78 | 0.14 | 0.78 | 0.13 |  |
| At3G62700 | ATMRP10, MRP10, multidrug resistance-associated protein 10 | 0.87 | 0.03 | 0.80 | 0.08 |  |
| At3G63190 | AtcpRRF, cpRRF, HFP108, RRF, ribosome recycling factor, chloroplast precursor | 0.86 | 0.05 | 0.80 | 0.06 |  |
| At4G00430 | PIP1;4, PIP1E, TMP-C, plasma membrane intrinsic protein 1;4 | 0.72 | 0.07 | 0.90 | 0.07 |  |
| At4G00910 | Aluminium activated malate transporter family protein | 1.66 | 0.13 | 0.46 | 0.22 |  |
| At4G01310 | Ribosomal L5P family protein | 0.81 | 0.05 | 0.84 | 0.03 |  |
| At4G01400 | Unknown protein | 0.88 | 0.02 | 0.94 | 0.03 |  |
| At4G01430 | Nodulin MtN21 /EamA-like transporter family protein | 0.30 | 0.03 | 0.48 | 0.19 |  |
| At4G01610 | Cysteine proteinases superfamily protein | 1.19 | 0.03 | 1.21 | 0.13 |  |
| At4G01800 | AGY1, AtcpSecA, Albino or Glassy Yellow 1 | 0.78 | 0.07 | 0.75 | 0.07 |  |
| At4G02110 | Transcription coactivators | 0.81 | 0.08 | 0.69 | 0.05 |  |
| At4G02290 | AtGH9B13, GH9B13, glycosyl hydrolase 9B13 | 0.90 | 0.04 | 0.77 | 0.05 |  |
| At4G02520 | ATGSTF2, ATPM24, ATPM24.1, GST2, GSTF2, glutathione S-transferase PHI 2 | 0.64 | 0.05 | 0.64 | 0.11 |  |
| At4G02890 | UBQ14, Ubiquitin family protein | 1.11 | 0.04 | 1.18 | 0.05 |  |
| At4G02940 | Oxidoreductase, 2OG-Fe(II) oxygenase family protein | 1.46 | 0.12 | 1.31 | 0.23 |  |
| At4G03340 | Core-2/I-branching beta-1,6-N-acetylglucosaminyltransferase family protein | 1.25 | 0.14 | 0.80 | 0.02 |  |
| At4G03500 | Ankyrin repeat family protein | 0.84 | 0.08 | 1.14 | 0.04 |  |
| At4G04040 | MEE51, Phosphofructokinase family protein | 0.83 | 0.04 | 2.50 | 0.22 |  |
| At4G04610 | APR, APR1, ATAPR1, PRH19, APS reductase 1 | 1.44 | 0.04 | 1.87 | 0.14 |  |
| At4G04850 | ATKEA3, KEA3, K+ efflux antiporter 3 | 0.68 | 0.11 | 0.82 | 0.12 |  |
| At4G04910 | NSF, AAA-type ATPase family protein | 0.95 | 0.02 | 0.84 | 0.05 |  |
| At4G05040 | Ankyrin repeat family protein | 1.25 | 0.16 | 1.19 | 0.05 |  |
| At4G09000 | GF14 CHI, GRF1, general regulatory factor 1 | 0.82 | 0.07 | 0.83 | 0.04 |  |
| At4G09040 | RNA-binding (RRM/RBD/RNP motifs) family protein | 0.77 | 0.13 | 0.76 | 0.03 |  |
| At4G09110 | RING/U-box superfamily protein | #DIV/0! | ###### | 0.77 | 0.04 |  |
| At4G10040 | CYTC-2, cytochrome c-2 | 0.69 | 0.04 | 1.32 | 0.11 |  |
| At4G10590 | UBP10, ubiquitin-specific protease 10 | 1.16 | 0.11 | 0.85 | 0.06 |  |
| At4G11150 | Emb2448, TUF, TUFF, VHA-E1, vacuolar ATP synthase subunit E1 | 0.92 | 0.04 | 1.13 | 0.06 |  |
| At4G11600 | ATGPX6, GPX6, LSC803, PHGPX, glutathione peroxidase 6 | 1.14 | 0.08 | 1.29 | 0.09 |  |
| At4G11650 | ATOSM34, OSM34, osmotin 34 | 0.67 | 0.10 | 2.16 | 0.38 |  |
| At4G12030 | BASS5, BAT5, bile acid transporter 5 | 1.34 | 0.19 | 1.45 | 0.08 |  |
| At4G12735 | Unknown protein | 2.67 | 1.20 | 1.87 | 0.47 |  |
| At4G13370 | Plant protein of unknown function (DUF936) | 0.87 | 0.01 | 0.85 | 0.06 |  |
| At4G13770 | CYP83A1, REF2, cytochrome P450, family 83, subfamily A, polypeptide 1 | 1.32 | 0.02 | 1.52 | 0.14 |  |
| At4G13970 | Zinc ion binding | 0.90 | 0.02 | 0.85 | 0.06 |  |
| At4G14310 | Transducin/WD40 repeat-like superfamily protein | 0.82 | 0.06 | 0.78 | 0.02 |  |
| At4G15560 | CLA, CLA1, DEF, DXPS2, DXS, Deoxyxylulose-5-phosphate synthase | 0.87 | 0.03 | 0.78 | 0.10 |  |
| At4G15810 | P-loop containing nucleoside triphosphate hydrolases superfamily protein | 0.76 | 0.07 | 0.62 | 0.12 |  |
| At4G15900 | PRL1, pleiotropic regulatory locus 1 | 0.93 | 0.06 | 0.82 | 0.04 |  |
| At4G16350 | CBL6, SCABP2, calcineurin B-like protein 6 | 1.47 | 0.20 | 1.68 | 0.07 |  |
| At4G16650 | O-fucosyltransferase family protein | 0.88 | 0.07 | 0.77 | 0.04 |  |
| At4G17140 | Pleckstrin homology (PH) domain-containing protein | 1.14 | 0.02 | 0.79 | 0.07 |  |
| At4G18480 | CH-42, CH42, CHL11, CHLI-1, CHLI1, P-loop containing nucleoside triphosphate hydrolases superfamily protein | 0.81 | 0.03 | 0.81 | 0.11 |  |
| At4G18600 | ATSCAR-LIKE, SCARL, WAVE5, SCAR family protein | 1.07 | 0.03 | 0.74 | 0.13 |  |
| At4G18700 | ATWL4, CIPK12, SnRK3.9, WL4, CBL-interacting protein kinase 12 | 1.91 | 0.10 | 1.68 | 0.11 |  |
| At4G19030 | AT-NLM1, ATNLM1, NIP1;1, NLM1, NOD26-like major intrinsic protein 1 | 0.83 | 0.03 | 1.23 | 0.13 |  |
| At4G19200 | Proline-rich family protein | 1.45 | 0.32 | 1.30 | 0.07 |  |
| At4G19690 | ATIRT1, IRT1, iron-regulated transporter 1 | 54.72 | 7.88 | 0.26 | 0.04 |  |
| At4G20070 | AAH, ATAAH, allantoate amidohydrolase | 0.83 | 0.04 | 0.61 | 0.11 |  |
| At4G20160 | RING/U-box superfamily protein (TAIR:AT1G30860.1) | 1.71 | 0.10 | 2.07 | 0.17 |  |
| At4G20320 | CTP synthase family protein | 0.83 | 0.04 | 0.77 | 0.04 |  |
| At4G20360 | ATRAB8D, ATRABE1B, RABE1b, RAB GTPase homolog E1B | 0.83 | 0.05 | 0.75 | 0.06 |  |
| At4G20430 | Subtilase family protein | 0.83 | 0.03 | 0.76 | 0.03 |  |
| At4G20820 | FAD-binding Berberine family protein | 0.66 | 0.19 | 0.79 | 0.06 |  |
| At4G20910 | CRM2, HEN1, double-stranded RNA binding protein-related / DsRBD protein-related | 0.79 | 0.08 | 0.78 | 0.03 |  |
| At4G21215 | Unknown protein | 1.60 | 0.20 | 1.46 | 0.27 |  |
| At4G21580 | Oxidoreductase, zinc-binding dehydrogenase family protein | 1.12 | 0.03 | 1.16 | 0.10 |  |
| At4G21960 | PRXR1, Peroxidase superfamily protein | 0.91 | 0.05 | 0.89 | 0.05 |  |
| At4G22250 | RING/U-box superfamily protein | 0.76 | 0.04 | 1.26 | 0.21 |  |
| At4G22860 | Cell cycle regulated microtubule associated protein | 1.28 | 0.10 | 0.83 | 0.04 |  |
| At4G22980 | Pyridoxal phosphate (PLP)-dependent transferases superfamily protein (TAIR:AT5G51920.1) | 2.30 | 0.28 | 0.56 | 0.14 |  |
| At4G23650 | CDPK6, CPK3, calcium-dependent protein kinase 6 | 0.93 | 0.05 | 0.92 | 0.01 |  |
| At4G23920 | ATUGE2, UGE2, UDP-D-glucose/UDP-D-galactose 4-epimerase 2 | 0.78 | 0.09 | 1.81 | 0.20 |  |
| At4G24100 | Protein kinase superfamily protein | 1.11 | 0.02 | 0.92 | 0.05 |  |
| At4G24670 | TAR2, tryptophan aminotransferase related 2 | 0.65 | 0.11 | 0.66 | 0.03 |  |
| At4G25100 | ATFSD1, FSD1, Fe superoxide dismutase 1 | 0.23 | 0.02 | 1.58 | 0.17 |  |
| At4G25150 | HAD superfamily, subfamily IIIB acid phosphatase | 0.72 | 0.07 | 1.48 | 0.15 |  |
| At4G26620 | Sucrase/ferredoxin-like family protein | 0.81 | 0.12 | 1.61 | 0.13 |  |
| At4G26890 | MAPKKK16, mitogen-activated protein kinase kinase kinase 16 | 2.16 | 0.42 | 3.00 | 0.85 |  |
| At4G27000 | ATRBP45C, RNA-binding (RRM/RBD/RNP motifs) family protein | 0.89 | 0.08 | 0.88 | 0.06 |  |
| At4G27230 | HTA2, histone H2A 2 | 0.85 | 0.08 | 1.20 | 0.04 |  |
| At4G27520 | AtENODL2, ENODL2, early nodulin-like protein 2 | 0.72 | 0.08 | 0.58 | 0.08 |  |
| At4G28710 | ATXIH, XIH, Myosin family protein with Dil domain | 1.11 | 0.06 | 0.89 | 0.05 |  |
| At4G29060 | Emb2726, elongation factor Ts family protein | 0.72 | 0.04 | 0.67 | 0.06 |  |
| At4G30310 | FGGY family of carbohydrate kinase | 0.94 | 0.04 | 0.87 | 0.05 |  |
| At4G30490 | AFG1-like ATPase family protein | 2.52 | 0.32 | 1.40 | 0.08 |  |
| At4G30630 | Unknown protein | 1.18 | 0.09 | 1.14 | 0.08 |  |
| At4G30670 | Putative membrane lipoprotein | 1.61 | 0.16 | 2.22 | 0.17 |  |
| At4G31940 | CYP82C4, cytochrome P450, family 82, subfamily C, polypeptide 4 | 184.70 | 3.63 | 0.08 | 0.01 |  |
| At4G32480 | Protein of unknown function (DUF506) | 1.35 | 0.19 | 2.85 | 1.36 |  |
| At4G33020 | ATZIP9, ZIP9, ZIP metal ion transporter family | 7.09 | 2.01 | 2.21 | 0.97 |  |
| At4G33090 | APM1, ATAPM1, aminopeptidase M1 | 0.87 | 0.00 | 0.81 | 0.04 |  |
| At4G34490 | ATCAP1, CAP 1, CAP1, cyclase associated protein 1 | 0.91 | 0.04 | 0.92 | 0.02 |  |
| At4G34580 | COW1, SRH1, Sec14p-like phosphatidylinositol transfer family protein | 1.39 | 0.16 | 1.81 | 0.20 |  |
| At4G34630 | Unknown protein | 0.89 | 0.06 | 1.18 | 0.06 |  |
| At4G34740 | ASE2, ATASE2, ATPURF2, CIA1, GLN phosphoribosyl pyrophosphate amidotransferase 2 | 0.69 | 0.11 | 0.74 | 0.07 |  |
| At4G34830 | MRL1, Pentatricopeptide repeat (PPR) superfamily protein | 0.69 | 0.10 | 0.68 | 0.07 |  |
| At4G35090 | CAT2, catalase 2 | 0.63 | 0.05 | 0.78 | 0.05 |  |
| At4G35440 | ATCLC-E, CLC-E, CLCE, chloride channel E | 0.55 | 0.06 | 0.74 | 0.16 |  |
| At4G35630 | PSAT, phosphoserine aminotransferase | 1.16 | 0.05 | 1.20 | 0.08 |  |
| At4G35720 | Arabidopsis protein of unknown function (DUF241) | 0.67 | 0.04 | 0.69 | 0.04 |  |
| At4G36060 | bHLH11, basic helix-loop-helix (bHLH) DNA-binding superfamily protein | 0.48 | 0.16 | 0.52 | 0.15 |  |
| At4G36180 | Leucine-rich receptor-like protein kinase family protein | 0.88 | 0.03 | 0.79 | 0.06 |  |
| At4G36430 | Peroxidase superfamily protein | 1.18 | 0.05 | 1.15 | 0.04 |  |
| At4G36440 | Unknown protein | 0.88 | 0.08 | 1.11 | 0.08 |  |
| At4G36670 | Major facilitator superfamily protein | 0.44 | 0.09 | 0.44 | 0.08 |  |
| At4G37410 | CYP81F4, cytochrome P450, family 81, subfamily F, polypeptide 4 | 0.79 | 0.02 | 0.76 | 0.05 |  |
| At4G37510 | Ribonuclease III family protein | 0.83 | 0.01 | 0.76 | 0.15 |  |
| At4G37890 | EDA40, Zinc finger (C3HC4-type RING finger) family protein | 0.89 | 0.05 | 0.80 | 0.14 |  |
| At4G37920 | Unknown protein | 0.78 | 0.06 | 0.80 | 0.10 |  |
| At4G38620 | ATMYB4, MYB4, myb domain protein 4 | 1.66 | 0.17 | 1.44 | 0.31 |  |
| At4G38950 | ATP binding microtubule motor family protein | 2.25 | 0.45 | 1.50 | 0.15 |  |
| At4G39420 | Unknown protein | 1.07 | 0.02 | 0.83 | 0.03 |  |
| At5G01060 | Protein kinase protein with tetratricopeptide repeat domain | 4.44 | 0.17 | 1.70 | 0.10 |  |
| At5G01220 | SQD2, sulfoquinovosyldiacylglycerol 2 | 0.86 | 0.06 | 10.49 | 3.02 |  |
| At5G01460 | LMBR1-like membrane protein | 0.93 | 0.03 | 0.87 | 0.05 |  |
| At5G01600 | ATFER1, FER1, ferretin 1 | 0.21 | 0.01 | 1.36 | 0.14 |  |
| At5G01720 | RNI-like superfamily protein | 1.38 | 0.17 | 1.78 | 0.26 |  |
| At5G01870 | Bifunctional inhibitor/lipid-transfer protein/seed storage 2S albumin superfamily protein | 0.71 | 0.10 | 2.05 | 0.51 |  |
| At5G01920 | STN8, Protein kinase superfamily protein | 0.84 | 0.05 | 0.79 | 0.09 |  |
| At5G02350 | Cysteine/Histidine-rich C1 domain family protein | 1.45 | 0.14 | 1.26 | 0.06 |  |
| At5G02380 | MT2B, metallothionein 2B | 1.12 | 0.03 | 1.35 | 0.16 |  |
| At5G02780 | GSTL1, glutathione transferase lambda 1 | 57.98 | 10.87 | 0.69 | 0.06 |  |
| At5G03730 | AtCTR1, CTR1, SIS1, Protein kinase superfamily protein | 1.15 | 0.05 | 0.96 | 0.01 |  |
| At5G04140 | FD-GOGAT, GLS1, GLU1, GLUS, glutamate synthase 1 | 0.63 | 0.13 | 0.54 | 0.15 |  |
| At5G04740 | ACT domain-containing protein | 1.14 | 0.06 | 1.24 | 0.07 |  |
| At5G04950 | ATNAS1, NAS1, nicotianamine synthase 1 | 4.39 | 0.50 | 0.49 | 0.01 |  |
| At5G05250 | Unknown protein | 6.62 | 0.95 | 0.57 | 0.10 |  |
| At5G05970 | NEDD1, Transducin/WD40 repeat-like superfamily protein | 0.87 | 0.04 | 0.81 | 0.04 |  |
| At5G06300 | Putative lysine decarboxylase family protein | 1.24 | 0.08 | 1.81 | 0.40 |  |
| At5G07460 | ATMSRA2, PMSR2, peptidemethionine sulfoxide reductase 2 | 1.24 | 0.18 | 1.24 | 0.01 |  |
| At5G07680 | ANAC079, ANAC080, ATNAC4, NAC080, NAC domain containing protein 80 | 0.81 | 0.02 | 0.65 | 0.10 |  |
| At5G08360 | Protein of unknown function (DUF789) | 0.67 | 0.04 | 0.64 | 0.17 |  |
| At5G10580 | Protein of unknown function, DUF599 | 0.75 | 0.04 | 0.74 | 0.03 |  |
| At5G10730 | NAD(P)-binding Rossmann-fold superfamily protein | 0.87 | 0.04 | 1.18 | 0.08 |  |
| At5G10740 | Protein phosphatase 2C family protein | 1.24 | 0.05 | 1.63 | 0.20 |  |
| At5G12250 | TUB6, beta-6 tubulin | 0.88 | 0.04 | 0.93 | 0.01 |  |
| At5G12370 | SEC10, exocyst complex component sec10 | 0.93 | 0.02 | 0.94 | 0.02 |  |
| At5G12400 | DNA binding;zinc ion binding;DNA binding | 1.12 | 0.02 | 0.78 | 0.10 |  |
| At5G12850 | CCCH-type zinc finger protein with ARM repeat domain | 0.83 | 0.04 | 0.87 | 0.07 |  |
| At5G13750 | ZIFL1, zinc induced facilitator-like 1 | 1.19 | 0.07 | 1.44 | 0.15 |  |
| At5G13910 | LEP, Integrase-type DNA-binding superfamily protein | 2.14 | 0.15 | 1.25 | 0.16 |  |
| At5G13980 | Glycosyl hydrolase family 38 protein | 0.83 | 0.05 | 0.77 | 0.11 |  |
| At5G14200 | ATIMD1, IMD1, isopropylmalate dehydrogenase 1 | 1.10 | 0.01 | 1.50 | 0.06 |  |
| At5G15830 | AtbZIP3, bZIP3, basic leucine-zipper 3 | 0.75 | 0.07 | 0.76 | 0.05 |  |
| At5G16590 | LRR1, Leucine-rich repeat protein kinase family protein | 0.87 | 0.07 | 0.88 | 0.04 |  |
| At5G16790 | AtTHO7, THO7, Tho complex subunit 7/Mft1p | 0.66 | 0.18 | 0.73 | 0.01 |  |
| At5G17820 | Peroxidase superfamily protein | 1.32 | 0.17 | 1.52 | 0.30 |  |
| At5G18700 | EMB3013, RUK, Protein kinase family protein with ARM repeat domain | 0.85 | 0.06 | 0.72 | 0.01 |  |
| At5G18860 | Inosine-uridine preferring nucleoside hydrolase family protein | 0.86 | 0.01 | 0.80 | 0.04 |  |
| At5G18900 | 2-Oxoglutarate (2OG) and Fe(II)-dependent oxygenase superfamily protein | 1.24 | 0.12 | 1.10 | 0.04 |  |
| At5G19560 | ATROPGEF10, ROPGEF10, ROP uanine nucleotide exchange factor 10 | 5.33 | 0.51 | 2.12 | 0.20 |  |
| At5G19600 | SULTR3;5, sulfate transporter 3;5 | 0.47 | 0.09 | 0.62 | 0.13 |  |
| At5G19770 | TUA3, tubulin alpha-3 | 0.84 | 0.06 | 0.86 | 0.05 |  |
| At5G19780 | TUA5, tubulin alpha-5 | 0.83 | 0.06 | 0.85 | 0.03 |  |
| At5G19970 | Unknown protein | 2.81 | 0.43 | 0.57 | 0.07 |  |
| At5G20140 | SOUL heme-binding family protein | 0.72 | 0.05 | 1.91 | 0.34 |  |
| At5G20280 | ATSPS1F, SPS1F, sucrose phosphate synthase 1F | 0.77 | 0.06 | 2.05 | 0.17 |  |
| At5G20400 | 2-Oxoglutarate (2OG) and Fe(II)-dependent oxygenase superfamily protein | 1.18 | 0.09 | 1.67 | 0.12 |  |
| At5G20610 | Unknown protein | 1.14 | 0.04 | 0.85 | 0.04 |  |
| At5G20660 | Zn-dependent exopeptidases superfamily protein | 1.18 | 0.05 | 0.82 | 0.05 |  |
| At5G20790 | Unknown protein | 0.32 | 0.13 | 90.27 | 24.26 |  |
| At5G21222 | protein kinase family protein | 0.77 | 0.04 | 0.72 | 0.12 |  |
| At5G22555 | Unknown protein | 2.37 | 0.58 | 4.13 | 2.07 |  |
| At5G22800 | EMB1030, EMB263, EMB86, Alanyl-tRNA synthetase, class IIc | 1.62 | 0.21 | 0.85 | 0.10 |  |
| At5G22890 | C2H2 and C2HC zinc fingers superfamily protein | 2.32 | 0.53 | 1.91 | 0.25 |  |
| At5G23010 | IMS3, MAM1, methylthioalkylmalate synthase 1 | 1.28 | 0.01 | 1.75 | 0.19 |  |
| At5G23020 | IMS2, MAM-L, MAM3, 2-isopropylmalate synthase 2 | 1.30 | 0.10 | 1.33 | 0.08 |  |
| At5G23150 | HUA2, Tudor/PWWP/MBT domain-containing protein | 1.10 | 0.03 | 0.86 | 0.05 |  |
| At5G23290 | PFD5, prefoldin 5 | 0.85 | 0.05 | 1.19 | 0.10 |  |
| At5G23955 | Transposable element gene | 1.34 | 0.27 | 1.17 | 0.01 |  |
| At5G24070 | Peroxidase superfamily protein | 0.77 | 0.15 | 1.51 | 0.02 |  |
| At5G24090 | ATCHIA, CHIA, chitinase A | 1.59 | 0.23 | 1.41 | 0.38 |  |
| At5G24270 | ATSOS3, CBL4, SOS3, Calcium-binding EF-hand family protein | 1.15 | 0.02 | 1.23 | 0.13 |  |
| At5G24460 | Unknown protein | 1.21 | 0.08 | 1.20 | 0.12 |  |
| At5G24810 | ABC1 family protein | 1.24 | 0.16 | 0.86 | 0.07 |  |
| At5G26320 | TRAF-like family protein | 2.24 | 0.26 | 2.95 | 0.37 |  |
| At5G26742 | Emb1138, DEAD box RNA helicase (RH3) | 0.81 | 0.09 | 0.74 | 0.10 |  |
| At5G26820 | ATIREG3, IREG3, IREG3, MAR1, RTS3, iron-regulated protein 3 | 2.54 | 0.28 | 0.77 | 0.19 |  |
| At5G27920 | F-box family protein | 2.14 | 0.15 | 1.29 | 0.15 |  |
| At5G35630 | ATGSL1, GLN2, GS2, glutamine synthetase 2 | 1.06 | 0.02 | 0.90 | 0.01 |  |
| At5G35970 | P-loop containing nucleoside triphosphate hydrolases superfamily protein | 0.83 | 0.01 | 0.72 | 0.06 |  |
| At5G36870 | ATGSL09, atgsl9, gsl09, GSL09, glucan synthase-like 9 | 0.54 | 0.06 | 0.40 | 0.10 |  |
| At5G37020 | ARF8, ATARF8, auxin response factor 8 | 0.89 | 0.03 | 0.77 | 0.04 |  |
| At5G37740 | Calcium-dependent lipid-binding (CaLB domain) family protein | 1.31 | 0.09 | 1.28 | 0.13 |  |
| At5G37990 | S-adenosyl-L-methionine-dependent methyltransferases superfamily protein | 0.85 | 0.06 | 0.73 | 0.03 |  |
| At5G38820 | Transmembrane amino acid transporter family protein | 54.35 | 11.69 | 0.43 | 0.09 |  |
| At5G39500 | ERMO1, GNL1, GNOM-like 1 | 0.92 | 0.04 | 0.82 | 0.07 |  |
| At5G39950 | ATH2, ATTRX2, ATTRXH2, TRX2, TRXH2, thioredoxin 2 | 1.14 | 0.05 | 1.18 | 0.04 |  |
| At5G40510 | Sucrase/ferredoxin-like family protein | 0.59 | 0.01 | 1.23 | 0.08 |  |
| At5G40850 | UPM1, urophorphyrin methylase 1 | 0.68 | 0.01 | 0.76 | 0.04 |  |
| At5G40860 | Unknown protein | 1.51 | 0.11 | 2.32 | 0.81 |  |
| At5G41140 | Myosin heavy chain-related protein | 1.20 | 0.10 | 0.83 | 0.06 |  |
| At5G41790 | CIP1, COP1-interactive protein 1 | 1.62 | 0.02 | 0.81 | 0.07 |  |
| At5G42325 | Transcription factor IIS protein | 0.51 | 0.21 | 0.19 | 0.18 |  |
| At5G42710 | Unknown protein | 1.17 | 0.05 | 0.64 | 0.08 |  |
| At5G43180 | Protein of unknown function, DUF599 | 0.71 | 0.02 | 0.68 | 0.06 |  |
| At5G44720 | Molybdenum cofactor sulfurase family protein | 1.12 | 0.04 | 1.19 | 0.08 |  |
| At5G45380 | ATDUR3, DUR3, solute:sodium symporters;urea transmembrane transporters | 0.81 | 0.04 | 0.68 | 0.05 |  |
| At5G45510 | Leucine-rich repeat (LRR) family protein | 1.38 | 0.08 | 0.79 | 0.07 |  |
| At5G47730 | Sec14p-like phosphatidylinositol transfer family protein | 1.19 | 0.06 | 1.41 | 0.09 |  |
| At5G47870 | Cobalt ion binding (TAIR:AT1G71310.1) | 1.91 | 0.18 | 1.19 | 0.04 |  |
| At5G48290 | Heavy metal transport/detoxification superfamily protein | 1.67 | 0.46 | 1.45 | 0.17 |  |
| At5G48410 | ATGLR1.3, GLR1.3, glutamate receptor 1.3 | 1.56 | 0.20 | 1.33 | 0.23 |  |
| At5G48657 | Defense protein-related | 3.33 | 0.71 | 1.36 | 0.23 |  |
| At5G48910 | LPA66, Pentatricopeptide repeat (PPR) superfamily protein | 0.69 | 0.11 | 0.77 | 0.09 |  |
| At5G48930 | HCT, hydroxycinnamoyl-CoA shikimate/quinate hydroxycinnamoyl transferase | 2.04 | 0.17 | 1.16 | 0.07 |  |
| At5G49440 | Unknown protein | 0.83 | 0.03 | 0.78 | 0.01 |  |
| At5G49770 | Leucine-rich repeat protein kinase family protein | 0.75 | 0.10 | 1.78 | 0.10 |  |
| At5G49820 | EMB1879, RUS6, Protein of unknown function, DUF647 | 0.87 | 0.04 | 0.92 | 0.03 |  |
| At5G50400 | ATPAP27, PAP27, purple acid phosphatase 27 | 0.81 | 0.06 | 1.18 | 0.11 |  |
| At5G50920 | ATHSP93-V, CLPC, CLPC1, DCA1, HSP93-V, CLPC homologue 1 | 0.94 | 0.02 | 0.86 | 0.07 |  |
| At5G51600 | ATMAP65-3, MAP65-3, PLE, Microtubule associated protein (MAP65/ASE1) family protein | 0.84 | 0.09 | 0.78 | 0.03 |  |
| At5G51890 | Peroxidase superfamily protein | 0.84 | 0.12 | 0.82 | 0.05 |  |
| At5G52790 | CBS domain-containing protein with a domain of unknown function (DUF21) | 0.67 | 0.11 | 0.51 | 0.09 |  |
| At5G53140 | Protein phosphatase 2C family protein | 1.14 | 0.08 | 0.82 | 0.03 |  |
| At5G53330 | Ubiquitin-associated/translation elongation factor EF1B protein | 1.21 | 0.06 | 1.21 | 0.12 |  |
| At5G53420 | CCT motif family protein | 1.20 | 0.03 | 1.69 | 0.13 |  |
| At5G53480 | ARM repeat superfamily protein | 1.06 | 0.01 | 0.87 | 0.00 |  |
| At5G53550 | ATYSL3, YSL3, YELLOW STRIPE like 3 | 0.53 | 0.03 | 1.11 | 0.02 |  |
| At5G53620 | Unknown protein | 0.87 | 0.04 | 0.85 | 0.10 |  |
| At5G53850 | Haloacid dehalogenase-like hydrolase family protein | 2.09 | 0.06 | 0.96 | 0.01 |  |
| At5G54040 | Cysteine/Histidine-rich C1 domain family protein | 0.82 | 0.04 | 1.54 | 0.24 |  |
| At5G54160 | ATOMT1, OMT1, O-methyltransferase 1 | 0.72 | 0.08 | 1.20 | 0.10 |  |
| At5G54230 | AtMYB49, MYB49, myb domain protein 49 | 0.74 | 0.09 | 0.83 | 0.10 |  |
| At5G54300 | Protein of unknown function (DUF761) | 0.53 | 0.13 | 0.50 | 0.10 |  |
| At5G54670 | ATK3, KATC, kinesin 3 | 1.22 | 0.07 | 1.07 | 0.01 |  |
| At5G54680 | bHLH105, ILR3, basic helix-loop-helix (bHLH) DNA-binding superfamily protein | 1.32 | 0.11 | 1.29 | 0.11 |  |
| At5G54790 | Unknown protein | 3.20 | 0.75 | 2.02 | 0.19 |  |
| At5G54800 | ATGPT1, GPT1, glucose 6-phosphate/phosphate translocator 1 | 1.41 | 0.13 | 1.30 | 0.11 |  |
| At5G55480 | SVL1, SHV3-like 1 | 0.83 | 0.02 | 0.77 | 0.05 |  |
| At5G55560 | Protein kinase superfamily protein | 1.49 | 0.04 | 1.33 | 0.12 |  |
| At5G56290 | ATPEX5, PEX5, peroxin 5 | 0.88 | 0.03 | 0.86 | 0.03 |  |
| At5G56660 | ILL2, IAA-leucine resistant (ILR)-like 2 | 1.36 | 0.19 | 1.18 | 0.04 |  |
| At5G57480 | P-loop containing nucleoside triphosphate hydrolases superfamily protein | 1.31 | 0.04 | 0.81 | 0.04 |  |
| At5G57540 | AtXTH13, XTH13, xyloglucan endotransglucosylase/hydrolase 13 | 1.53 | 0.11 | 2.33 | 0.36 |  |
| At5G58330 | Lactate/malate dehydrogenase family protein | 0.58 | 0.07 | 0.81 | 0.02 |  |
| At5G58550 | EOL2, ETO1-like 2 | 0.81 | 0.04 | 0.88 | 0.08 |  |
| At5G59080 | Unknown protein | 0.41 | 0.12 | 0.45 | 0.04 |  |
| At5G59340 | WOX2, WUSCHEL related homeobox 2 | 0.21 | 0.25 | 0.32 | 0.20 |  |
| At5G59520 | ZIP2, ZRT/IRT-like protein 2 | 0.16 | 0.04 | 1.85 | 0.32 |  |
| At5G59770 | Protein-tyrosine phosphatase-like, PTPLA | 0.84 | 0.05 | 1.11 | 0.01 |  |
| At5G60410 | ATSIZ1, SIZ1, DNA-binding protein with MIZ/SP-RING zinc finger, PHD-finger and SAP domain | 1.11 | 0.04 | 0.94 | 0.01 |  |
| At5G61040 | Unknown protein | 0.88 | 0.03 | 0.82 | 0.10 |  |
| At5G62900 | Unknown protein | 1.23 | 0.03 | 0.83 | 0.03 |  |
| At5G62960 | Unknown protein | 0.79 | 0.12 | 0.71 | 0.14 |  |
| At5G63790 | ANAC102, NAC102, NAC domain containing protein 102 | 1.18 | 0.08 | 1.19 | 0.07 |  |
| At5G63940 | Protein kinase protein with adenine nucleotide alpha hydrolases-like domain | 0.90 | 0.08 | 0.82 | 0.06 |  |
| At5G65010 | ASN2, asparagine synthetase 2 | 0.76 | 0.03 | 0.70 | 0.11 |  |
| At5G65380 | MATE efflux family protein | 1.36 | 0.09 | 0.81 | 0.05 |  |
| At5G65500 | U-box domain-containing protein kinase family protein | 2.73 | 0.60 | 6.26 | 0.42 |  |
| At5G65640 | bHLH093, beta HLH protein 93 | 0.92 | 0.05 | 1.29 | 0.11 |  |
| At5G66470 | RNA binding; GTP binding | 0.74 | 0.10 | 0.81 | 0.03 |  |
| At5G66530 | Galactose mutarotase-like superfamily protein | 0.83 | 0.08 | 1.34 | 0.06 |  |

If the transcript of a gene was not determined (read number =zero) is one the biological repeats under control condition, no fold change was available in this repeat, resulting in no any number for the final mean, which indicated as ‘-’
