# Supplementary figures and images for: Genome-wide analysis of overlapping genes regulated by iron deficiency and phosphate starvation reveals new interactions in Arabidopsis roots
Source: BMC Res Notes. 2015 Oct 12;8:555. doi: 10.1186/s13104-015-1524-y (PMC4604098; doi:10.1186/s13104-015-1524-y)

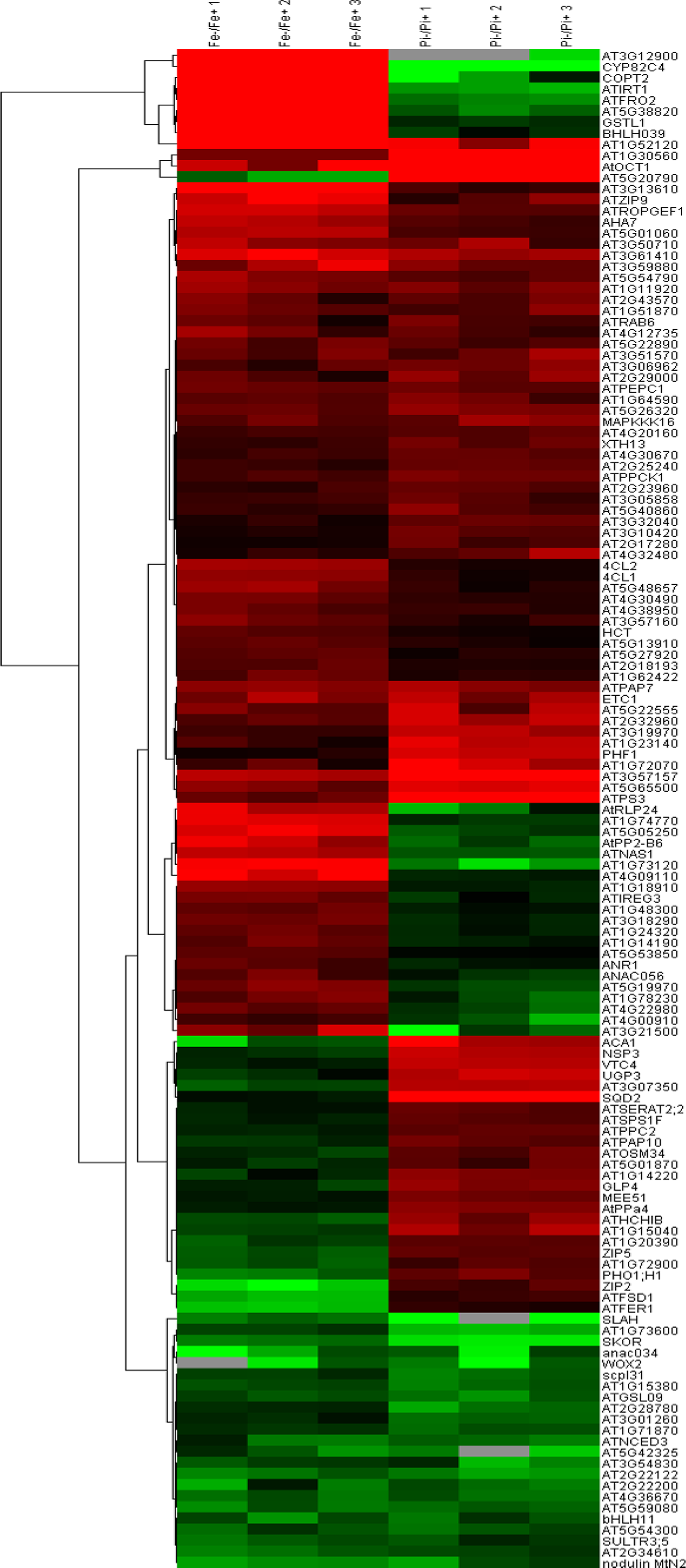

Supplement: Supplementary file 2 — 10.1186/s13104-015-1524-y Hierarchical cluster analysis of 579 overlapping genes with greater than twofold changes in transcript abundance in Arabidopsis roots grown under Fe- or Pi-deficient conditions. [file 13104_2015_1524_MOESM2_ESM.tif]

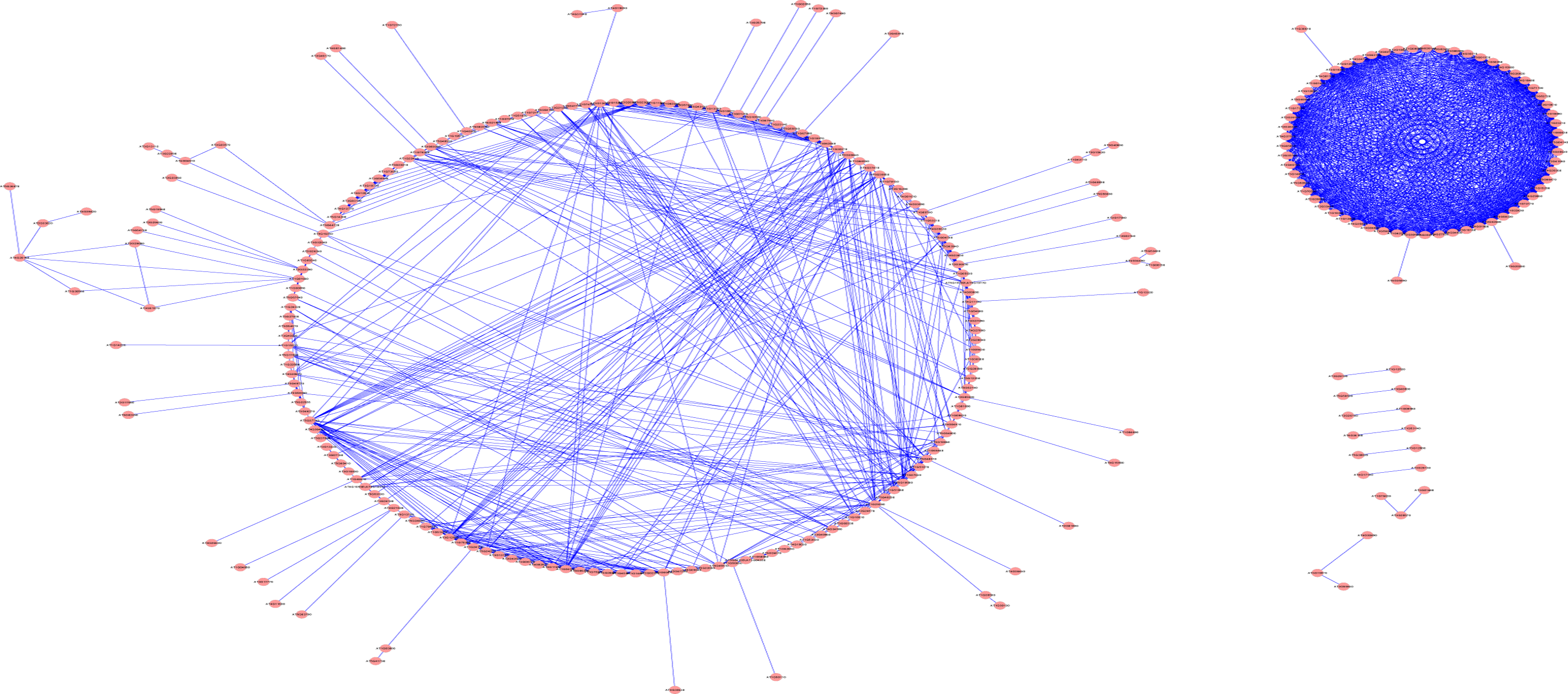

Supplement: Supplementary file 5 — 10.1186/s13104-015-1524-y Co-expression relationships of the 579 differentially expressed overlapping genes. [file 13104_2015_1524_MOESM5_ESM.tif]

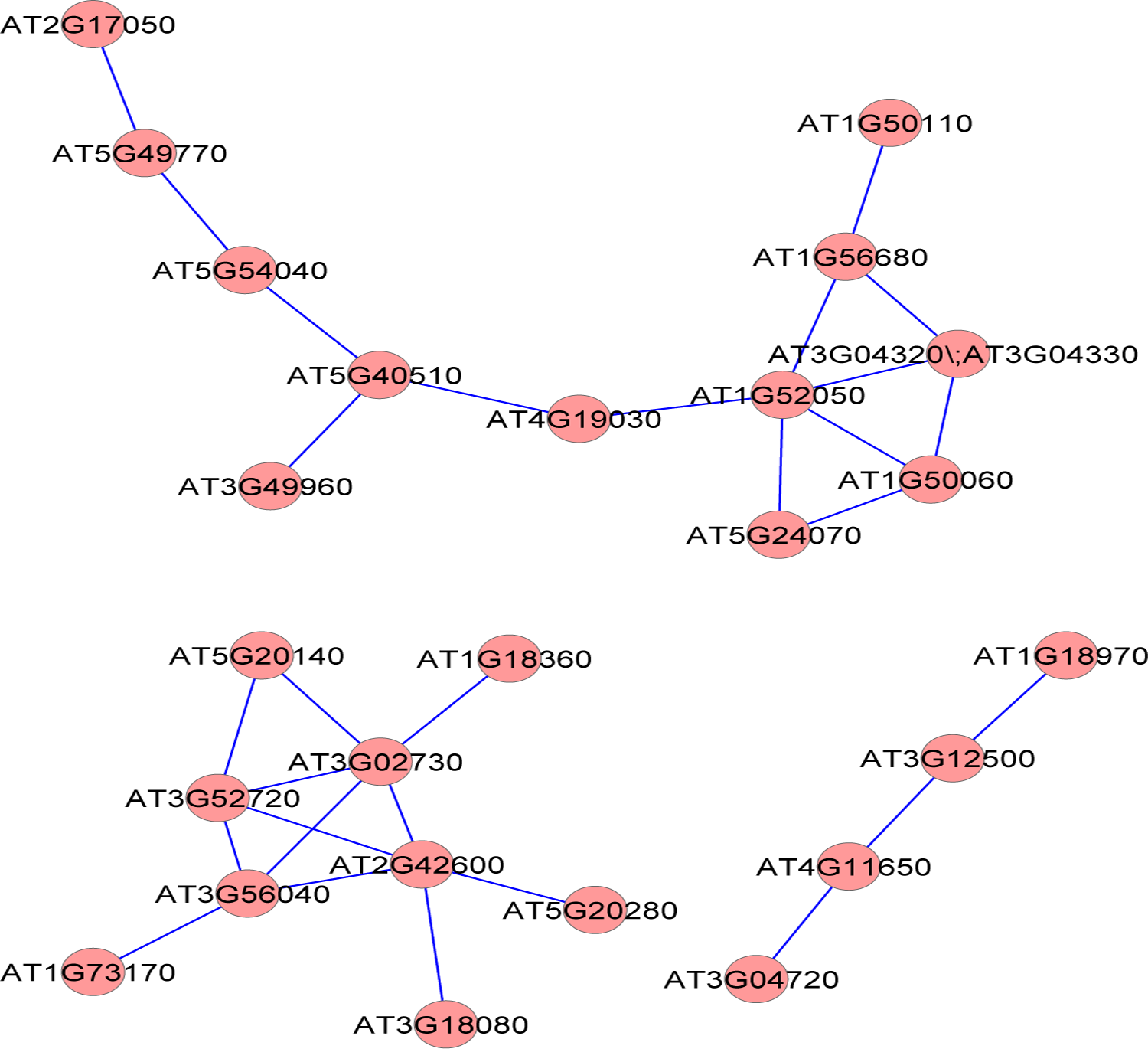

Supplement: Supplementary file 8 — 10.1186/s13104-015-1524-y Co-expression relationships of the 97 differentially expressed overlapping genes induced by Pi starvation but repressed by Fe deficiency. [file 13104_2015_1524_MOESM8_ESM.tif]
