# Supplementary material for: Genome-wide analysis of overlapping genes regulated by iron deficiency and phosphate starvation reveals new interactions in Arabidopsis roots
Source: BMC Res Notes. 2015 Oct 12;8:555. doi: 10.1186/s13104-015-1524-y (PMC4604098; doi:10.1186/s13104-015-1524-y)
Supplement: Supplementary file 3 — 10.1186/s13104-015-1524-y Gene Ontology enrichment was assessed using GOBU (Lin et al. [66]) in the 579 overlapping genes (elim, P < 0.01). In the term type column, P, F and C indicate biological process, functional process and subcellular localization, respectively. [file 13104_2015_1524_MOESM3_ESM.doc]

**Additional file 3** Gene Ontology enrichment was assessed using GOBU (Lin et al., 2006) in the 579 overlapping genes (elim, P<0.01). In the term type column, P, F and C indicate biological process, functional process and subcellular localization, respectively.

| **GOID** | **Term Type** | **P-value(elim)** | **GO name** |
| --- | --- | --- | --- |
| GO:0009651 | P | 1.38E-05 | response to salt stress |
| GO:0006979 | P | 2.14E-05 | response to oxidative stress |
| GO:0019761 | P | 3.77E-05 | glucosinolate biosynthetic process |
| GO:0010731 | P | 4.96E-05 | protein glutathionylation |
| GO:0010043 | P | 5.13E-05 | response to zinc ion |
| GO:0009793 | P | 5.45E-05 | embryo development ending in seed dormancy |
| GO:0046686 | P | 7.23E-05 | response to cadmium ion |
| GO:0055085 | P | 8.82E-05 | transmembrane transport |
| GO:0007017 | P | 1.25E-04 | microtubule-based process |
| GO:0071281 | P | 1.26E-04 | cellular response to iron ion |
| GO:0071732 | P | 1.35E-04 | cellular response to nitric oxide |
| GO:0009611 | P | 3.40E-04 | response to wounding |
| GO:0009098 | P | 3.96E-04 | leucine biosynthetic process |
| GO:0042221 | P | 4.91E-04 | response to chemical stimulus |
| GO:0016036 | P | 5.83E-04 | cellular response to phosphate starvation |
| GO:0009624 | P | 6.34E-04 | response to nematode |
| GO:0006879 | P | 7.58E-04 | cellular iron ion homeostasis |
| GO:0007009 | P | 8.79E-04 | plasma membrane organization |
| GO:0006996 | P | 9.41E-04 | organelle organization |
| GO:0006817 | P | 0.001279696 | phosphate transport |
| GO:0006829 | P | 0.001279696 | zinc ion transport |
| GO:0071369 | P | 0.001879483 | cellular response to ethylene stimulus |
| GO:0019748 | P | 0.001951322 | secondary metabolic process |
| GO:0050832 | P | 0.002111457 | defense response to fungus |
| GO:0006826 | P | 0.002411173 | iron ion transport |
| GO:0006012 | P | 0.002411173 | galactose metabolic process |
| GO:0080022 | P | 0.002411173 | primary root development |
| GO:0046394 | P | 0.002715584 | carboxylic acid biosynthetic process |
| GO:0006605 | P | 0.003040525 | protein targeting |
| GO:0015995 | P | 0.003319672 | chlorophyll biosynthetic process |
| GO:0009409 | P | 0.003546359 | response to cold |
| GO:0015698 | P | 0.003672046 | inorganic anion transport |
| GO:0050801 | P | 0.004196272 | ion homeostasis |
| GO:0042742 | P | 0.004210512 | defense response to bacterium |
| GO:0010359 | P | 0.004246399 | regulation of anion channel activity |
| GO:0000096 | P | 0.004451133 | sulfur amino acid metabolic process |
| GO:0005985 | P | 0.004451133 | sucrose metabolic process |
| GO:0006790 | P | 0.004944139 | sulfur compound metabolic process |
| GO:0000911 | P | 0.005372263 | cytokinesis by cell plate formation |
| GO:0006520 | P | 0.005402166 | cellular amino acid metabolic process |
| GO:0007584 | P | 0.005877287 | response to nutrient |
| GO:0009616 | P | 0.005877287 | virus induced gene silencing |
| GO:0048580 | P | 0.005929301 | regulation of post-embryonic development |
| GO:0009309 | P | 0.006336735 | amine biosynthetic process |
| GO:0048367 | P | 0.006820478 | shoot development |
| GO:0019751 | P | 0.006867376 | polyol metabolic process |
| GO:0080167 | P | 0.007159355 | response to karrikin |
| GO:0006995 | P | 0.007747311 | cellular response to nitrogen starvation |
| GO:0019430 | P | 0.007747311 | removal of superoxide radicals |
| GO:0010109 | P | 0.00786929 | regulation of photosynthesis |
| GO:0044262 | P | 0.008272904 | cellular carbohydrate metabolic process |
| GO:0071554 | P | 0.008643246 | cell wall organization or biogenesis |
| GO:0006813 | P | 0.008666298 | potassium ion transport |
| GO:0009610 | P | 0.009847774 | response to symbiotic fungus |
| GO:0051701 | P | 0.009847774 | interaction with host |
| GO:0004674 | F | 6.12E-05 | protein serine/threonine kinase activity |
| GO:0004601 | F | 7.51E-05 | peroxidase activity |
| GO:0005385 | F | 7.61E-05 | zinc ion transmembrane transporter activity |
| GO:0005524 | F | 8.37E-05 | ATP binding |
| GO:0005375 | F | 1.02E-04 | copper ion transmembrane transporter activity |
| GO:0008061 | F | 2.67E-04 | chitin binding |
| GO:0010177 | F | 2.96E-04 | 2-(2'-methylthio)ethylmalate synthase activity |
| GO:0015631 | F | 6.70E-04 | tubulin binding |
| GO:0004034 | F | 7.58E-04 | aldose 1-epimerase activity |
| GO:0005215 | F | 8.18E-04 | transporter activity |
| GO:0008661 | F | 8.79E-04 | 1-deoxy-D-xylulose-5-phosphate synthase activity |
| GO:0042277 | F | 0.00147181 | peptide binding |
| GO:0008757 | F | 0.001652643 | S-adenosylmethionine-dependent methyltransferase activity |
| GO:0016661 | F | 0.001737986 | oxidoreductase activity, acting on other nitrogenous compounds as donors |
| GO:0008964 | F | 0.001737986 | phosphoenolpyruvate carboxylase activity |
| GO:0045174 | F | 0.001737986 | glutathione dehydrogenase (ascorbate) activity |
| GO:0022804 | F | 0.001819111 | active transmembrane transporter activity |
| GO:0015075 | F | 0.001845293 | ion transmembrane transporter activity |
| GO:0016887 | F | 0.001977332 | ATPase activity |
| GO:0008483 | F | 0.001990816 | transaminase activity |
| GO:0005515 | F | 0.002013157 | protein binding |
| GO:0003824 | F | 0.002020552 | catalytic activity |
| GO:0016879 | F | 0.003051939 | ligase activity, forming carbon-nitrogen bonds |
| GO:0003989 | F | 0.004246399 | acetyl-CoA carboxylase activity |
| GO:0008526 | F | 0.004246399 | phosphatidylinositol transporter activity |
| GO:0005086 | F | 0.004246399 | ARF guanyl-nucleotide exchange factor activity |
| GO:0004866 | F | 0.004451133 | endopeptidase inhibitor activity |
| GO:0005507 | F | 0.005101919 | copper ion binding |
| GO:0003993 | F | 0.006867376 | acid phosphatase activity |
| GO:0004784 | F | 0.007747311 | superoxide dismutase activity |
| GO:0015370 | F | 0.007747311 | solute:sodium symporter activity |
| GO:0004568 | F | 0.00786929 | chitinase activity |
| GO:0016836 | F | 0.008101142 | hydro-lyase activity |
| GO:0004176 | F | 0.0088295 | ATP-dependent peptidase activity |
| GO:0008172 | F | 0.009847774 | S-methyltransferase activity |
| GO:0005886 | C | 5.37E-15 | plasma membrane |
| GO:0009570 | C | 3.21E-11 | chloroplast stroma |
| GO:0048046 | C | 1.13E-10 | apoplast |
| GO:0009507 | C | 1.80E-06 | chloroplast |
| GO:0005773 | C | 4.04E-06 | vacuole |
| GO:0005618 | C | 1.13E-04 | cell wall |
| GO:0005829 | C | 6.96E-04 | cytosol |
| GO:0009941 | C | 7.38E-04 | chloroplast envelope |
| GO:0016020 | C | 0.001930473 | membrane |
| GO:0044430 | C | 0.003492807 | cytoskeletal part |
| GO:0009535 | C | 0.003784428 | chloroplast thylakoid membrane |
