# Supplementary material for: Genome-wide analysis of overlapping genes regulated by iron deficiency and phosphate starvation reveals new interactions in Arabidopsis roots
Source: BMC Res Notes. 2015 Oct 12;8:555. doi: 10.1186/s13104-015-1524-y (PMC4604098; doi:10.1186/s13104-015-1524-y)
Supplement: Supplementary file 4 — 10.1186/s13104-015-1524-y Representative Gene Ontology categories (in biological process) enriched in the 137 overlapping genes with twofold change in expression. [file 13104_2015_1524_MOESM4_ESM.doc]

**Additional file 4** Gene Ontology enrichment was assessed using GOBU (Lin et al., 2006) in the 137 overlapping genes with 2-fold change in expression (elim, P<0.01). In the term type column, P, F and C indicate biological process, functional process and subcellular localization, respectively.

| **GOID** | **Term Type** | **P-value(elim)** | **GO name** |
| --- | --- | --- | --- |
| GO:0071732 | P | 4.63E-07 | cellular response to nitric oxide |
| GO:0071281 | P | 5.80E-06 | cellular response to iron ion |
| GO:0006879 | P | 1.07E-05 | cellular iron ion homeostasis |
| GO:0071369 | P | 1.44E-05 | cellular response to ethylene stimulus |
| GO:0006817 | P | 0.00125 | phosphate transport |
| GO:0006829 | P | 0.00125 | zinc ion transport |
| GO:0010038 | P | 0.001667 | response to metal ion |
| GO:0006826 | P | 0.001673 | iron ion transport |
| GO:0006099 | P | 0.001907 | tricarboxylic acid cycle |
| GO:0015794 | P | 0.004077 | glycerol-3-phosphate transport |
| GO:0010654 | P | 0.004077 | apical cell fate commitment |
| GO:0015678 | P | 0.004077 | high-affinity copper ion transport |
| GO:0015688 | P | 0.004077 | iron chelate transport |
| GO:0016036 | P | 0.004802 | cellular response to phosphate starvation |
| GO:0009620 | P | 0.005605 | response to fungus |
| GO:0044262 | P | 0.005715 | cellular carbohydrate metabolic process |
| GO:0009698 | P | 0.005834 | phenylpropanoid metabolic process |
| GO:0000097 | P | 0.007555 | sulfur amino acid biosynthetic process |
| GO:0010421 | P | 0.008138 | hydrogen peroxide-mediated programmed cell death |
| GO:0046506 | P | 0.008138 | sulfolipid biosynthetic process |
| GO:0010106 | P | 0.008138 | cellular response to iron ion starvation |
| GO:0008964 | F | 9.85E-05 | phosphoenolpyruvate carboxylase activity |
| GO:0005375 | F | 0.00125 | copper ion transmembrane transporter activity |
| GO:0016207 | F | 0.00125 | 4-coumarate-CoA ligase activity |
| GO:0005385 | F | 0.001454 | zinc ion transmembrane transporter activity |
| GO:0004573 | F | 0.004077 | mannosyl-oligosaccharide glucosidase activity |
| GO:0015169 | F | 0.004077 | glycerol-3-phosphate transmembrane transporter activity |
| GO:0043874 | F | 0.004077 | acireductone synthase activity |
| GO:0047172 | F | 0.004077 | shikimate O-hydroxycinnamoyltransferase activity |
| GO:0046510 | F | 0.004077 | UDP-sulfoquinovose:DAG sulfoquinovosyltransferase activity |
| GO:0046524 | F | 0.004077 | sucrose-phosphate synthase activity |
| GO:0047205 | F | 0.004077 | quinate O-hydroxycinnamoyltransferase activity |
| GO:0046570 | F | 0.004077 | methylthioribulose 1-phosphate dehydratase activity |
| GO:0015226 | F | 0.004077 | carnitine transporter activity |
| GO:0004568 | F | 0.004294 | chitinase activity |
| GO:0008324 | F | 0.005615 | cation transmembrane transporter activity |
| GO:0051213 | F | 0.006661 | dioxygenase activity |
| GO:0010347 | F | 0.008138 | L-galactose-1-phosphate phosphatase activity |
| GO:0015088 | F | 0.008138 | copper uptake transmembrane transporter activity |
| GO:0016021 | C | 0.001831 | integral to membrane |
| GO:0005886 | C | 0.007492 | plasma membrane |
| GO:0010318 | C | 0.008138 | pyrophosphate-dependent phosphofructokinase complex,  beta-subunit complex |

Lin W-D, Chen Y-C, Ho J-M, Hsiao C-D: **GOBU: toward an integration interface for biological objects**. *Journal of information science and engineering* 2006, **22**(1):19.
