# Supplementary material for: Genome-wide analysis of overlapping genes regulated by iron deficiency and phosphate starvation reveals new interactions in Arabidopsis roots
Source: BMC Res Notes. 2015 Oct 12;8:555. doi: 10.1186/s13104-015-1524-y (PMC4604098; doi:10.1186/s13104-015-1524-y)
Supplement: Supplementary file 6 — 10.1186/s13104-015-1524-y Subset of 210 overlapping genes consists of the largest co-expression module. The fold change of the gene expression was indicated as mean with standard deviation (SD). [file 13104_2015_1524_MOESM6_ESM.doc]

**Additional file 6** Subset of 210 overlapping genes consists of the largest co-expression module. The fold change of the gene expression was indicated as mean with standard deviation (SD).

| **AGI** | **Annotation** | **Mean(-Fe/+Fe)** | **SD** | **Mean(-Pi-/+Pi)** | **SD** |
| --- | --- | --- | --- | --- | --- |
| At1G01090 | PDH-E1 ALPHA, pyruvate dehydrogenase E1 alpha | 0.84 | 0.01 | 0.88 | 0.03 |
| At1G01660 | RING/U-box superfamily protein | 1.49 | 0.11 | 1.58 | 0.2 |
| At1G03080 | Kinase interacting (KIP1-like) family protein | 1.37 | 0.03 | 0.86 | 0.09 |
| At1G04680 | Pectin lyase-like superfamily protein | 0.81 | 0.04 | 0.8 | 0.08 |
| At1G04850 | ubiquitin-associated (UBA)/TS-N Domain-containing protein | 1.11 | 0.01 | 1.05 | 0.03 |
| At1G07590 | Tetratricopeptide repeat (TPR)-like Superfamily protein | 1.7 | 0.18 | 1.24 | 0.1 |
| At1G08190 | ATVAM2, ATVPS41, VAM2, VPS41, ZIP2, Vacuolar protein sorting 41 | 1.07 | 0.02 | 0.89 | 0.02 |
| At1G09430 | ACLA-3, ATP-citrate lyase A-3 | 1.29 | 0.06 | 0.88 | 0.03 |
| At1G09620 | ATP binding;leucine-tRNA ligases;aminoacyl-tRNA ligases;nucleotide binding;ATP binding;aminoacyl-tRNA Ligases | 0.87 | 0.05 | 0.74 | 0.1 |
| At1G09780 | Phosphoglycerate mutase, 2,3-Bisphosphoglycerate-independent | 1.33 | 0.03 | 1.18 | 0.04 |
| At1G11080 | Scpl31, serine carboxypeptidase-like 31 | 0.63 | 0.08 | 0.42 | 0.08 |
| At1G13300 | HRS1, myb-like transcription factor family protein | 1.19 | 0.08 | 1.33 | 0.15 |
| At1G13320 | PP2AA3, protein phosphatase 2A subunit A3 | 0.91 | 0.04 | 0.93 | 0.05 |
| At1G14220 | Ribonuclease T2 family protein | 0.74 | 0.18 | 2.89 | 0.27 |
| At1G14870 | PCR2, PLANT CADMIUM RESISTANCE 2 | 1.47 | 0.2 | 1.66 | 0.17 |
| At1G15210 | ATPDR7, PDR7, pleiotropic drug resistance 7 | 0.87 | 0 | 0.77 | 0.06 |
| At1G15380 | Lactoylglutathione lyase / glyoxalase I family protein | 0.55 | 0.04 | 0.43 | 0.09 |
| At1G18910 | Zinc ion binding;zinc ion binding | 3.33 | 0.13 | 0.78 | 0.04 |
| At1G18970 | GLP4, germin-like protein 4 | 0.71 | 0.12 | 3.04 | 0.41 |
| At1G19570 | ATDHAR1, DHAR1, DHAR5, dehydroascorbate reductase | 0.86 | 0.06 | 1.2 | 0.07 |
| At1G22410 | Class-II DAHP synthetase family protein | 1.55 | 0.13 | 1.32 | 0.07 |
| At1G22500 | RING/U-box superfamily protein | 0.78 | 0.1 | 0.7 | 0.05 |
| At1G23140 | Calcium-dependent lipid-binding (CaLB domain) family protein | 1.58 | 0.43 | 5.33 | 1.19 |
| At1G24150 | ATFH4, FH4, formin homologue 4 | 1.16 | 0.06 | 1.19 | 0.06 |
| At1G24320 | Six-hairpin glycosidases superfamily protein | 2.26 | 0.27 | 0.78 | 0.09 |
| At1G27030 | Unknown protein | 1.4 | 0.09 | 1.34 | 0.07 |
| At1G29280 | ATWRKY65, WRKY65, WRKY DNA-binding protein 65 | 1.51 | 0.05 | 1.23 | 0.12 |
| At1G30130 | Unknown protein | 0.77 | 0.05 | 0.79 | 0.07 |
| At1G30270 | ATCIPK23, CIPK23, LKS1, SnRK3.23, CBL-interacting protein kinase 23 | 0.85 | 0.1 | 0.87 | 0.06 |
| At1G30360 | ERD4, Early-responsive to dehydration stress protein (ERD4) | 0.88 | 0.04 | 0.78 | 0.08 |
| At1G30560 | Major facilitator superfamily protein | - | - | 55.93 | 32.45 |
| At1G30850 | RSH4, root hair specific 4 | 1.38 | 0.14 | 1.52 | 0.21 |
| At1G35580 | CINV1, cytosolic invertase 1 | 1.33 | 0.1 | 0.72 | 0.03 |
| At1G43710 | Emb1075, Pyridoxal phosphate (PLP)-dependent transferases superfamily protein | 0.84 | 0.03 | 0.76 | 0.05 |
| At1G45145 | ATH5, ATTRX5, LIV1, TRX5, thioredoxin H-type 5 | 1.95 | 0.27 | 1.59 | 0.26 |
| At1G48300 | Unknown protein | 2.27 | 0.25 | 0.85 | 0.07 |
| At1G48410 | AGO1, Stabilizer of iron transporter SufD / Polynucleotidyl transferase | 0.85 | 0.09 | 0.79 | 0.08 |
| At1G50060 | CAP (Cysteine-rich secretory proteins, Antigen 5, and Pathogenesis-related 1 protein) superfamily protein | 0.71 | 0.04 | 1.42 | 0.05 |
| At1G50110 | D-aminoacid aminotransferase-like PLP-dependent enzymes superfamily protein | 0.55 | 0.02 | 1.62 | 0.09 |
| At1G51070 | bHLH115, basic helix-loop-helix (bHLH) DNA-binding superfamily protein | 1.26 | 0.02 | 1.11 | 0.04 |
| At1G51420 | ATSPP1, SPP1, sucrose-phosphatase 1 | 1.24 | 0.12 | 1.4 | 0.24 |
| At1G51680 | 4CL.1, 4CL1, AT4CL1, 4-coumarate:CoA ligase 1 | 3.22 | 0.06 | 1.27 | 0.19 |
| At1G51860 | Leucine-rich repeat protein kinase family protein | 1.97 | 0.32 | 1.2 | 0.05 |
| At1G52050 | Mannose-binding lectin superfamily protein | 0.7 | 0.04 | 1.32 | 0.11 |
| At1G53310 | ATPEPC1, ATPPC1, PEPC1, PPC1, phosphoenolpyruvate carboxylase 1 | 2.34 | 0.19 | 2.21 | 0.25 |
| At1G53830 | ATPME2, PME2, pectin methylesterase 2 | 0.79 | 0.12 | 0.74 | 0.08 |
| At1G56145 | Leucine-rich repeat transmembrane protein kinase | 1.21 | 0.05 | 0.85 | 0.03 |
| At1G56680 | Chitinase family protein | 0.68 | 0.04 | 1.36 | 0.1 |
| At1G62280 | SLAH1, SLAC1 homologue 1 | 0.4 | 0.06 | 0.04 | 0.04 |
| At1G64590 | NAD(P)-binding Rossmann-fold superfamily protein | 2.06 | 0.13 | 2.39 | 0.7 |
| At1G65690 | Late embryogenesis abundant (LEA) hydroxyproline-rich glycoprotein family | 1.75 | 0.18 | 1.47 | 0.22 |
| At1G65840 | ATPAO4, PAO4, polyamine oxidase 4 | 1.74 | 0.07 | 1.79 | 0.34 |
| At1G65930 | cICDH, cytosolic NADP+-dependent isocitrate dehydrogenase | 0.94 | 0.01 | 0.93 | 0.01 |
| At1G67800 | Copine (Calcium-dependent phospholipid-binding protein) family | 1.54 | 0.19 | 1.51 | 0.17 |
| At1G67940 | ATNAP3, AtSTAR1, NAP3, NAP3, non-intrinsic ABC protein 3 | 1.38 | 0.1 | 1.33 | 0.17 |
| At1G69240 | ATMES15, MES15, RHS9, methyl esterase 15 | 1.17 | 0.04 | 1.64 | 0.16 |
| At1G70300 | KUP6, K+ uptake permease 6 | 1.32 | 0.17 | 1.32 | 0.08 |
| At1G72150 | PATL1, PATELLIN 1 | 0.87 | 0.04 | 0.79 | 0.03 |
| At1G72160 | Sec14p-like phosphatidylinositol transfer family protein | 0.83 | 0.02 | 0.9 | 0.03 |
| At1G72300 | Leucine-rich receptor-like protein kinase family protein | 1.26 | 0.03 | 0.77 | 0.03 |
| At1G73600 | S-adenosyl-L-methionine-dependent methyltransferases superfamily protein | 0.61 | 0.06 | 0.23 | 0.02 |
| At1G74770 | Zinc ion binding | 6.43 | 0.5 | 0.66 | 0.07 |
| At1G75270 | DHAR2, dehydroascorbate reductase 2 | 1.14 | 0.02 | 1.38 | 0.09 |
| At1G78150 | Unknown protein | 0.93 | 0.05 | 1.3 | 0 |
| At1G78300 | 14-3-3OMEGA, GF14 OMEGA, GRF2, general regulatory factor 2 | 0.86 | 0.05 | 0.92 | 0.06 |
| At1G79450 | ALIS5, ALA-interacting subunit 5 | 1.55 | 0.21 | 1.24 | 0.1 |
| At2G01880 | ATPAP7, PAP7, purple acid phosphatase 7 | 3.14 | 0.21 | 3.31 | 0.81 |
| At2G02390 | ATGSTZ1, GST18, GSTZ1, glutathione S-transferase zeta 1 | 1.16 | 0.04 | 1.21 | 0.09 |
| At2G05920 | Subtilase family protein | 0.87 | 0.01 | 0.74 | 0.07 |
| At2G15620 | ATHNIR, NIR, NIR1, nitrite reductase 1 | 0.63 | 0.01 | 0.67 | 0.06 |
| At2G17050 | disease resistance protein (TIR-NBS-LRR class), putative | 0.6 | 0.05 | 1.19 | 0.12 |
| At2G20990 | ATSYTA, NTMC2T1.1, NTMC2TYPE1.1, SYT1, SYTA, synaptotagmin A | 0.9 | 0.03 | 0.87 | 0.02 |
| At2G22290 | ATRAB-H1D, ATRAB6, ATRABH1D, RAB-H1D, RABH1d, RAB GTPase homolog H1D | 1.91 | 0.66 | 2.1 | 0.59 |
| At2G23960 | Class I glutamine amidotransferase-like superfamily protein | 1.55 | 0.27 | 2.13 | 0.21 |
| At2G24570 | ATWRKY17, WRKY17, WRKY DNA-binding protein 17 | 1.15 | 0.05 | 0.85 | 0.02 |
| At2G25240 | Serine protease inhibitor (SERPIN) family protein | 1.53 | 0.15 | 2.27 | 0.08 |
| At2G28780 | Unknown protein | 0.72 | 0.03 | 0.36 | 0.1 |
| At2G28840 | XBAT31, XB3 ortholog 1 in Arabidopsis thaliana | 0.85 | 0.02 | 0.88 | 0.04 |
| At2G29000 | Leucine-rich repeat protein kinase family protein | 1.85 | 0.6 | 3.03 | 0.79 |
| At2G29620 | Unknown protein | 1.47 | 0.13 | 1.34 | 0.26 |
| At2G30840 | 2-oxoglutarate (2OG) and Fe(II)-dependent oxygenase superfamily protein | 1.53 | 0.15 | 1.24 | 0.06 |
| At2G33020 | AtRLP24, RLP24, receptor like protein 24 | 5.05 | 1.74 | 0.48 | 0.33 |
| At2G36530 | ENO2, LOS2, Enolase | 1.29 | 0.1 | 1.26 | 0.05 |
| At2G43570 | CHI, chitinase, putative | 2.22 | 0.87 | 2.26 | 0.45 |
| At2G45910 | U-box domain-containing protein kinase family protein | 0.91 | 0.03 | 1.93 | 0.17 |
| At2G46170 | Reticulon family protein | 1.12 | 0.1 | 1.18 | 0.06 |
| At2G47260 | ATWRKY23, WRKY23, WRKY DNA-binding protein 23 | 1.46 | 0.04 | 0.67 | 0.05 |
| At3G01260 | Galactose mutarotase-like superfamily protein | 0.76 | 0.09 | 0.44 | 0.03 |
| At3G02850 | SKOR, STELAR K+ outward rectifier | 0.39 | 0.03 | 0.15 | 0.01 |
| At3G03160 | FUNCTIONS IN: molecular_function unknown; INVOLVED IN: intracellular protein transport | 0.83 | 0.05 | 1.21 | 0.12 |
| At3G03190 | ATGSTF11, ATGSTF6, GSTF11, glutathione S-transferase F11 | 1.44 | 0.11 | 1.93 | 0.15 |
| At3G03250 | AtUGP1, UGP, UGP1, UDP-GLUCOSE PYROPHOSPHORYLASE 1 | 0.9 | 0.05 | 1.19 | 0.04 |
| At3G04320 | Kunitz family trypsin and protease inhibitor protein | 0.74 | 0.05 | 1.91 | 0.14 |
| At3G04330 | Kunitz family trypsin and protease inhibitor protein | 0.7 | 0.03 | 1.94 | 0.43 |
| At3G04720 | HEL, PR-4, PR4, pathogenesis-related 4 | 0.62 | 0.09 | 1.76 | 0.33 |
| At3G07160 | ATGSL10, CALS9, gsl10, GSL10, glucan synthase-like 10 | 1.07 | 0.05 | 0.78 | 0.08 |
| At3G12500 | ATHCHIB, B-CHI, CHI-B, HCHIB, PR-3, PR3, basic chitinase | 0.52 | 0.05 | 3.05 | 0.75 |
| At3G13100 | ATMRP7, MRP7, MRP7, multidrug resistance-associated protein 7 | 0.91 | 0.03 | 1.6 | 0.19 |
| At3G13110 | ATSERAT2;2, SAT-1, SAT-A, SAT-M, SAT3, SERAT2;2, serine acetyltransferase 2;2 | 0.83 | 0.08 | 2.12 | 0.22 |
| At3G13330 | PA200, proteasome activating protein 200 | 1.22 | 0.05 | 0.84 | 0.06 |
| At3G13610 | 2-Oxoglutarate (2OG) and Fe(II)-dependent oxygenase superfamily protein | 10.04 | 0.65 | 1.63 | 0.25 |
| At3G16390 | NSP3, nitrile specifier protein 3 | 0.67 | 0.08 | 4.55 | 0.59 |
| At3G16830 | TPR2, TOPLESS-related 2 | 1.07 | 0.04 | 0.86 | 0.03 |
| At3G17770 | Dihydroxyacetone kinase | 1.32 | 0.13 | 0.83 | 0.08 |
| At3G17940 | Galactose mutarotase-like superfamily protein | 0.86 | 0.02 | 1.19 | 0.08 |
| At3G19710 | BCAT4, branched-chain aminotransferase4 | 1.29 | 0.04 | 1.83 | 0.22 |
| At3G21240 | 4CL2, AT4CL2, 4-coumarate:CoA ligase 2 | 3.73 | 0.16 | 1.25 | 0.09 |
| At3G22890 | APS1, ATP sulfurylase 1 | 1.25 | 0.09 | 1.37 | 0.07 |
| At3G23570 | Alpha/beta-Hydrolases superfamily protein | 1.23 | 0.14 | 1.18 | 0.05 |
| At3G24180 | Beta-glucosidase, GBA2 type family protein | 1.1 | 0.01 | 0.89 | 0.01 |
| At3G25790 | Myb-like transcription factor family protein | 0.65 | 0.12 | 1.94 | 0.29 |
| At3G27090 | DCD (Development and Cell Death) domain protein | 1.15 | 0.03 | 0.89 | 0.04 |
| At3G32040 | Terpenoid synthases superfamily protein | 1.32 | 0.21 | 2.3 | 0.11 |
| At3G46270 | Receptor protein kinase-related | 1.64 | 0.1 | 1.36 | 0.24 |
| At3G48890 | ATMAPR3, ATMP2, MAPR3, MSBP2, membrane-associated progesterone binding protein 3 | 1.64 | 0.16 | 1.17 | 0.05 |
| At3G49390 | CID10, CTC-interacting domain 10 | 1.1 | 0.02 | 1.15 | 0.01 |
| At3G49960 | Peroxidase superfamily protein | 0.51 | 0.13 | 1.99 | 0.19 |
| At3G51570 | Disease resistance protein (TIR-NBS-LRR class) family | 2.32 | 0.58 | 2.7 | 1.18 |
| At3G58990 | IPMI1, isopropylmalate isomerase 1 | 1.53 | 0.19 | 1.71 | 0.14 |
| At3G60330 | AHA7, HA7, H(+)-ATPase 7 | 4.27 | 0.69 | 1.79 | 0.21 |
| At3G62040 | Haloacid dehalogenase-like hydrolase (HAD) superfamily protein | 0.7 | 0.08 | 0.65 | 0.05 |
| At4G01400 | Unknown protein | 0.88 | 0.02 | 0.94 | 0.03 |
| At4G01610 | Cysteine proteinases superfamily protein | 1.19 | 0.03 | 1.21 | 0.13 |
| At4G02520 | ATGSTF2, ATPM24, ATPM24.1, GST2, GSTF2, glutathione S-transferase PHI 2 | 0.64 | 0.05 | 0.64 | 0.11 |
| At4G04610 | APR, APR1, ATAPR1, PRH19, APS reductase 1 | 1.44 | 0.04 | 1.87 | 0.14 |
| At4G04910 | NSF, AAA-type ATPase family protein | 0.95 | 0.02 | 0.84 | 0.05 |
| At4G09000 | GF14 CHI, GRF1, general regulatory factor 1 | 0.82 | 0.07 | 0.83 | 0.04 |
| At4G10590 | UBP10, ubiquitin-specific protease 10 | 1.16 | 0.11 | 0.85 | 0.06 |
| At4G11150 | Emb2448, TUF, TUFF, VHA-E1, vacuolar ATP synthase subunit E1 | 0.92 | 0.04 | 1.13 | 0.06 |
| At4G11600 | ATGPX6, GPX6, LSC803, PHGPX, glutathione peroxidase 6 | 1.14 | 0.08 | 1.29 | 0.09 |
| At4G11650 | ATOSM34, OSM34, osmotin 34 | 0.67 | 0.1 | 2.16 | 0.38 |
| At4G12030 | BASS5, BAT5, bile acid transporter 5 | 1.34 | 0.19 | 1.45 | 0.08 |
| At4G13770 | CYP83A1, REF2, cytochrome P450, family 83, subfamily A, polypeptide 1 | 1.32 | 0.02 | 1.52 | 0.14 |
| At4G15900 | PRL1, pleiotropic regulatory locus 1 | 0.93 | 0.06 | 0.82 | 0.04 |
| At4G16350 | CBL6, SCABP2, calcineurin B-like protein 6 | 1.47 | 0.2 | 1.68 | 0.07 |
| At4G19030 | AT-NLM1, ATNLM1, NIP1;1, NLM1, NOD26-like major intrinsic protein 1 | 0.83 | 0.03 | 1.23 | 0.13 |
| At4G19690 | ATIRT1, IRT1, iron-regulated transporter 1 | 54.72 | 7.88 | 0.26 | 0.04 |
| At4G20160 | RING/U-box superfamily protein (TAIR:AT1G30860.1) | 1.71 | 0.1 | 2.07 | 0.17 |
| At4G21580 | Oxidoreductase, zinc-binding dehydrogenase family protein | 1.12 | 0.03 | 1.16 | 0.1 |
| At4G21960 | PRXR1, Peroxidase superfamily protein | 0.91 | 0.05 | 0.89 | 0.05 |
| At4G23650 | CDPK6, CPK3, calcium-dependent protein kinase 6 | 0.93 | 0.05 | 0.92 | 0.01 |
| At4G26620 | Sucrase/ferredoxin-like family protein | 0.81 | 0.12 | 1.61 | 0.13 |
| At4G27000 | ATRBP45C, RNA-binding (RRM/RBD/RNP motifs) family protein | 0.89 | 0.08 | 0.88 | 0.06 |
| At4G30630 | Unknown protein | 1.18 | 0.09 | 1.14 | 0.08 |
| At4G30670 | Putative membrane lipoprotein | 1.61 | 0.16 | 2.22 | 0.17 |
| At4G31940 | CYP82C4, cytochrome P450, family 82, subfamily C, polypeptide 4 | 184.7 | 3.63 | 0.08 | 0.01 |
| At4G33090 | APM1, ATAPM1, aminopeptidase M1 | 0.87 | 0 | 0.81 | 0.04 |
| At4G34490 | ATCAP1, CAP 1, CAP1, cyclase associated protein 1 | 0.91 | 0.04 | 0.92 | 0.02 |
| At4G34580 | COW1, SRH1, Sec14p-like phosphatidylinositol transfer family protein | 1.39 | 0.16 | 1.81 | 0.2 |
| At4G35630 | PSAT, phosphoserine aminotransferase | 1.16 | 0.05 | 1.2 | 0.08 |
| At4G36430 | Peroxidase superfamily protein | 1.18 | 0.05 | 1.15 | 0.04 |
| At4G36440 | Unknown protein | 0.88 | 0.08 | 1.11 | 0.08 |
| At4G37410 | CYP81F4, cytochrome P450, family 81, subfamily F, polypeptide 4 | 0.79 | 0.02 | 0.76 | 0.05 |
| At4G39420 | Unknown protein | 1.07 | 0.02 | 0.83 | 0.03 |
| At5G02350 | Cysteine/Histidine-rich C1 domain family protein | 1.45 | 0.14 | 1.26 | 0.06 |
| At5G02380 | MT2B, metallothionein 2B | 1.12 | 0.03 | 1.35 | 0.16 |
| At5G04740 | ACT domain-containing protein | 1.14 | 0.06 | 1.24 | 0.07 |
| At5G06300 | Putative lysine decarboxylase family protein | 1.24 | 0.08 | 1.81 | 0.4 |
| At5G07460 | ATMSRA2, PMSR2, peptidemethionine sulfoxide reductase 2 | 1.24 | 0.18 | 1.24 | 0.01 |
| At5G10580 | Protein of unknown function, DUF599 | 0.75 | 0.04 | 0.74 | 0.03 |
| At5G12250 | TUB6, beta-6 tubulin | 0.88 | 0.04 | 0.93 | 0.01 |
| At5G13750 | ZIFL1, zinc induced facilitator-like 1 | 1.19 | 0.07 | 1.44 | 0.15 |
| At5G14200 | ATIMD1, IMD1, isopropylmalate dehydrogenase 1 | 1.1 | 0.01 | 1.5 | 0.06 |
| At5G17820 | Peroxidase superfamily protein | 1.32 | 0.17 | 1.52 | 0.3 |
| At5G18860 | Inosine-uridine preferring nucleoside hydrolase family protein | 0.86 | 0.01 | 0.8 | 0.04 |
| At5G18900 | 2-Oxoglutarate (2OG) and Fe(II)-dependent oxygenase superfamily protein | 1.24 | 0.12 | 1.1 | 0.04 |
| At5G19560 | ATROPGEF10, ROPGEF10, ROP uanine nucleotide exchange factor 10 | 5.33 | 0.51 | 2.12 | 0.2 |
| At5G19770 | TUA3, tubulin alpha-3 | 0.84 | 0.06 | 0.86 | 0.05 |
| At5G19780 | TUA5, tubulin alpha-5 | 0.83 | 0.06 | 0.85 | 0.03 |
| At5G19970 | Unknown protein | 2.81 | 0.43 | 0.57 | 0.07 |
| At5G20400 | 2-Oxoglutarate (2OG) and Fe(II)-dependent oxygenase superfamily protein | 1.18 | 0.09 | 1.67 | 0.12 |
| At5G22555 | Unknown protein | 2.37 | 0.58 | 4.13 | 2.07 |
| At5G22890 | C2H2 and C2HC zinc fingers superfamily protein | 2.32 | 0.53 | 1.91 | 0.25 |
| At5G23010 | IMS3, MAM1, methylthioalkylmalate synthase 1 | 1.28 | 0.01 | 1.75 | 0.19 |
| At5G23020 | IMS2, MAM-L, MAM3, 2-isopropylmalate synthase 2 | 1.3 | 0.1 | 1.33 | 0.08 |
| At5G24070 | Peroxidase superfamily protein | 0.77 | 0.15 | 1.51 | 0.02 |
| At5G24090 | ATCHIA, CHIA, chitinase A | 1.59 | 0.23 | 1.41 | 0.38 |
| At5G24270 | ATSOS3, CBL4, SOS3, Calcium-binding EF-hand family protein | 1.15 | 0.02 | 1.23 | 0.13 |
| At5G27920 | F-box family protein | 2.14 | 0.15 | 1.29 | 0.15 |
| At5G36870 | ATGSL09, atgsl9, gsl09, GSL09, glucan synthase-like 9 | 0.54 | 0.06 | 0.4 | 0.1 |
| At5G37740 | Calcium-dependent lipid-binding (CaLB domain) family protein | 1.31 | 0.09 | 1.28 | 0.13 |
| At5G37990 | S-adenosyl-L-methionine-dependent methyltransferases superfamily protein | 0.85 | 0.06 | 0.73 | 0.03 |
| At5G39950 | ATH2, ATTRX2, ATTRXH2, TRX2, TRXH2, thioredoxin 2 | 1.14 | 0.05 | 1.18 | 0.04 |
| At5G40510 | Sucrase/ferredoxin-like family protein | 0.59 | 0.01 | 1.23 | 0.08 |
| At5G40850 | UPM1, urophorphyrin methylase 1 | 0.68 | 0.01 | 0.76 | 0.04 |
| At5G41790 | CIP1, COP1-interactive protein 1 | 1.62 | 0.02 | 0.81 | 0.07 |
| At5G43180 | Protein of unknown function, DUF599 | 0.71 | 0.02 | 0.68 | 0.06 |
| At5G44720 | Molybdenum cofactor sulfurase family protein | 1.12 | 0.04 | 1.19 | 0.08 |
| At5G45510 | Leucine-rich repeat (LRR) family protein | 1.38 | 0.08 | 0.79 | 0.07 |
| At5G47730 | Sec14p-like phosphatidylinositol transfer family protein | 1.19 | 0.06 | 1.41 | 0.09 |
| At5G48290 | Heavy metal transport/detoxification superfamily protein | 1.67 | 0.46 | 1.45 | 0.17 |
| At5G48930 | HCT, hydroxycinnamoyl-CoA shikimate/quinate hydroxycinnamoyl transferase | 2.04 | 0.17 | 1.16 | 0.07 |
| At5G49770 | Leucine-rich repeat protein kinase family protein | 0.75 | 0.1 | 1.78 | 0.1 |
| At5G53140 | Protein phosphatase 2C family protein | 1.14 | 0.08 | 0.82 | 0.03 |
| At5G53330 | Ubiquitin-associated/translation elongation factor EF1B protein | 1.21 | 0.06 | 1.21 | 0.12 |
| At5G53480 | ARM repeat superfamily protein | 1.06 | 0.01 | 0.87 | 0 |
| At5G54040 | Cysteine/Histidine-rich C1 domain family protein | 0.82 | 0.04 | 1.54 | 0.24 |
| At5G54160 | ATOMT1, OMT1, O-methyltransferase 1 | 0.72 | 0.08 | 1.2 | 0.1 |
| At5G54670 | ATK3, KATC, kinesin 3 | 1.22 | 0.07 | 1.07 | 0.01 |
| At5G54680 | bHLH105, ILR3, basic helix-loop-helix (bHLH) DNA-binding superfamily protein | 1.32 | 0.11 | 1.29 | 0.11 |
| At5G54790 | Unknown protein | 3.2 | 0.75 | 2.02 | 0.19 |
| At5G54800 | ATGPT1, GPT1, glucose 6-phosphate/phosphate translocator 1 | 1.41 | 0.13 | 1.3 | 0.11 |
| At5G55560 | Protein kinase superfamily protein | 1.49 | 0.04 | 1.33 | 0.12 |
| At5G56660 | ILL2, IAA-leucine resistant (ILR)-like 2 | 1.36 | 0.19 | 1.18 | 0.04 |
| At5G57480 | P-loop containing nucleoside triphosphate hydrolases superfamily protein | 1.31 | 0.04 | 0.81 | 0.04 |
| At5G57540 | AtXTH13, XTH13, xyloglucan endotransglucosylase/hydrolase 13 | 1.53 | 0.11 | 2.33 | 0.36 |
| At5G60410 | ATSIZ1, SIZ1, DNA-binding protein with MIZ/SP-RING zinc finger, PHD-finger and SAP domain | 1.11 | 0.04 | 0.94 | 0.01 |
| At5G63790 | ANAC102, NAC102, NAC domain containing protein 102 | 1.18 | 0.08 | 1.19 | 0.07 |
| At5G63940 | Protein kinase protein with adenine nucleotide alpha hydrolases-like domain | 0.9 | 0.08 | 0.82 | 0.06 |
| At5G65640 | bHLH093, beta HLH protein 93 | 0.92 | 0.05 | 1.29 | 0.11 |

If the transcript of a gene was not determined (read number =zero) is one the biological repeats under control condition, no fold change was available in this repeat, resulting in no any number for the final mean, which indicated as ‘-’.
