# Supplementary material for: Genome-wide analysis of overlapping genes regulated by iron deficiency and phosphate starvation reveals new interactions in Arabidopsis roots
Source: BMC Res Notes. 2015 Oct 12;8:555. doi: 10.1186/s13104-015-1524-y (PMC4604098; doi:10.1186/s13104-015-1524-y)
Supplement: Supplementary file 7 — 10.1186/s13104-015-1524-y Gene ontology enrichment was assessed using GOBU (Lin et al. [66]) in the 210 overlapping genes comprising the largest model in Figure S2 (elim, P < 0.01). In the term type column, P, F and C indicate biological process, functional process and subcellular localization, respectively. [file 13104_2015_1524_MOESM7_ESM.doc]

**Additional file 7** Gene Ontology enrichment was assessed using GOBU (Lin et al., 2006) in the 210 overlapping genes comprising the largest model in Figure S2 (elim, P<0.01). In the term type column, P, F and C indicate biological process, functional process and subcellular localization, respectively.

| **GOID** | **Term Type** | **P-value(elim)** | **GO name** |
| --- | --- | --- | --- |
| GO:0019761 | P | 1.10E-07 | glucosinolate biosynthetic process |
| GO:0046686 | P | 1.34E-06 | response to cadmium ion |
| GO:0009651 | P | 2.51E-06 | response to salt stress |
| GO:0009098 | P | 1.97E-05 | leucine biosynthetic process |
| GO:0009611 | P | 5.07E-05 | response to wounding |
| GO:0010731 | P | 3.84E-04 | protein glutathionylation |
| GO:0006979 | P | 3.86E-04 | response to oxidative stress |
| GO:0009409 | P | 3.96E-04 | response to cold |
| GO:0065008 | P | 0.001432 | regulation of biological quality |
| GO:0050832 | P | 0.001479 | defense response to fungus |
| GO:0010043 | P | 0.001863 | response to zinc ion |
| GO:0005985 | P | 0.001863 | sucrose metabolic process |
| GO:0009620 | P | 0.002153 | response to fungus |
| GO:0042742 | P | 0.002353 | defense response to bacterium |
| GO:0006075 | P | 0.002462 | 1,3-beta-D-glucan biosynthetic process |
| GO:0009991 | P | 0.002896 | response to extracellular stimulus |
| GO:0009407 | P | 0.002997 | toxin catabolic process |
| GO:0006813 | P | 0.003187 | potassium ion transport |
| GO:0042221 | P | 0.003204 | response to chemical stimulus |
| GO:0009719 | P | 0.003255 | response to endogenous stimulus |
| GO:0000103 | P | 0.003367 | sulfate assimilation |
| GO:0009615 | P | 0.003588 | response to virus |
| GO:0009870 | P | 0.003869 | defense response signaling pathway, resistance gene-dependent |
| GO:0006012 | P | 0.004403 | galactose metabolic process |
| GO:0010033 | P | 0.004902 | response to organic substance |
| GO:0009698 | P | 0.004981 | phenylpropanoid metabolic process |
| GO:0015794 | P | 0.00625 | glycerol-3-phosphate transport |
| GO:2000070 | P | 0.00625 | regulation of response to water deprivation |
| GO:0042306 | P | 0.00625 | regulation of protein import into nucleus |
| GO:0006429 | P | 0.00625 | leucyl-tRNA aminoacylation |
| GO:0010247 | P | 0.00625 | detection of phosphate ion |
| GO:0001778 | P | 0.00625 | plasma membrane repair |
| GO:0046740 | P | 0.00625 | spread of virus in host, cell to cell |
| GO:0006154 | P | 0.00625 | adenosine catabolic process |
| GO:0019760 | P | 0.006858 | glucosinolate metabolic process |
| GO:0009070 | P | 0.006858 | serine family amino acid biosynthetic process |
| GO:0052543 | P | 0.006858 | callose deposition in cell wall |
| GO:0044403 | P | 0.006858 | symbiosis, encompassing mutualism through parasitism |
| GO:0043900 | P | 0.007549 | regulation of multi-organism process |
| GO:0034220 | P | 0.007908 | ion transmembrane transport |
| GO:0016998 | P | 0.00827 | cell wall macromolecule catabolic process |
| GO:0045087 | P | 0.009158 | innate immune response |
| GO:0009411 | P | 0.009688 | response to UV |
| GO:0051707 | P | 0.009928 | response to other organism |
| GO:0008061 | F | 1.32E-05 | chitin binding |
| GO:0010177 | F | 3.89E-05 | 2-(2'-methylthio)ethylmalate synthase activity |
| GO:0004601 | F | 4.79E-05 | peroxidase activity |
| GO:0045174 | F | 2.31E-04 | glutathione dehydrogenase (ascorbate) activity |
| GO:0003824 | F | 3.69E-04 | catalytic activity |
| GO:0004568 | F | 4.42E-04 | chitinase activity |
| GO:0016671 | F | 6.30E-04 | oxidoreductase activity, acting on a sulfur group of donors, disulfide as acceptor |
| GO:0005200 | F | 8.63E-04 | structural constituent of cytoskeleton |
| GO:0043295 | F | 0.001692 | glutathione binding |
| GO:0004866 | F | 0.001863 | endopeptidase inhibitor activity |
| GO:0004034 | F | 0.00206 | aldose 1-epimerase activity |
| GO:0020037 | F | 0.002425 | heme binding |
| GO:0003843 | F | 0.002462 | 1,3-beta-D-glucan synthase activity |
| GO:0016207 | F | 0.002898 | 4-coumarate-CoA ligase activity |
| GO:0015077 | F | 0.003232 | monovalent inorganic cation transmembrane transporter activity |
| GO:0045309 | F | 0.003367 | protein phosphorylated amino acid binding |
| GO:0004364 | F | 0.003384 | glutathione transferase activity |
| GO:0008483 | F | 0.004245 | transaminase activity |
| GO:0047763 | F | 0.00625 | caffeate O-methyltransferase activity |
| GO:0004573 | F | 0.00625 | mannosyl-oligosaccharide glucosidase activity |
| GO:0015169 | F | 0.00625 | glycerol-3-phosphate transmembrane transporter activity |
| GO:0047172 | F | 0.00625 | shikimate O-hydroxycinnamoyltransferase activity |
| GO:0050421 | F | 0.00625 | nitrite reductase (NO-forming) activity |
| GO:0047205 | F | 0.00625 | quinate O-hydroxycinnamoyltransferase activity |
| GO:0033799 | F | 0.00625 | myricetin 3'-O-methyltransferase activity |
| GO:0030755 | F | 0.00625 | quercetin 3-O-methyltransferase activity |
| GO:0047889 | F | 0.00625 | ferredoxin-nitrate reductase activity |
| GO:0004851 | F | 0.00625 | uroporphyrin-III C-methyltransferase activity |
| GO:0016831 | F | 0.006338 | carboxy-lyase activity |
| GO:0046872 | F | 0.006629 | metal ion binding |
| GO:0005886 | C | 2.64E-12 | plasma membrane |
| GO:0048046 | C | 1.07E-06 | apoplast |
| GO:0005829 | C | 2.85E-06 | cytosol |
| GO:0005773 | C | 0.002323 | vacuole |
| GO:0000148 | C | 0.002462 | 1,3-beta-D-glucan synthase complex |
| GO:0005618 | C | 0.002701 | cell wall |
| GO:0045298 | C | 0.002898 | tubulin complex |
| GO:0005955 | C | 0.00625 | calcineurin complex |
| GO:0009570 | C | 0.008579 | chloroplast stroma |
| GO:0005774 | C | 0.009232 | vacuolar membrane |
