# Supplementary material for: Genome-wide analysis of overlapping genes regulated by iron deficiency and phosphate starvation reveals new interactions in Arabidopsis roots
Source: BMC Res Notes. 2015 Oct 12;8:555. doi: 10.1186/s13104-015-1524-y (PMC4604098; doi:10.1186/s13104-015-1524-y)
Supplement: Supplementary file 9 — 10.1186/s13104-015-1524-y Subset of 35 overlapping genes induced by iron deficiency with fold change more than 1.5-fold but down-regulated by Pi starvation the Arabidopsis roots (P < 0.05). The fold change of the gene expression was indicated as mean with standard deviation (SD). [file 13104_2015_1524_MOESM9_ESM.doc]

**Additional file 9** Subset of 35 overlapping genes induced by iron deficiency with fold change more than 1.5-fold but down-regulated by Pi starvation the Arabidopsis roots (P<0.05). The fold change of the gene expression was indicated as mean with standard deviation (SD).

| 列**AGI** | 列2 **Annotation** | **Mean(Fe-/Fe+)** | SD | **Mean(Pi-/Pi+)** | **SD** |  |
| --- | --- | --- | --- | --- | --- | --- |
| At3G12900 | 2-Oxoglutarate (2OG) and Fe(II)-dependent oxygenase superfamily protein | 612.44 | 199.47 | 0.06 | 0.10 |  |
| At4G31940 | CYP82C4, cytochrome P450, family 82, subfamily C, polypeptide 4 | 184.70 | 3.63 | 0.08 | 0.01 |  |
| At4G19690 | ATIRT1, IRT1, iron-regulated transporter 1 | 54.72 | 7.88 | 0.26 | 0.04 |  |
| At1G73120 | Unknown protein | 8.98 | 2.09 | 0.28 | 0.11 | |
| At1G01580 | ATFRO2, FRD1, FRO2, ferric reduction oxidase 2 | 59.73 | 10.56 | 0.35 | 0.05 |  |
| At3G46900 | COPT2, copper transporter 2 | 71.15 | 33.39 | 0.40 | 0.36 |  |
| At3G21500 | DXPS1, 1-deoxy-D-xylulose 5-phosphate synthase 1 | 3.76 | 1.94 | 0.40 | 0.26 |  |
| At5G38820 | Transmembrane amino acid transporter family protein | 54.35 | 11.69 | 0.43 | 0.09 |  |
| At4G00910 | Aluminium activated malate transporter family protein | 1.66 | 0.13 | 0.46 | 0.22 |  |
| At2G33020 | AtRLP24, RLP24, receptor like protein 24 | 5.05 | 1.74 | 0.48 | 0.33 |  |
| At2G02310 | AtPP2-B6, PP2-B6, phloem protein 2-B6 | 5.30 | 2.01 | 0.49 | 0.12 |  |
| At5G04950 | ATNAS1, NAS1, nicotianamine synthase 1 | 4.39 | 0.50 | 0.49 | 0.01 |  |
| At4G22980 | Pyridoxal phosphate (PLP)-dependent transferases superfamily protein (TAIR:AT5G51920.1) | 2.30 | 0.28 | 0.56 | 0.14 |  |
| At5G05250 | Unknown protein | 6.62 | 0.95 | 0.57 | 0.10 |  |
| At5G19970 | Unknown protein | 2.81 | 0.43 | 0.57 | 0.07 |  |
| At1G78230 | Outer arm dynein light chain 1 protein | 2.51 | 0.57 | 0.59 | 0.22 |  |
| At1G74770 | Zinc ion binding | 6.43 | 0.50 | 0.66 | 0.07 |  |
| At5G02780 | GSTL1, glutathione transferase lambda 1 | 57.98 | 10.87 | 0.69 | 0.06 |  |
| At3G12977 | NAC (No Apical Meristem) domain transcriptional regulator superfamily protein | 1.56 | 0.26 | 0.70 | 0.15 |  |
| At3G15510 | ANAC056, ATNAC2, NAC2, NARS1, NAC domain containing protein 2 | 2.13 | 0.62 | 0.71 | 0.15 |  |
| At2G21540 | ATSFH3, SFH3, SEC14-like 3 | 1.89 | 0.09 | 0.71 | 0.13 |  |
| At3G56980 | BHLH039, ORG3, basic helix-loop-helix (bHLH) DNA-binding superfamily protein | 34.23 | 13.48 | 0.73 | 0.18 |  |
| At3G12920 | SBP (S-ribonuclease binding protein) family protein | 1.77 | 0.23 | 0.75 | 0.04 |  |
| At3G18290 | BTS, EMB2454, zinc finger protein-related | 2.51 | 0.14 | 0.76 | 0.09 |  |
| At5G26820 | ATIREG3, IREG3, IREG3, MAR1, RTS3, iron-regulated protein 3 | 2.54 | 0.28 | 0.77 | 0.19 |  |
| At1G18910 | Zinc ion binding;zinc ion binding | 3.33 | 0.13 | 0.78 | 0.04 |  |
| At1G14190 | Glucose-methanol-choline (GMC) oxidoreductase family protein | 2.29 | 0.41 | 0.78 | 0.08 |  |
| At1G24320 | Six-hairpin glycosidases superfamily protein | 2.26 | 0.27 | 0.78 | 0.09 |  |
| At1G47565 | Transposable element gene | 1.60 | 0.29 | 0.80 | 0.07 |  |
| At5G41790 | CIP1, COP1-interactive protein 1 | 1.62 | 0.02 | 0.81 | 0.07 |  |
| At2G14210 | AGL44, ANR1, AGAMOUS-like 44 | 2.01 | 0.37 | 0.82 | 0.09 |  |
| At1G48300 | Unknown protein | 2.27 | 0.25 | 0.85 | 0.07 |  |
| At5G22800 | EMB1030, EMB263, EMB86, Alanyl-tRNA synthetase, class IIc | 1.62 | 0.21 | 0.85 | 0.10 |  |
| At1G76950 | PRAF1, Regulator of chromosome condensation (RCC1) family with FYVE zinc finger domain | 1.58 | 0.04 | 0.89 | 0.05 |  |
| At5G53850 | Haloacid dehalogenase-like hydrolase family protein | 2.09 | 0.06 | 0.96 | 0.01 |  |
