# Supplementary material for: Genome-wide analysis of overlapping genes regulated by iron deficiency and phosphate starvation reveals new interactions in Arabidopsis roots
Source: BMC Res Notes. 2015 Oct 12;8:555. doi: 10.1186/s13104-015-1524-y (PMC4604098; doi:10.1186/s13104-015-1524-y)
Supplement: Supplementary file 10 — 10.1186/s13104-015-1524-y Primers used in this study. [file 13104_2015_1524_MOESM10_ESM.doc]

**Additional file 10** Primers used in this study

| **Name** | **Primer sequence** | **Targets** |
| --- | --- | --- |
| IRT1F | 5’CACCATTCGGAATAGCGTTAGG3’ | IRT1(AT4G19690) |
| IRT1R | 5’CCAGCGGAGCATGCATTTA3’ |  |
| FRO2F | 5’GGCCACCACATATCCGAAGAT3’ | FRO2(AT1G01580) |
| FRO2R | 5’CGACGTGGAGGACAAAGAAGAG3’ |  |
| CYP82C4F | 5’TAACCATTCCTAAAGCCACGC3’ | CYP82C4(AT4G31940) |
| CYP82C4R | 5’CGTTTACGAACATAATACCGCAC3’ | |
| PHR1F | 5’CCAGAAGAAGAAACCAGGAA3’ | PHR1(AT4G28610) |
| PHR1R | 5’ACTCCGAGATTATCCAGCAA3’ |  |
| EF1F | 5’GCTGTTCGTGGTGTTGAGATGC3’ | EF1-β2(AT5G19510) |
| EF1R | 5’AGGCTCTGAGGTGAGGAAGTCT3’ |  |
| FITF | 5’TTTTCGCGGTATCAATCCTC3’ | FIT(AT2G18160) |
| FITR | 5’GGTATGTGTCCGGAGAAGGA3’ |  |
| SPX1F | 5’ GATTCCATTGTTGGAGCAAGA3’ | SPX1(AT5G20150) |
| SPX1R | 5’ AATCTGTTAGCTTCTTCTATTGTA3’ |  |
| PYEF | 5’ CAGGACTTCCCATTTTCCAA3’ | PYE(AT3G47640) |
| PYER | 5’ CTTGTGTCTGGGGATCAGGT3’ |  |
| AT1G74790F | 5’ AGGAAACTATGGTTGGCGTG3’ | AT1G74790 |
| AT1G74790R | 5’ GCTGATTTTCCGCTTGAGTC3’ |  |
